# Thermal reaction of the subsurface on the operation of a geothermal planar trench collector

**DOI:** 10.1038/s41597-025-06072-8

**Published:** 2025-10-20

**Authors:** Adinda Van de Ven, Fabian Neth, Andreas Köhler, Anna Albers, Linda Schindler, Daniel Buchmiller, Marion Denninger, Hagen Steger, Roman Zorn, Roland Koenigsdorff

**Affiliations:** 1 Institute for Building and Energy Systems, Biberach University of Applied Sciences, Karlstraße 11, 88400 Biberach, Germany; 2 Institute of Applied Geosciences, Karlsruhe Institute of Technology, Kaiserstraße 12, 76131 Karlsruhe, Germany; 3 European Institute for Energy Research (EIFER), Emmy-Noether-Straße 11, 76131 Karlsruhe, Germany

**Keywords:** Mechanical engineering, Geothermal energy

## Abstract

In order to develop and analyse ground heat collectors, experimental plants with reliable measurement data are needed. Here, an experimental plant with a planar ground heat collector installed vertically in a trench is presented. The ground heat collector is extensively equipped with measurement technology installed to track its thermal behaviour and that of the surrounding ground. This includes 3 different types of temperature measurements as well as the determination of the volumetric water content and the bulk electrical conductivity of the subsurface near the collector. The description also includes detailed information on the geometry of the experimental plant and the sensors installed, the data acquisition systems used and information on measurement accuracy and calibration procedures. Measurement data collected by these sensors during a thermal response test on the planar ground heat collector are described.

## Background & Summary

Ground-source heat pumps are becoming increasingly important as a fossil-free and sustainable alternative for domestic heating^1^. Besides the more widely applied borehole heat exchangers, horizontal ground heat collectors also represent a viable source configuration for heat pump systems. A wide range of geometrical configurations is available for ground heat collector, ranging from horizontally laid pipes and spiral or basket-shaped configurations to planar systems installed in trenches. This diversity results in differences in thermal behaviour during operation and the corresponding need for an equally wide range of design models. For both numerical and analytical models, validation against measurement data is essential to demonstrate their practical applicability. It is necessary to quantify uncertainties in modelling and to understand real (thermal) behaviour of a plant, which is helpful while developing operational strategies.

Whereas for linear-loop and slinky-coil configurations there exist measurement data from case studies, experimental plants or laboratory scale experiments^2–11^, little data exist for other configurations^12–17^. Currently, only two studies focusing on experimental data of planar trench collectors are documented in the literature, one experimental plant in Ferrara (Italy)^13^ and one small-scale test rig^16^. The experimental plant in Ferrara consists of two planar trench collectors, which can be operated separately, in parallel or in series and are coupled to a heat pump. An irrigation system allows to wet the soil on demand and the surrounding subsurface and the pipes are equipped with several temperature sensors^13^. However, soil moisture is not measured here. The small-scale test rig is similar to the experimental plant in Ferrara but on the basis of 1:10. Two boxes filled with sand and a flat-panel collector in the middle are used for thermal performance evaluations for various operational modes and soil conditions^16^.

**Fig. 1:**
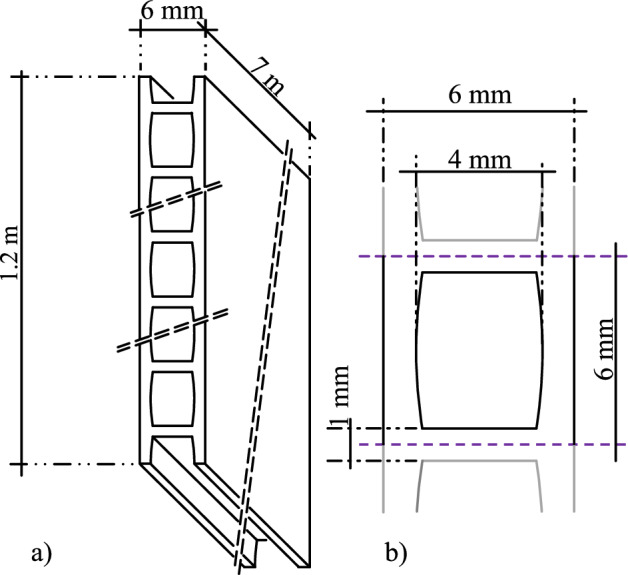
A sketch of (**a**) the inner geometry incl. a perspective of the entire collector and its dimensions and (**b**) a detailed representation of a collector channel.

**Fig. 2:**
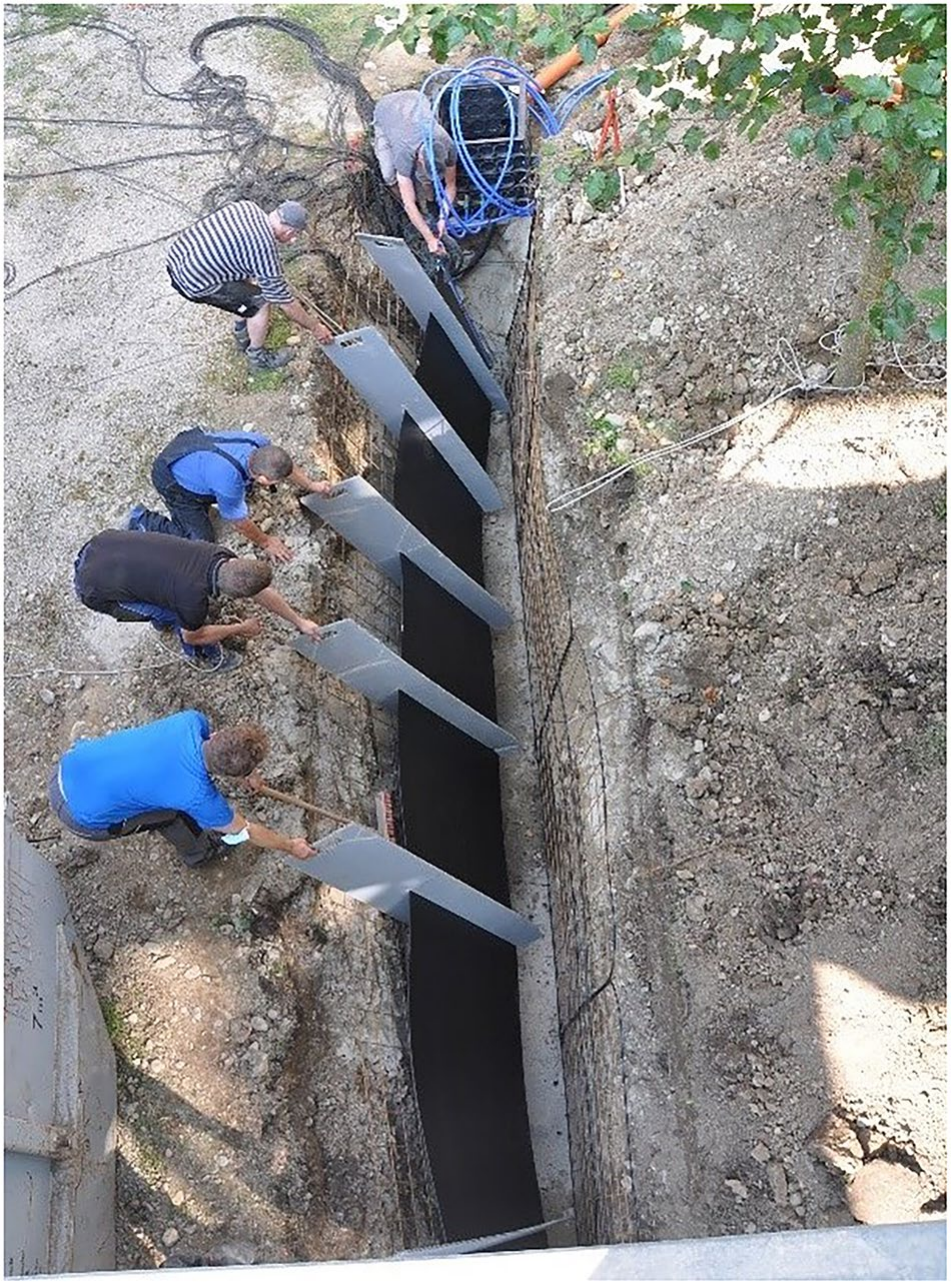
Installation of the planar collector into the trench.

**Fig. 3:**
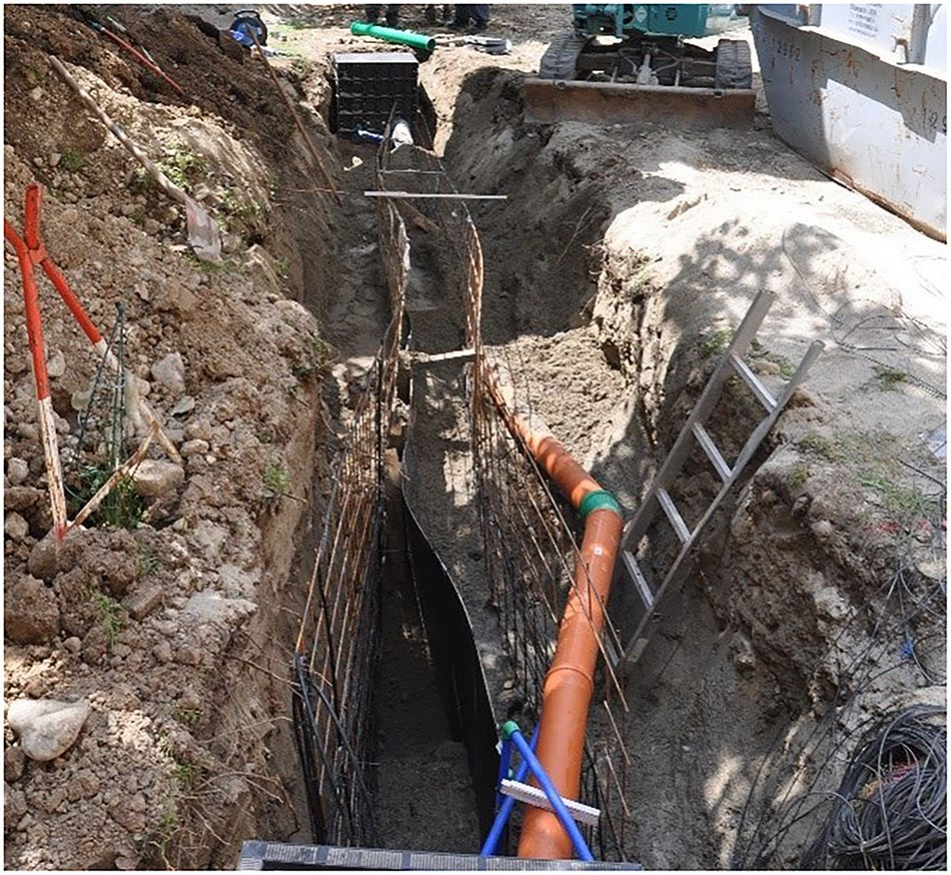
Installation of the collector, the structural steel mats and the connecting pipes.

However, easily accessible and extensive measurement data of ground heat collectors are rare. With this data descriptor, we present a planar trench collector located in the geothermal test field of Biberach University of Applied Sciences in Germany, Karlstraße 11, in 88400 Biberach. It was installed during the QEWSplus research project (www.qewsplus.de/english/). With a height of 1.2 m, a length of 7 m and a thickness of 6 mm, the collector is placed vertically in a trench and covered by a soil layer of 70 cm. The collector consists of a polypropylene plate with 199 small channels inside. 189 of these channels are active, i.e. they are flowed through during operation, while the 5 upper and 5 lower channels are inactive due to safety reasons. A sketch of the collector and its channels is shown in Fig. 1. The connecting pipes attached to the collector have an outer diameter of 25 mm. The installation of the collector in the trench is shown in Fig. 2. The unstable ground at the site resulted in a risk of the trench collapsing during installation. In addition, larger rocks in the ground made it difficult to create a vertical and straight trench. So, the trench had to be wider than usual. Despite spacers, it was not possible to install the collector plate centrally along the entire length of the trench, and as a result, it could not be filled uniformly with sand. This led to bulges, which prevented the collector from being installed straight, see Fig. 3. Therefore, the actual installation position of the collector and the installed measurement sensors was traced as far as possible and documented in drawings and metafiles published together with measurement results in the following repository: https://zenodo.org/records/14069578^18^.

Two shafts, one at the collector inlet and one at the collector outlet, are installed to enable direct access to the collector inlet and outlet pipes. From the collector outlet shaft 2 pipes lead back to the plant and pass through the shaft next to the inlet. One of these pipes is insulated and the other one is non-insulated, which allows to investigate the thermal influence of connecting pipes of geothermal heat exchangers. The collector is flown through with brine, consisting of a monoethylene-water mixture of 41.5% glycol for the duration of the research project QEWSplus (until the end of 2024). Once the project is completed, the glycol content will be reduced due to water law requirements.

To characterise the subsurface and the backfill sand, both *in-situ* measurements were carried out and samples were taken and analysed in the laboratory. The thermal conductivity was found to range between 1.3 and 2.0 W/(m·K). From a series of measurements in which the water content and packing density of the backfill sand were varied, porosity values between 30 and 40% were determined. The specific heat capacity *c*_*p*_ ranges from 660 to 1,410 J/(kg·K) within a temperature range of −10 °C to 40 °C. This variation is primarily due to differences in soil type and associated moisture content. The topsoil had a moisture content of 15.10%, which was more than twice that of the fill sand (6.10%) and the *in-situ* soil (ranging from 5.15% to 7.40%). At 20 °C, the *c*_*p*_ value of the *in-situ* soil lies between 950 and 1,050 J/(kg·K), while it reaches approximately 1,280 J/(kg·K) for the topsoil and 860 J/(kg·K) for the backfill sand.

Furthermore, all datasets include measurements taken prior to heat injection or extraction, which can be used to determine the undisturbed subsurface temperature at the time of investigation to tackle the challenge of seasonally varying undisturbed temperatures influenced by solar radiation and other weather conditions. Note, that the undisturbed subsurface temperatures change very slowly compared to the duration of the thermal response test which allows to take values prior to the test as reference for undisturbed conditions. Similarly, the moisture measurement before and during thermal activation of the trench collector allows to analyse any possible change in moisture due to heat injection or extraction in its vicinity.

Parts of the dataset described in this paper were already used to validate an analytical model for planar trench collectors in Van de Ven, *et al*.^19^. Apart from the analyses of the thermal behaviour of the trench collector while under operation, datasets of this plant of periods without active heat injection or extraction are helpful to analyse the temperature development in the subsurface in the experimental site.

## Methods

### The experiments

With this data descriptor, four datasets containing measurement values from April 28^th^ 2022 to May 3^rd^ 2022^18^, February 28^th^ 2023 to March 6^th^ 2023^20^, April 6^th^ 2023 to April 13^th^ 2023^21^ and Mai 2^nd^ 2024 to May 12^th^ 2024^22^ are published. Table 1 shows an overview of the four datasets. Within the first three periods, a thermal response test (TRT) with constant heat injection was carried out^23,24^. As mentioned above, the collector was operated with a monoethylene-water mixture of 41.5%. A TRT-device was directly connected to the inlet and outlet pipes in both collector shafts. The volume flow is determined using a high-precision magnetic inductive measuring device Krohne Ekoflux 020 K/D. The temperatures are measured directly at the transition to the collector using high-precision calibrated sensors. The heat output introduced is calculated in accordance with DIN EN 1434 using the values of the glycol mixture filled into the automation system.

**Table 1:** Overview of the datasets.

Description	Unit	Dataset 1^18^	Dataset 2^20^	Dataset 3^21^	Dataset 4^22^
Date of the dataset	DD.MM.YYYY	28.04.2022-03.05.2022	28.02.2023-06.03.2023	06.04.2023-13.04.2023	02.05.2024-12.05.2024
Heat injection/extraction rate	kW	0.88 ± 0.03407	1.51 ± 0.03569	1.54 ± 0.03577	−0.7 ± 0.01895
Volume flow	m^3^/h	1.00 ± 0.00739	0.88 ± 0.00725	0.88 ± 0.00725	0.35 ± 0.00673

The first test started with a fluid circulation at 15:25 on April 28^th^ 2022 for about 12 minutes with a constant volume flow of 1.00 m³/h without heating. At 15:38 on April 28^th^ the collector was loaded with both a constant heat injection rate of 0.88 kW (105 W/m²) and a constant volume flow of 1.00 m³/h. This heat injection rate and volume flow were continued until May 3^rd^ 2022, leading to a temperature increase of the fluid up to about 27 °C. This dataset is freely accessible at: https://zenodo.org/records/140695781^18^.

Similar to the first dataset, the second dataset started with a fluid circulation on February 28^th^ 2023 at 13:48 until 13:54 with a constant volume flow of 0.88 m³/h. Immediately thereafter the heat injection is started with a rate of 1.51 kW (180 W/m²). This heat injection rate was continued until March 6^th^ 2023 at 07:06, whereas the fluid circulation was stopped at March 6^th^ 2023 at 08:39. This dataset is freely accessible at: https://zenodo.org/records/15369421^20^.

The third dataset is also a thermal response test with a constant heat injection rate, which started its fluid circulation on April 6^th^ 2023 at 14:49 with a constant volume flow of 0.88 m³/h. The constant heat injection rate was started on April 6^th^ 2023 at 15:00 with 1.54 kW (183 W/m²). On April 11^th^ 2023 at 07:49 the heat injection was stopped, whereas the fluid was circulated for two more days until April 13^th^ 2023 15:26. This dataset is freely accessible at: https://zenodo.org/records/15369554^21^.

The fourth dataset differs from the previous three datasets as this dataset contains measurement data collected during a cooling experiment, in which heat was extracted from the ground. Unfortunately, the compressor of the cooling system ceased operation despite continued requests from the control system on May 11^th^ 2024 around 18:50. Due to the presence of a buffer tank and the continued volume flow, the exact time of compressor failure could not be traced back. From May 2^nd^ at 15:57 onwards the fluid was circulated until the end of the dataset (May 12^th^ 2024) with a volume flow of 0.35 m³/h. Heat extraction started at the same time with a rate of about −0.7 kW (83 W/m^2^). This dataset is freely accessible at: https://zenodo.org/records/15425180^22^.

### Temperature measurement

Subsurface temperatures are logged at various depths by PT 100 sensors, some of which are directly attached to the collector, as well as by a fibre-optic cable and by thermistors within the moisture sensors. Along both sides of the collector, within the trench, there are structural steel mats installed: one with PT 100 sensors (on the right side in the direction of flow) and another with both PT 100 sensors and a fibre optic cable attached to it (on the left side in the direction of flow). Similar to the installation of the collector, the structural steel mats could not be installed straight either, leading to varying distances between the collector and the installed sensors. However, the exact position of the sensors and the collector are documented in pictures (see Fig. 3), drawings^18^ and in metafiles^18,20–22^. In the metafile of the PT 100-sensors, the shortest distance (i.e. the length of the straight line perpendicular from the plate surface to a sensor) between each PT 100 sensor and the collector itself is documented. Beyond that, on the left side in the direction of flow of the collector further PT 100 sensors in three different depths and five positions along the collector have been rammed into the subsurface at a distance of ca. 50 cm from the middle of the trench. On the other side, further 15 PT 100 sensors are rammed into the subsurface to monitor the subsurface temperature in detail. The thermistors are placed in the centre needle of all 5 moisture sensors. The exact position of the various sensors and their corresponding designation can be found in the drawings in the repository^18^ as well as in the metafiles^18,20–22^.

#### PT 100 measurement

The method described in this subchapter refers to the datasets named “*20220428-20220503_PT100_calibrated.csv”*^18^, “20230228-20230306_PT100_calibrated.csv”^20^, “230406-230413_PT100_calibrated.csv”^21^ and “20240502-20240512_PT100_calibrated.csv”^22^.

**Fig. 4:**
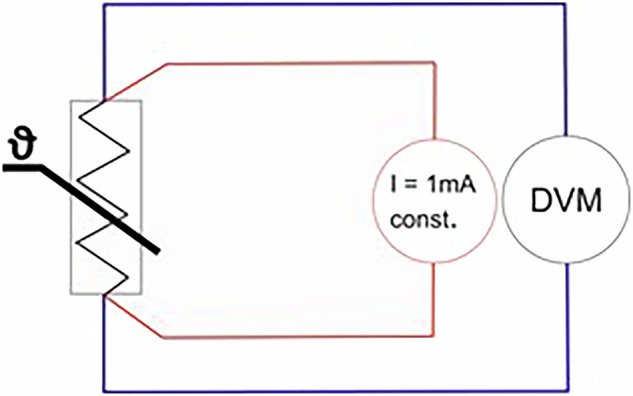
The wiring diagram of a four-wire resistance measurement.

A resistance temperature detector (RTD) measures temperature via the electrical resistance of a certain material. The materials used – here: platinum - have a well-known resistance/temperature relationship that is used to indicate the temperature. The PT 100 RTDs have a high linearity within a certain temperature range (e.g. −20 °C to 85 °C). When a RTD is connected with a two-wire installation, the measured resistance includes the lead resistance which results in significant measurement errors for long connecting wires. To avoid this error, the four-wire technology is used, i.e. the sensor is connected to two circuits. In Fig. 4, the two electrical circuits are displayed in different colours. The red circuit includes an active 1 mA constant current generator. Because of the constant current, there is a specific voltage drop at the RTD. This voltage drop depends on the resistance (ohm’s law) of the RTD for a specific temperature. This voltage can be measured with the circuit shown in blue and a digital voltage meter (DVM) at a very high internal resistance of the voltmeter, nearly without any current and an influence of the wire resistances.

With these measured voltages and the measured current of the constant current generator, the resistance of the RTD can easily be calculated with Ohm’s Law. In our case, the constant current is also measured and used as input to the following formula:

1 $$R=\frac{U}{I}$$

The measurement data was determined in accordance with DIN 43760 until the July 2^nd^ 2024, after that DIN EN IEC 60751 2023-06 was used. In DIN EN IEC 60751 2023-06 empirical formulas are introduced to calculate the resistance of an RTD from a temperature in two temperature ranges from −200 °C to 0 °C and from 0 °C to 850 °C. The standard approach involves interpolating the temperature from the measured resistance between the closest tabulated values using formulas and the resulting reference tables. However, in the current version of the international standard, these reference tables are now only for informational purposes. The temperature values can be calculated directly using the formulas in^25^ if they are solved according to temperature. Since the formula for negative temperatures is quartic, it cannot be easily solved according to temperature for an exact solution. Therefore, the numerical Newton-Raphson method is used for the conversion, applying Eq. (2) for temperatures above 0 °C and Eq. (3) for temperatures below 0 °C. The following formulas apply for the specified temperature ranges^25^:

2 $${R}_{T}={R}_{0}\cdot (1+AT+B{T}^{2})\,for\,T\ge 0^\circ C$$

3 $${R}_{T}={R}_{0}[1+AT+B{T}^{2}+C\cdot (T-100^\circ C)\cdot {T}^{3}]\,for\,T < 0^\circ C$$

With:

4 $$A=3.90833\cdot {10}^{-3}{^\circ C}^{-1}$$

5 $$B=-5.775\cdot {10}^{-7}{^\circ C}^{-2}$$

6 $$C=-4.183\cdot {10}^{-12}{^\circ C}^{-4}$$

7 $${R}_{0}:{resistance}\,{at}\,{T}{=}0^\circ {C}\,{in}\,{\Omega }$$

8 $${R}_{T}:{resistance}\,{at}\,{temperature}\,{T}{=}{{T}}_{{R}_{T}}\,in\,{\Omega }$$

The PT 100 sensors are calibrated together with all components of the measurement chain, i.e. all cables, terminal points and transmitters as in regular operation mode. The calibration is carried out with the calibration device LAUDA ecoline/staredition RE 212 cooling thermostat for several points within the range of −5 °C to 30 °C. At each calibration point, ten measured values are recorded, which are used to calculate its average value. A high-precision thermometer (PHYSICS 1000) is used as a calibration standard to evaluate the deviation of the measured values from this standard. Using these deviations, the calibration function is determined by linear regression. The gain *m* and the offset *b* of the regression curve are determined to be able to correct the measured value ${T}_{{\rm{m}}{\rm{e}}{\rm{a}}{\rm{s}}}$ according to Eq. (9).

9 $${T}_{{\rm{c}}{\rm{o}}{\rm{r}}}=(1+m)\cdot {T}_{{\rm{m}}{\rm{e}}{\rm{a}}{\rm{s}}}+b$$

The PT 100 values provided here already include the correction of the calibration as described above. The gain *m* and the offset *b* used to correct the data provided here can be found for each PT 100 sensor both in the supplementary material—in the diagrams, where they are shown in the equation of the linear regression line—and in the metafile^18^.

Additionally, the calibration curve, the variance and the degrees of freedom in accordance to JCGM 100:2008^26^ are depicted in an extra text box in the calibration diagrams in the supplementary material, as well as the combined standard uncertainty composed of the uncertainty of the reference temperature sensor and the overall uncertainty of the calibration curve. As the corrected temperature according to Eq. (9) and the calibration method in accordance to JCGM 100:2008^26^ lead to the same temperature, the combined standard uncertainty in accordance to JCGM 100:2008^26^ also applies for the measurement data as published. Due to its narrow width, the uncertainty band associated with the combined standard uncertainty has been omitted from the diagrams.

On August 6^th^ 2024, 2 additional PT 100 sensors were installed to measure the temperature at the subsurface: GHC1_T_001_L017_778 is located underneath an overpass bridge connecting the two adjacent buildings and GHC1_T_001_R004_072 is located 72 cm from the inlet shaft and is not covered by the overpass.

The temperature sensors assigned to the heating-cooling unit are not included in the plans, as these sensors are positioned directly at the inlet and outlet of the heating-cooling unit within the building. For the described data, the heating-cooling unit was not used, since the heat input was supplied by a TRT device. Therefore, the description of the heating-cooling unit is not part of this data descriptor. However, the two sensors located at the inlet and outlet of the heating-cooling unit are measured via the same measurement technology as the screw-in and ground sensors. Consequently, these two measurement points are included in the dataset.

#### Fibre optic measurement

**Fig. 5:**
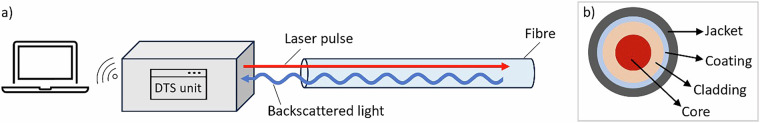
(**a**) Measurement setup, (**b**) Fibre cable structure.

The method described in this subchapter refers to the dataset named “*20220428-20220503_FiberOpticMeasurement.csv”*^18^. Temperature is measured using Distributed Temperature Sensing (DTS). A DTS unit sends laser pulses through an optical fibre that are partially backscattered and analysed at the transmitter end Fig. 5a). The backscattered signal is distributed over a range of wavelengths that can be distinguished into different signals, such as Rayleigh, Raman, or Brillouin signals. To determine the temperature along the fibre, the amplitude of the Raman signal is analysed. The ratio of the Raman anti-Stokes signal, which is strongly temperature dependent, to the Raman Stokes signal, which is weakly temperature dependent, is calculated by the DTS unit. The local position of the temperature within the fibre cable is determined by measuring the time of arrival of the backscattered signal. The length of the laser pulse determines the minimum distance between the samples. The shorter the pulse length (several nanoseconds), the shorter the sampling interval of the temperature readings. Note that the measured temperature value is more of an average value along the fibre, influenced by the selected spatial resolution.

Both ends of the fibre cable are connected to the DTS unit for double end measurements. The laser pulses alternate between the two ends of the cable, resulting in higher accuracy in the centre of the cable. In contrast, when only a single end of the cable is connected to the DTS unit, the measurement accuracy decreases along the length of the fibre cable.

An optical fibre consists of a fibre core and several protective layers, namely a cladding, a coating, and a jacket (Fig. 5b). In this setup, a multimode fibre with a 50 µm core is selected.

**Fig. 6:**
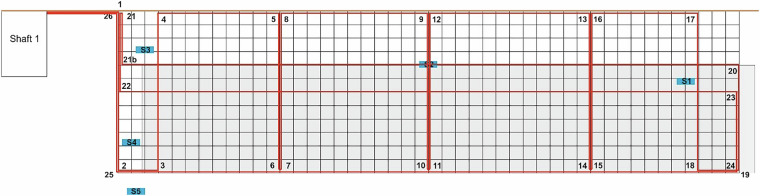
Sketch showing the position of the fibre optic cable along the steel mat. The fibre optic cable is laid along the positions (numbers); the approximate relative position of the collector is marked in grey and S1 to S5 are moisture sensors (see chapter 2.3 and 3.3).

For continuous temperature measurement, a 110.5 m long fibre optic cable (Helucom A-DQ(ZN)B2Y 4 G50/125) is fixed to the structural steel mat with cable ties (see also section 2.2.2). The steel mat measures 7 m × 1.8 m and the collector measures 7 m × 1.2 m (Fig. 6). The horizontal distance between the collector and the structural steel mat is not uniform and varies from approximately ~10 cm to ~40 cm.

The two ends of the fibre optic cable are connected to an Agilent AP Sensing instrument (model: N4386B, serial number: DE47504176). The temperature is measured from April 28^th^ 2022 00:01:00 to May 3^rd^ 2022 23:59:00. The DTS Configurator software is used to perform the measurement.

**Fig. 7:**
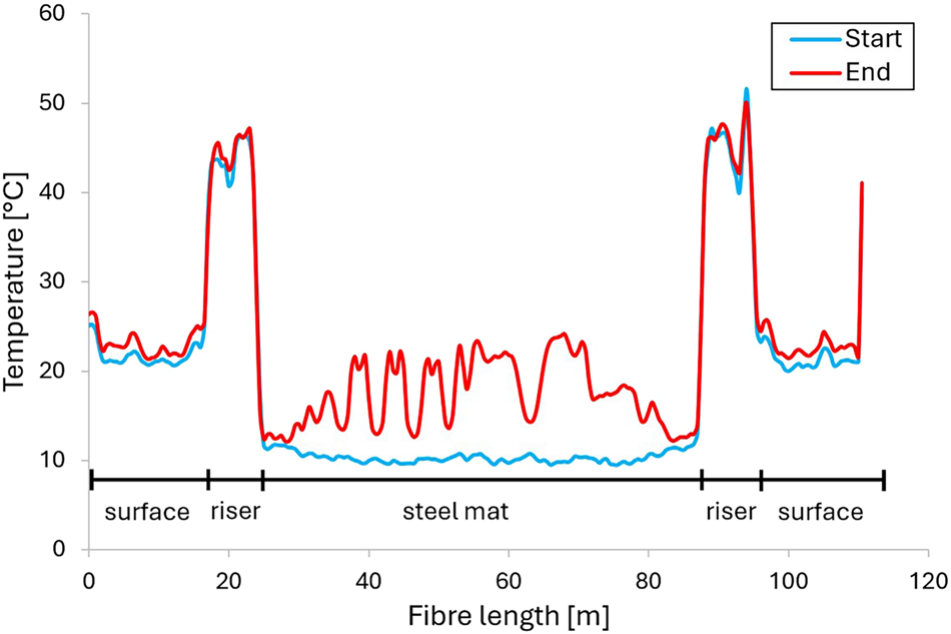
Temperature along the whole fibre optic cable at the beginning and end of heating phase.

The black unsheathed fibre optic cable is partially exposed to the air and is routed in a riser attached to the exterior wall of the building. The high temperatures, reaching peaks of up to 50 °C, are directly influenced by the solar radiation striking the riser (see Fig. 7). The fibre optic cable is shorter than 110.5 m, with the result that the signal is no longer resolved correctly by the measurement devise at distances larger than 110.5 m. Therefore, these measurement values should be ignored.

**Fig. 8:**
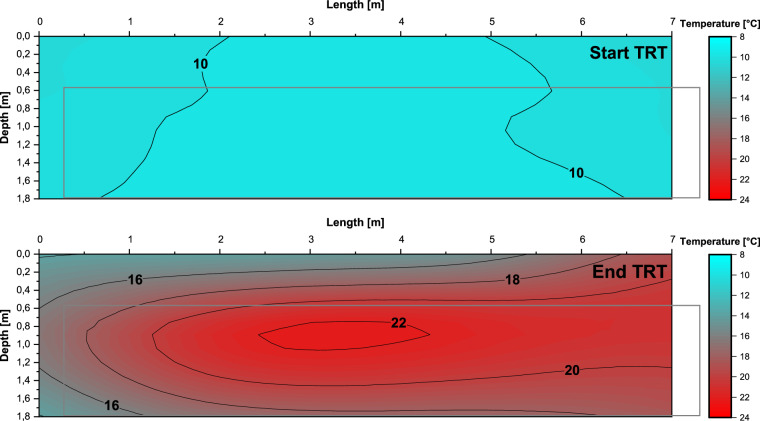
Contour plot of the DTS measurements of the fibre optic cable mounted at the steel mat at the beginning and end of the TRT.

Contour plots (Fig. 8) can be generated using the position of the fibre optic cable (Fig. 6).

The temperature distribution along the fibre optic cable reflects the heating up of the collector and the surrounding underground, as well as the different distances between the collector and the steel mat. The challenging installation conditions made it difficult to clearly assign the measurement positions along the fibre optic cable. Nevertheless, a reasonable temperature distribution is obtained.

### Moisture measurement

The method described in this subchapter refers to the datasets named “*20220428-20220503_VWC_processed.csv*”^18^ and “20240502-20240512_VWC_processed.csv”^22^.

**Fig. 9:**
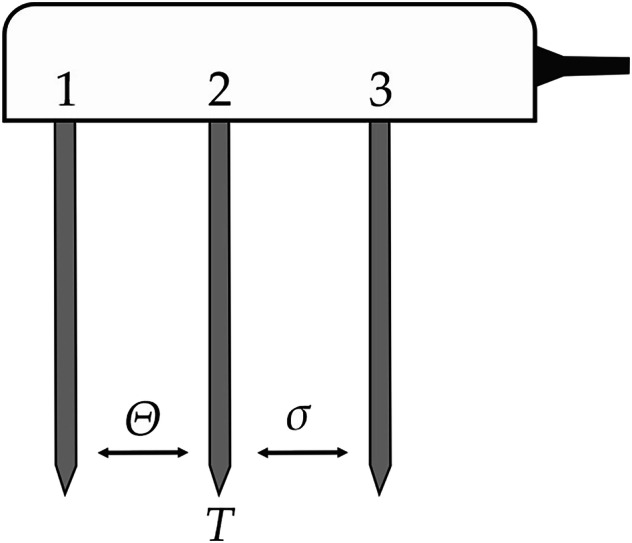
Schematic structure of the soil moisture sensor, including stainless steel probes for the measurement of the volumetric water content *θ*, the temperature *T*, and the electrical conductivity *σ*.

Figure 9 shows the schematic structure of the installed soil moisture sensors.

The volumetric water content is indirectly measured by measuring the dielectric permittivity of the soil *ε*_r_ between needle 1 and needle 2 (Fig. 9). The evaluation is based on the significantly different permittivity values of the soil components. The permittivity of minerals usually ranges only from 2 to 5 F/m^27^, whereas water and air exhibit distinctively different permittivity vakzes of 81 F/m and 1 F/m, respectively. Thus, the permittivity of the soil can be a direct indicator of the soil moisture content. The dielectric permittivity is measured by frequency domain reflectometry at a dielectric measurement frequency of 70 MHz. The raw output of the measurement (RAW) is further processed by applying the manufacturer calibration for mineral soils to calculate the volumetric water content *θ* in m^3^/m^3^:

10 $${\rm{\theta }}=3.879\cdot {10}^{-4}\cdot {RAW}-0.6956$$

The calibration is valid for a volumetric water content from 0.00 to 0.70 m^3^/m^3^. The influenced subsurface volume is stated to be 1.01 l. The resolution of the measurement is given with 0.001 m^3^/m^3^. The accuracy of applying the generic calibration is ±0.03 m^3^/m^3^ (for typical mineral soils with EC < 8 dS/m). The accuracy of the apparent dielectric permittivity *ε*_a_ is ± 1 in the measuring range between 1 and 40, and 15% of the measured value in the measuring range between 40 and 80.

The temperature is measured with an inbuilt thermistor in the centre needle. The resolution of the measurement is given with 0.1 °C. The accuracy is ±0.5 °C from −40 to 0 °C, and ±0.3 °C from 0 to +60 °C.

Bulk electrical conductivity is identified by measuring the electrical resistance between needle 2 and needle 3 due to an alternating electrical current. The determined electrical conductivities *EC*_*T*_ at a specific temperature *T* are corrected to the electrical conductivity at 25 °C, *EC*_25_:

11 $${{EC}}_{25}=\frac{{{EC}}_{T}}{1+0.017\cdot \left(T-25\right)}$$

The electrical conductivity measurements are calibrated by the manufacturer. The resolution of the measurement is given with 0.001 mS/cm. The accuracy is ± (5% + 0.01 mS/cm) from 0 to 10 mS/cm.

Before installation of the soil moisture sensors, they were tested in the laboratory for their accuracy regarding volumetric water content and temperature. The volumetric water content is determined by applying the measurement to quartz sand at three different soil moisture contents (dry, soil-moist, saturated). For dry soil, the deviation is 0.036 m³/m³ and thereby only slightly above the measurement accuracy of 0.03 m³/m³. For moist soil, the deviation is below the measurement uncertainty. For saturated soil, the deviation is 0.047 m³/m³, which can be mainly related to uncertainties in achieving a total saturation of the tested soil. The temperature measurement is tested in a calibration bath. The deviations between the measurements are below 0.5 °C and thereby below the given accuracy of the temperature probe. Soil-specific calibration is not conducted as the accuracies of the manufacturer calibration are sufficient for the sought application and interpretation of the measurement data.

**Fig. 10:**
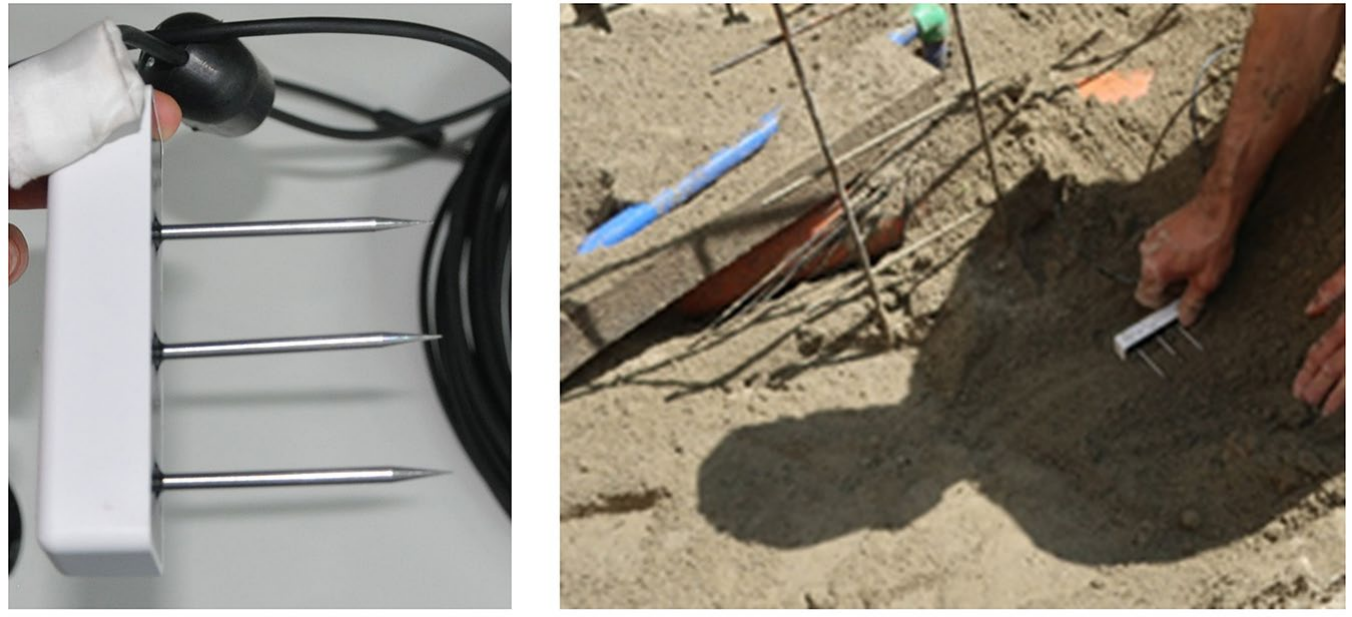
Installation of the soil moisture sensors at a close distance from the planar trench collector.

Five soil moisture sensors (TEROS 12, METER Group, Inc.) are installed in L-shape close to the planar trench collectors (see sketch Fig. 6 or further detailed information in the repository^18^). The installation process is shown in Fig. 10. The sensors are installed horizontally to ensure measurement of the entire precipitation water. Care is taken to ensure a good contact between the soil and the sensors. The sensors measure the volumetric water content but also soil temperature and bulk electric conductivity. Data acquisition is conducted with a designated logging device (Em50, METER Group, Inc.).

## Data Records

The datasets are available on Zenodo: https://zenodo.org/records/14069578^18^, https://zenodo.org/records/15369421^20^, https://zenodo.org/records/15369554^21^, https://zenodo.org/records/15425180^22^.

### PT 100 sensors

The temperature measurements of the PT 100 sensors can be found in the files named: 20220428-20220503_PT100_calibrated.csv, 20230228-20230306_PT100_calibrated.csv, 230406-230413_PT100_calibrated.csv and  20240502-20240512_PT100_calibrated.csv and are stored in the zenodo repositories^18,20–22^. The files contains the data of the period contained in its name, starting at 00:00:00 in hh:mm:ss of the first day until 23:59:00 of the last day of the period. The PT 100 measurements are continuously logged in one-minute intervals, so that there are measurements available for each day as long as there are no current faults. The separator between the measured values is comma, and the decimal separator of the value itself is a dot. For consistency reasons, the sensors installed at August 6^th^ 2024 are already labelled as described in the chapter Temperature measurement even before their installation date. The time stamp corresponds to the format: YYYY-MM-DD hh:mm:ss. The header name corresponds to the measurement points in the drawings in the repository^18^. The labelling system of the PT 100 sensors buried in the subsurface follows the structure shown in Table 2, whereas the sensors mounted in the pipes are defined by Table 3. The first three characters always define the system type, the 4^th^ character identifies the number of the system. In this case, it is the ground heat collector number 1 (GHC1). Then a separator is followed by a character for the relevant measurement type, e.g. *T* for temperature. For the sensors buried in the subsurface the coordinates of their location follow. The origin of the coordinate system is located at the border of the inlet shaft and is depicted in the drawings in the repository^18^. X represents the direction along the length of the trench collector. Y is the direction perpendicular to the length of the collector. Z is the depth but notated without the minus sign in the plant identification code. The first direction separated by an underscore is the depth below the ground surface in cm. Again, separated by an underscore, the lateral arrangement next to the collector follows (L for left side and R for right side of the collector in the direction of flow, i.e. R if y is positive and L if y is negative), combined with the absolute value of the normal distance from the axis to the location of the sensor in cm. The last specification, which is also preceded by a separator, corresponds to the distance in cm from the origin in x-direction. For all distances the coordinate system depicted in the drawings applies.

**Table 2:** Labelling system for the temperature sensors installed in the subsurface.

Geothermal Facility			Number	Separator	Measurement	Separator	Depth below top edge of terrain in cm			Separator	Collector side (left or right)	Numerical value normal distance to facility in cm			Separator	Distance from collector inlet in flow direction in cm			
1	2	3	4	5	6	7	8	9	10	11	12	13	14	15	16	17	18	19	
G	H	C																	Ground Heat Collector
			n	—															
					T														Temperature
					M														Moisture
						—													
							9	9	9										Unit cm
										—									
											L								L for left, R for right in the direction of flow
												9	9	9					Unit cm
															—				
																9	9	9	Unit cm

**Table 3:** Labelling system for the temperature sensors installed in the pipes.

Geothermal Facility			Number	Separator	Measurement	Separator	Position (inlet or outlet)			Separator	Reference component for the position				Separator	Additional information			
1	2	3	4	5	6	7	8	9	10	11	12	13	14	15	16	17	18	19	
G	H	C																	Ground Heat Collector
			n	—															
					T														Temperature
						—													
							I	N	L										Inlet
							O	U	T										Outlet
							S	P	R										Spare
										—									
											H	C	A	G					Heating-cooling aggregate
											C	O	L	L					Collector
											P	I	P	E					Connecting pipe
															—				
																I	N	S	Insulated
																N	I	N	Non-insulated

The labelling system of the screw-in sensors in the pipes is shown in Table 3. Here, the first seven characters are the same as those for the subsurface sensors. From the 8^th^ to the 10^th^ character, the position relative to a component is described, e.g. inlet and outlet. Separated by a delimiter, the 12^th^ to the 15^th^ characters indicate the corresponding component (e.g. collector, connecting pipe, heating-cooling aggregate etc.). In the case that certain specifications for sensors do not exist, these positions are filled with X. For smaller numbers than the designated character amount, the preceding positions are filled with 0.

Labels starting with the header names: GHC1_T_SPR_ are used for sensors which are connected but not mounted (SPR stands for: spare). Except for the sensor GHC1_T_SPR_0010_XXX, all of the unmounted sensors are located in the inlet shaft and are considered to be a rough reference for the outside temperature since they measure the air temperature in the inlet shaft. The sensor GHC1_T_SPR_0010_XXX is located in the laboratory near the heating-cooling unit to monitor the air temperature in the lab.

An excerpt of the dataset is shown in Table 4. The sensors were calibrated before installation, including the entire measuring section. More information on the calibration method can be found in subsection 2.2.1. The published data of the PT 100 sensors is calibrated.

**Table 4:** Extract from the measurement data file of the PT 100 sensors.

date_time	GHC1_T_078_R000_094	GHC1_T_078_R000_788	GHC1_T_078_R000_084
2022-04-28 00:00:00	9.601669066	9.800738852	9.555707613
2022-04-28 00:01:00	9.601669066	9.800738852	9.555707613

Further data series are planned for publication.

### Fibre optic

Initially, each individual measurement in the selected sampling interval is stored in a multiple data file in ASCII (American Standard Code for Information Interchange) format with the extension *tra*. For further data processing, the file is converted to a csv-file. In the example file, during a TRT, the temperature was recorded every two minutes with a sampling interval and a spatial resolution of 0.5 m. Decimals are separated by commas in the data file. An example of the data structure is shown in Table 5.

**Table 5:** Example of the data file of the fibre optic temperature measurements.

Measurement number	Date time	0*	0,5*	…*
1	28.04.2022 00:01:00	24.6543522	24.6656742	…
2	28.04.2022 00:03:00	24.490778	24.6440373	…

*Fibre length in meter.

The first column is the number of the measurement. The second column contains the date and time of the measurement in the format [DD.MM.YYYY hh:mm:ss]. From the third column onwards, the temperature data is displayed at intervals of 0.5 m.

### Moisture

The soil moisture data can be found in the files named: “20220428-20220503_VWC_processed.csv”^18^ and “20240502-20240512_VWC_processed.csv”^22^. The files contain the data logged every 10 minutes starting at 00:00:00 in hh:mm:ss of the first day until 23:59:00 of the last day of the period. The values are the average of data measured every minute across the measurement interval of 10 minutes. The decimal separator is a dot. Measurements are separated with a semicolon. The file is structured as follows: The first column includes the timestamp, containing the date and the time in the format YYYY-MM-DD hh:mm:ss. Each of the five soil moisture sensors (GHC1_M/T/C) is represented by three columns, consisting of the volumetric water content *θ* in m³/m³, the temperature *T* in °C and the bulk electrical conductivity *σ* in mS/cm.

## Technical Validation

**Fig. 11:**
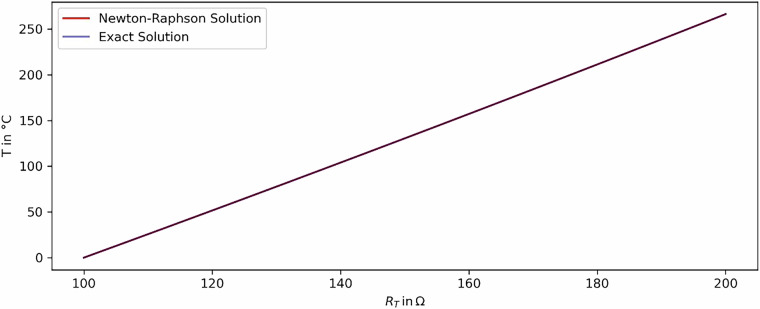
Comparison of the Newton-Raphson and the exact solution.

**Fig. 12:**
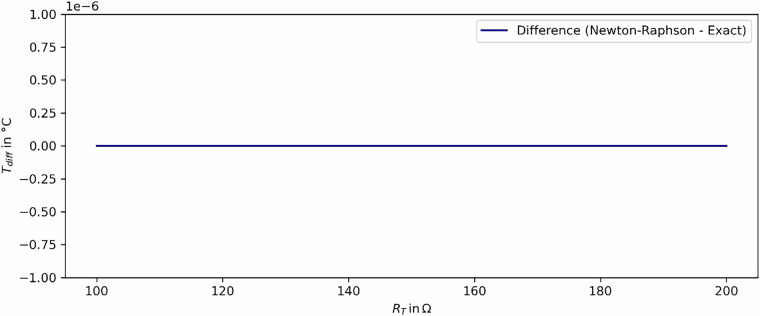
Deviation between the Newton-Raphson and the exact solution.

The numerical Newton-Raphson method which is used for the conversion of the PT 100 measurements into the temperatures is validated. A comparison of results between the numerical and the exact solutions was performed for temperatures above 0 °C, showing a deviation of less than $1\cdot {10}^{-6}$. Figure 11 shows the results for positive values of both the exact solution and the Newton-Raphson method. The deviation between both solutions is negligible and visualised in Fig. 12. The Python-code for the validation is provided in a GitHub repository.

## Supplementary information

Documentation of the calibrated PT100 sensors

## Data Availability

The four datasets are available on Zenodo: https://zenodo.org/records/1406957818, https://zenodo.org/records/1536942120, https://zenodo.org/records/1536955421, https://zenodo.org/records/1542518022. They all contain the measurement data of the PT 100 sensors, and, where available, the described moisture sensor data and fibre optic measurement data.

## Code availability

The custom code used for the validation of Newton-Raphson method is provided in the following GitHub repository: https://github.com/AdindaVandeVen/validation_Newton-Raphson_method.
