# Supplementary material for: Thermal reaction of the subsurface on the operation of a geothermal planar trench collector
Source: Sci Data. 2025 Oct 20;12:1661. doi: 10.1038/s41597-025-06072-8 (PMC12537951; doi:10.1038/s41597-025-06072-8)
Supplement: Supplementary file 1 — Documentation of the calibrated PT100 sensors [file 41597_2025_6072_MOESM1_ESM.pdf]

Quality Enhancement of Shallow Geothermal Systems

# - QEWSplus -

Documentation of the calibrated PT 100 sensors

Attachment to the Data Descriptor:

Thermal reaction of the subsurface on the operation of a geothermal planar trench collector

Last updated on

August 22nd 2025

Supported by:

Federal Ministry for Economic Affairs and Climate Action

on the basis of a decision by the German Bundestag

Grant number: 03EE4020A-H

Project duration: January 1st 2021 – December 31st 2024

List of figures

Figure 1: necessary correction of GHC1_T_078_R0000_094 defined by calibration of the temperature sensor 5
Figure 2: necessary correction of GHC1_T_078_R000_788 defined by calibration of the temperature sensor 5
Figure 3: necessary correction of GHC1_T_078_R000_084 defined by calibration of the temperature sensor 6
Figure 4: necessary correction of GHC1_T_078_R000_798 defined by calibration of the temperature sensor 6
Figure 5: necessary correction of GHC1_T_070_L012_760 defined by calibration of the temperature sensor 7
Figure 6: necessary correction of GHC1_T_070_L030_595 defined by calibration of the temperature sensor 7
Figure 7: necessary correction of GHC1_T_070_L020_415 defined by calibration of the temperature sensor 8
Figure 8: necessary correction of GHC1_T_070_L028_250 defined by calibration of the temperature sensor 8
Figure 9: necessary correction of GHC1_T_070_L040_070 defined by calibration of the temperature sensor 9
Figure 10: necessary correction of GHC1_T_070_R007_760 defined by calibration of the temperature sensor 9
Figure 11: necessary correction of GHC1_T_070_R010_595 defined by calibration of the temperature sensor 10
Figure 12: necessary correction of GHC1_T_070_R005_415 defined by calibration of the temperature sensor 10
Figure 13: necessary correction of GHC1_T_070_R017_250 defined by calibration of the temperature sensor 11
Figure 14: necessary correction of GHC1_T_070_R032_070 defined by calibration of the temperature sensor 11
Figure 15: necessary correction of GHC1_T_070_L052_760 defined by calibration of the temperature sensor 12
Figure 16: necessary correction of GHC1_T_130_L052_760 defined by calibration of the temperature sensor 12
Figure 17: necessary correction of GHC1_T_184_L052_760 defined by calibration of the temperature sensor 13
Figure 18: necessary correction of GHC1_T_070_L052_595 defined by calibration of the temperature sensor 13
Figure 19: necessary correction of GHC1_T_130_L052_595 defined by calibration of the temperature sensor 14
Figure 20: necessary correction of GHC1_T_184_L052_595 defined by calibration of the temperature sensor 14
Figure 21: necessary correction of GHC1_T_070_L052_415 defined by calibration of the temperature sensor 15
Figure 22: necessary correction of GHC1_T_130_L052_415 defined by calibration of the temperature sensor 15
Figure 23: necessary correction of GHC1_T_184_L052_415 defined by calibration of the temperature sensor 16
Figure 24: necessary correction of GHC1_T_184_L052_250 defined by calibration of the temperature sensor 16
Figure 25: necessary correction of GHC1_T_130_R000_094 defined by calibration of the temperature sensor 17
Figure 26: necessary correction of GHC1_T_130_R000_788 defined by calibration of the temperature sensor 17
Figure 27: necessary correction of GHC1_T_130_R000_084 defined by calibration of the temperature sensor 18
Figure 28: necessary correction of GHC1_T_130_R000_798 defined by calibration of the temperature sensor 18
Figure 29: necessary correction of GHC1_T_130_L040_070 defined by calibration of the temperature sensor 19
Figure 30: necessary correction of GHC1_T_130_L028_250 defined by calibration of the temperature sensor 19
Figure 31: necessary correction of GHC1_T_130_L020_415 defined by calibration of the temperature sensor 20
Figure 32: necessary correction of GHC1_T_130_L030_595 defined by calibration of the temperature sensor 20
Figure 33: necessary correction of GHC1_T_130_L012_760 defined by calibration of the temperature sensor 21
Figure 34: necessary correction of GHC1_T_130_R007_760 defined by calibration of the temperature sensor 21
Figure 35: necessary correction of GHC1_T_130_R010_595 defined by calibration of the temperature sensor 22
Figure 36: necessary correction of GHC1_T_130_R005_415 defined by calibration of the temperature sensor 22
Figure 37: necessary correction of GHC1_T_130_R017_250 defined by calibration of the temperature sensor 23
Figure 38: necessary correction of GHC1_T_130_R032_070 defined by calibration of the temperature sensor 23

Figure 39: necessary correction of GHC1_T_013_R076_123 defined by calibration of the temperature sensor 24
Figure 40: necessary correction of GHC1_T_015_R076_123 defined by calibration of the temperature sensor 24
Figure 41: necessary correction of GHC1_T_010_R076_123 defined by calibration of the temperature sensor 25
Figure 42: necessary correction of GHC1_T_030_R076_123 defined by calibration of the temperature sensor 25
Figure 43: necessary correction of GHC1_T_045_R076_123 defined by calibration of the temperature sensor 26
Figure 44: necessary correction of GHC1_T_060_R076_123 defined by calibration of the temperature sensor 26
Figure 45: necessary correction of GHC1_T_070_R076_123 defined by calibration of the temperature sensor 27
Figure 46: necessary correction of GHC1_T_080_R076_123 defined by calibration of the temperature sensor 27
Figure 47: necessary correction of GHC1_T_090_R076_123 defined by calibration of the temperature sensor 28
Figure 48: necessary correction of GHC1_T_100_R076_123 defined by calibration of the temperature sensor 28
Figure 49: necessary correction of GHC1_T_182_R000_094 defined by calibration of the temperature sensor 29
Figure 50: necessary correction of GHC1_T_182_R000_788 defined by calibration of the temperature sensor 29
Figure 51: necessary correction of GHC1_T_182_R000_084 defined by calibration of the temperature sensor 30
Figure 52: necessary correction of GHC1_T_182_R000_798 defined by calibration of the temperature sensor 30
Figure 53: necessary correction of GHC1_T_070_L052_250 defined by calibration of the temperature sensor 31
Figure 54: necessary correction of GHC1_T_130_L052_250 defined by calibration of the temperature sensor 31
Figure 55: necessary correction of GHC1_T_070_L052_070 defined by calibration of the temperature sensor 32
Figure 56: necessary correction of GHC1_T_130_L052_070 defined by calibration of the temperature sensor 32
Figure 57: necessary correction of GHC1_T_184_L052_070 defined by calibration of the temperature sensor 33
Figure 58: necessary correction of GHC1_T_184_R007_760 defined by calibration of the temperature sensor 33
Figure 59: necessary correction of GHC1_T_184_R010_595 defined by calibration of the temperature sensor 34
Figure 60: necessary correction of GHC1_T_184_R005_415 defined by calibration of the temperature sensor 34
Figure 61: necessary correction of GHC1_T_184_R017_250 defined by calibration of the temperature sensor 35
Figure 62: necessary correction of GHC1_T_184_R032_070 defined by calibration of the temperature sensor 35
Figure 63: necessary correction of GHC1_T_184_L012_760 defined by calibration of the temperature sensor 36
Figure 64: necessary correction of GHC1_T_184_L030_595 defined by calibration of the temperature sensor 36
Figure 65: necessary correction of GHC1_T_184_L020_415 defined by calibration of the temperature sensor 37
Figure 66: necessary correction of GHC1_T_184_L028_250 defined by calibration of the temperature sensor 37
Figure 67: necessary correction of GHC1_T_184_L040_070 defined by calibration of the temperature sensor 38
Figure 68: necessary correction of GHC1_T_120_R076_123 defined by calibration of the temperature sensor 38
Figure 69: necessary correction of GHC1_T_140_R076_123 defined by calibration of the temperature sensor 39
Figure 70: necessary correction of GHC1_T_160_R076_123 defined by calibration of the temperature sensor 39
Figure 71: necessary correction of GHC1_T_180_R076_123 defined by calibration of the temperature sensor 40
Figure 72: necessary correction of GHC1_T_200_R076_123 defined by calibration of the temperature sensor 40
Figure 73: necessary correction of GHC1_T_182_R008_778 defined by calibration of the temperature sensor 41
Figure 74: necessary correction of GHC1_T_182_L005_525 defined by calibration of the temperature sensor 41
Figure 75: necessary correction of GHC1_T_182_L014_415 defined by calibration of the temperature sensor 42
Figure 76: necessary correction of GHC1_T_182_L018_250 defined by calibration of the temperature sensor 42
Figure 77: necessary correction of GHC1_T_182_R001_106 defined by calibration of the temperature sensor 43

Figure 78: necessary correction of GHC1_T_130_R008_778 defined by calibration of the temperature sensor 43
Figure 79: necessary correction of GHC1_T_130_L005_525 defined by calibration of the temperature sensor 44
Figure 80: necessary correction of GHC1_T_130_L014_415 defined by calibration of the temperature sensor 44
Figure 81: necessary correction of GHC1_T_130_L018_250 defined by calibration of the temperature sensor 45
Figure 82: necessary correction of GHC1_T_130_R001_106 defined by calibration of the temperature sensor 45
Figure 83: necessary correction of GHC1_T_078_R008_778 defined by calibration of the temperature sensor 46
Figure 84: necessary correction of GHC1_T_078_L005_595 defined by calibration of the temperature sensor 46
Figure 85: necessary correction of GHC1_T_078_L014_415 defined by calibration of the temperature sensor 47
Figure 86: necessary correction of GHC1_T_078_L018_250 defined by calibration of the temperature sensor 47
Figure 87: necessary correction of GHC1_T_078_R001_106 defined by calibration of the temperature sensor 48
Figure 88: necessary correction of GHC1_T_001_R004_072 defined by calibration of the temperature sensor 48
Figure 89: necessary correction of GHC1_T_SPR_0002_XXX defined by calibration of the temperature sensor 49
Figure 90: necessary correction of GHC1_T_SPR_0003_XXX defined by calibration of the temperature sensor 49
Figure 91: necessary correction of GHC1_T_SPR_0004_XXX defined by calibration of the temperature sensor 50
Figure 92: necessary correction of GHC1_T_001_L017_778 defined by calibration of the temperature sensor 50
Figure 93: necessary correction of GHC1_T_SPR_0006_XXX defined by calibration of the temperature sensor 51
Figure 94: necessary correction of GHC1_T_SPR_0007_XXX defined by calibration of the temperature sensor 51
Figure 95: necessary correction of GHC1_T_OUT_PIPE_INS defined by calibration of the temperature sensor 52
Figure 96: necessary correction of GHC1_T_OUT_PIPE_NIN defined by calibration of the temperature sensor 52
Figure 97: necessary correction of GHC1_T_SPR_0008_XXX defined by calibration of the temperature sensor 53
Figure 98: necessary correction of GHC1_T_INL_COLL_XXX defined by calibration of the temperature sensor 53
Figure 99: necessary correction of GHC1_T_OUT_COLL defined by calibration of the temperature sensor 54
Figure 100: necessary correction of GHC1_T_OXX_HCAG defined by calibration of the temperature sensors 54
Figure 101: necessary correction of GHC_1_T_IXX_HCAG defined by calibration of the temperature sensors 55

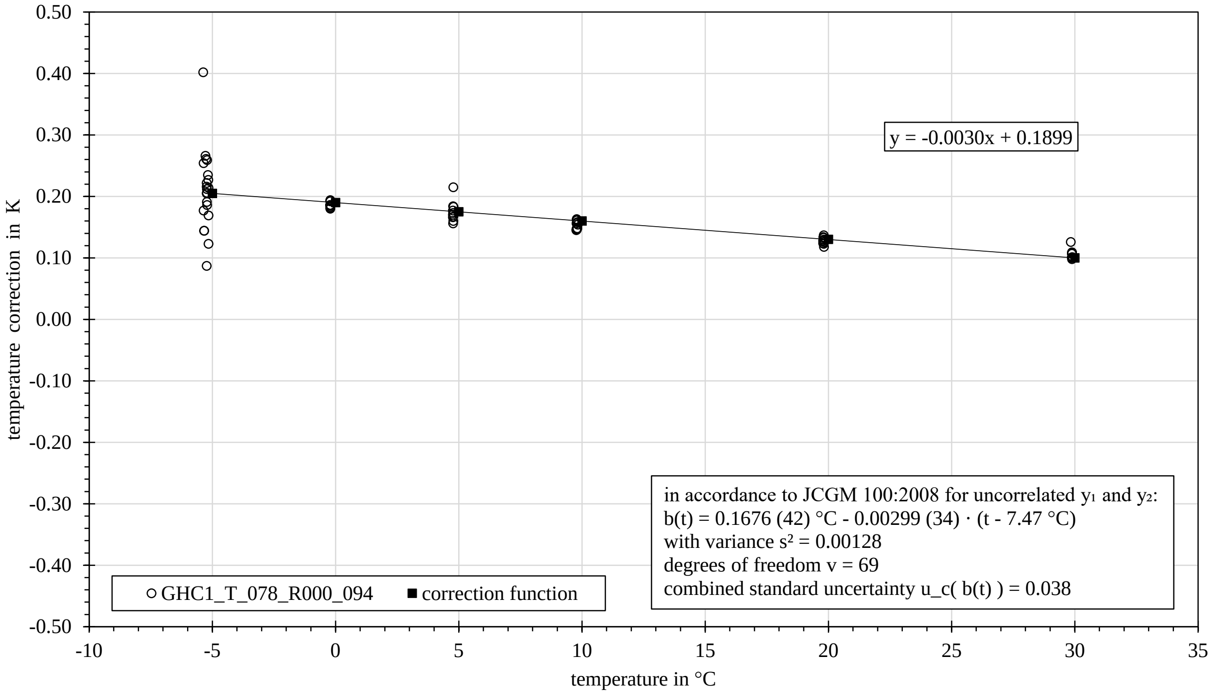

Figure 1: necessary correction of GHC1_T_078_R0000_094 defined by calibration of the temperature sensor

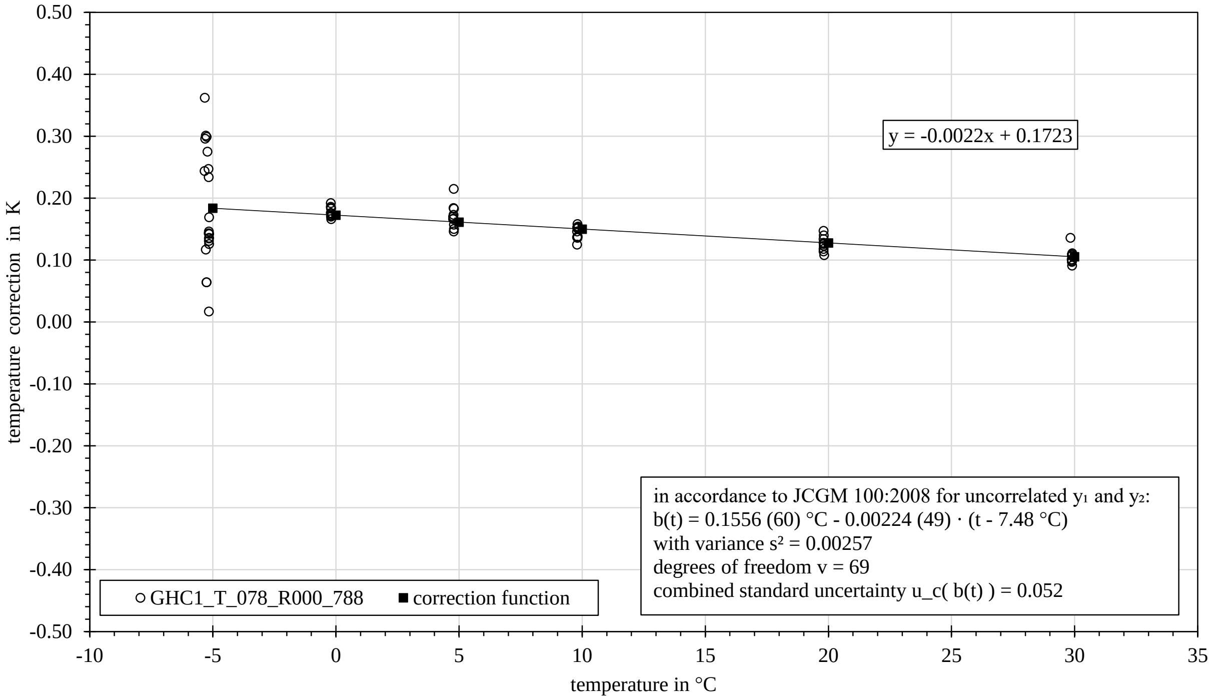

Figure 2: necessary correction of GHC1_T_078_R000_788 defined by calibration of the temperature sensor

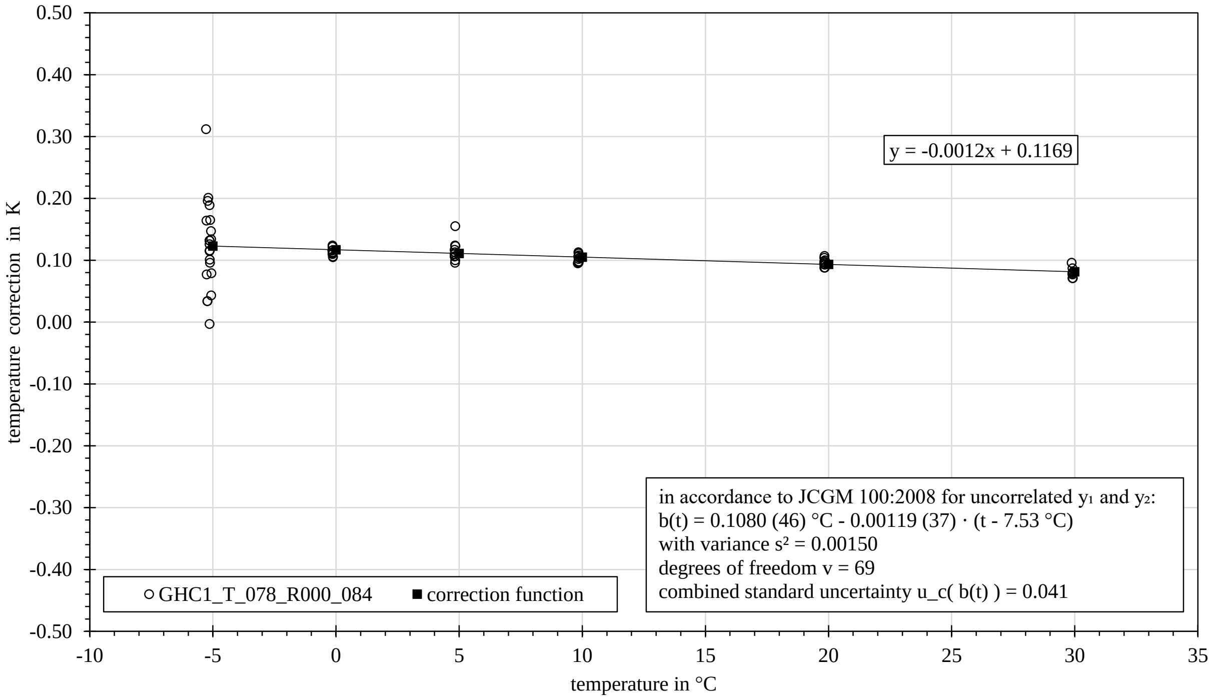

Figure 3: necessary correction of GHC1_T_078_R000_084 defined by calibration of the temperature sensor

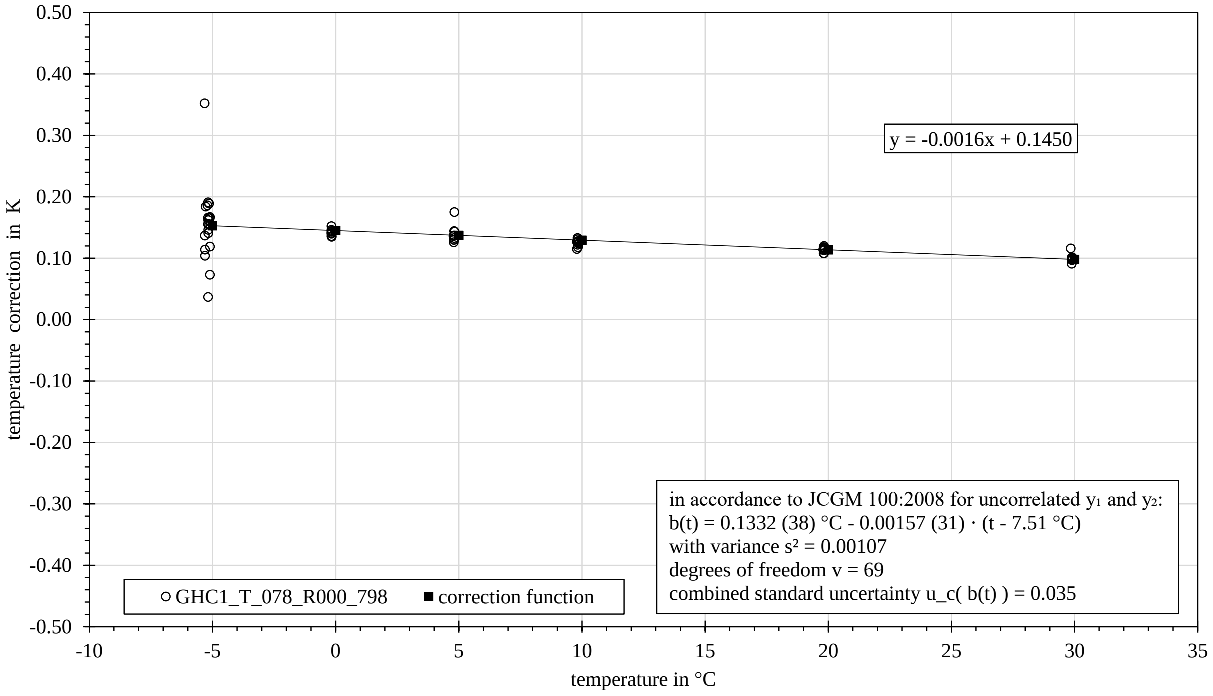

Figure 4: necessary correction of GHC1_T_078_R000_798 defined by calibration of the temperature sensor

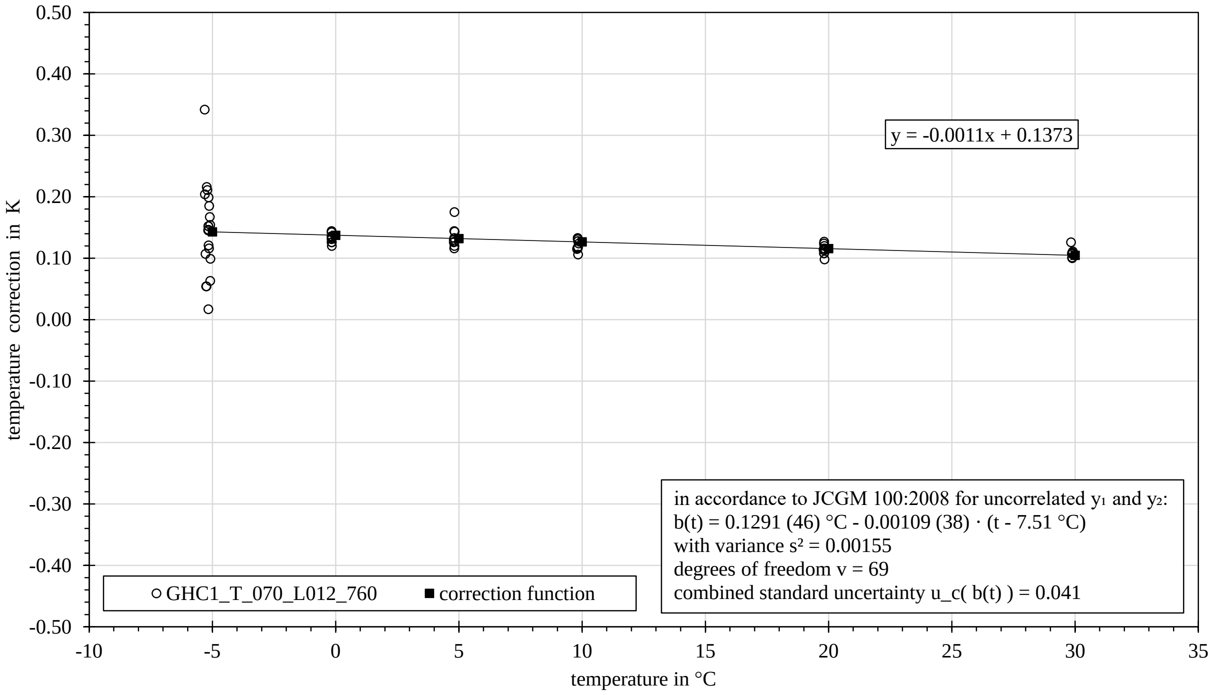

Figure 5: necessary correction of GHC1_T_070_L012_760 defined by calibration of the temperature sensor

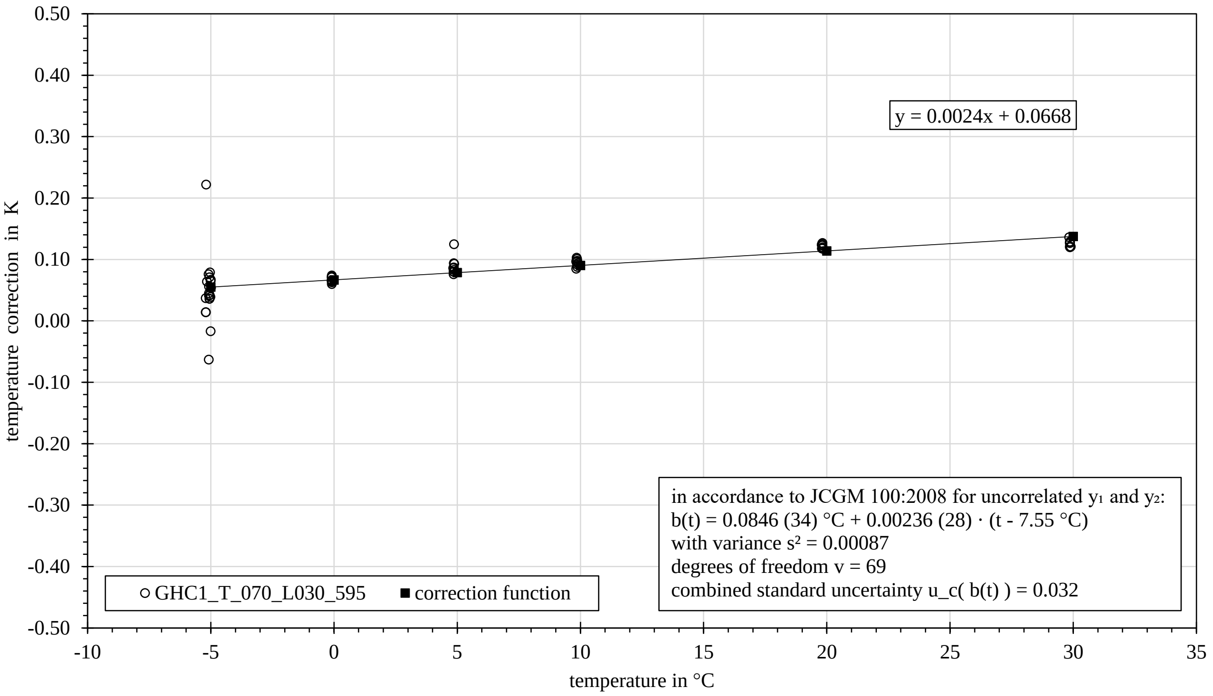

Figure 6: necessary correction of GHC1_T_070_L030_595 defined by calibration of the temperature sensor

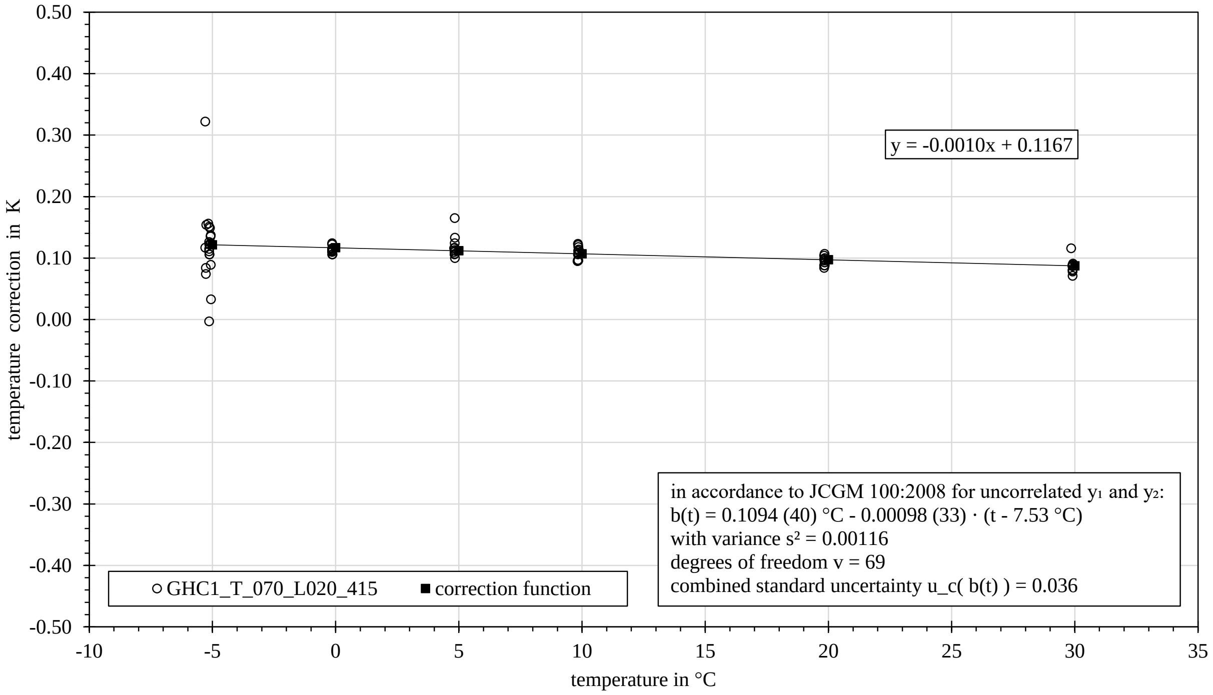

Figure 7: necessary correction of GHC1_T_070_L020_415 defined by calibration of the temperature sensor

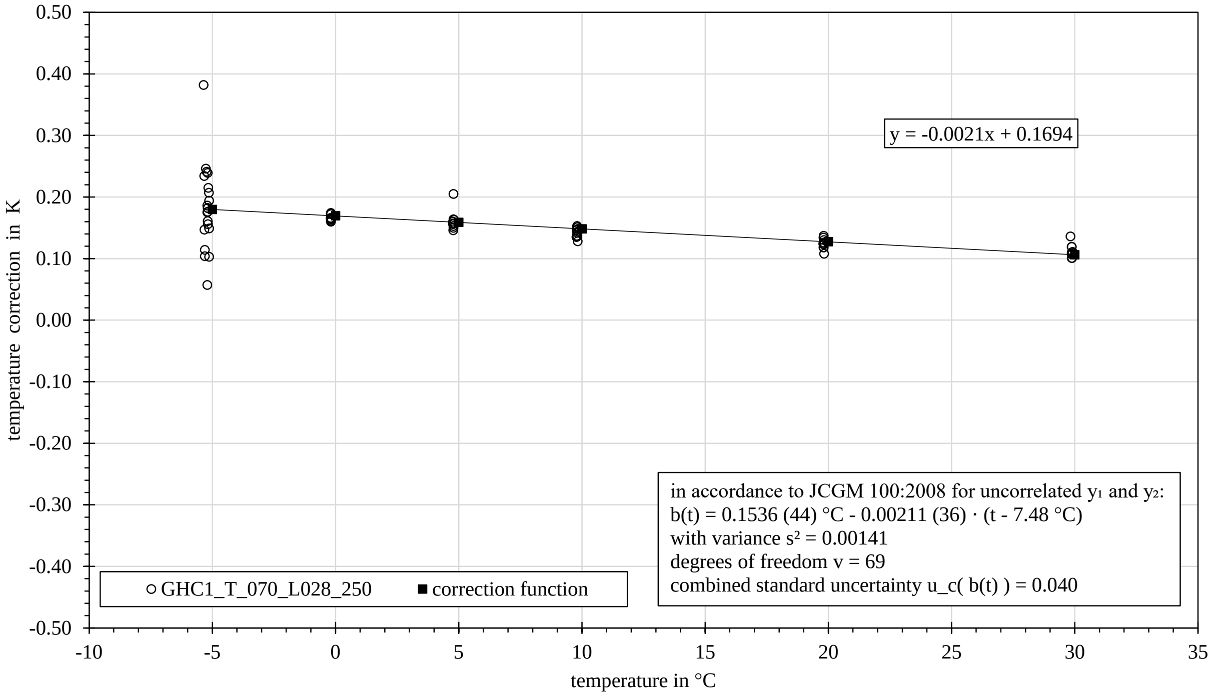

Figure 8: necessary correction of GHC1_T_070_L028_250 defined by calibration of the temperature sensor

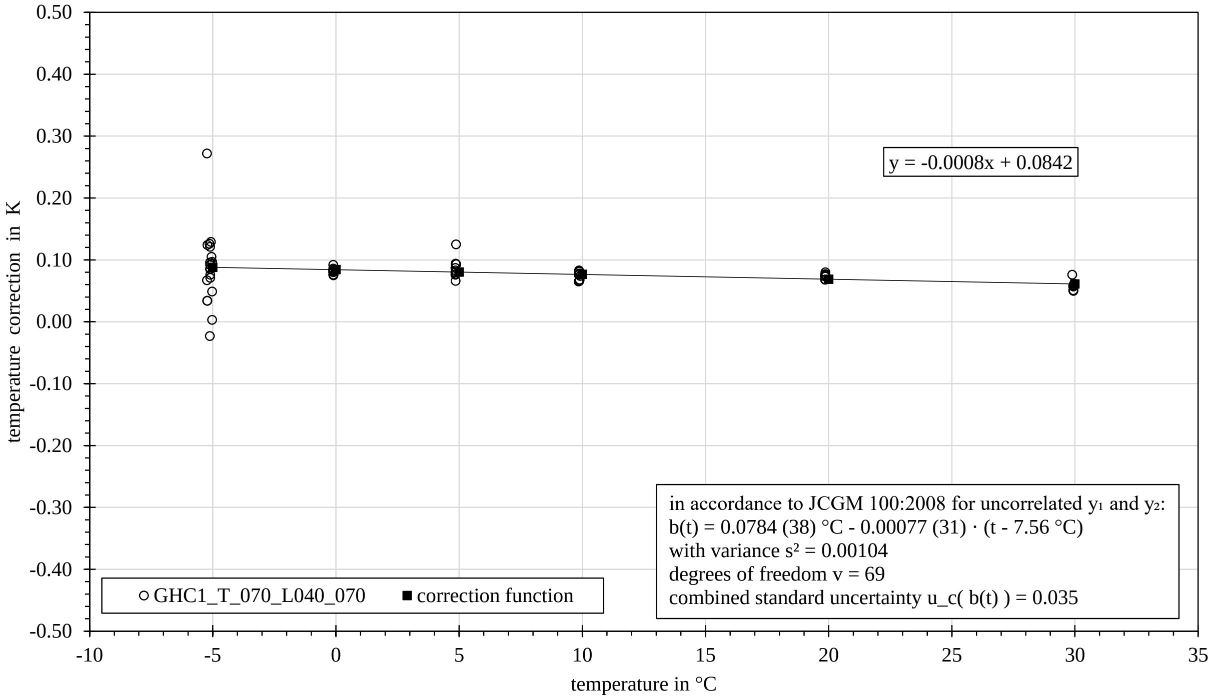

Figure 9: necessary correction of GHC1_T_070_L040_070 defined by calibration of the temperature sensor

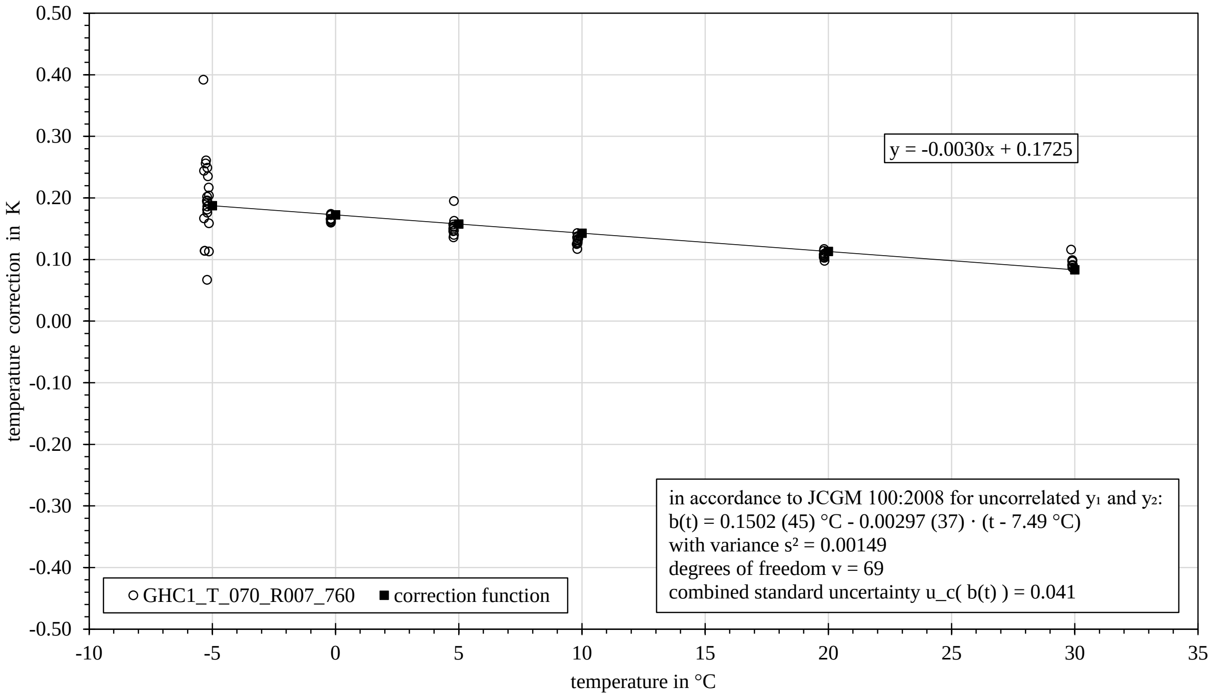

Figure 10: necessary correction of GHC1_T_070_R007_760 defined by calibration of the temperature sensor

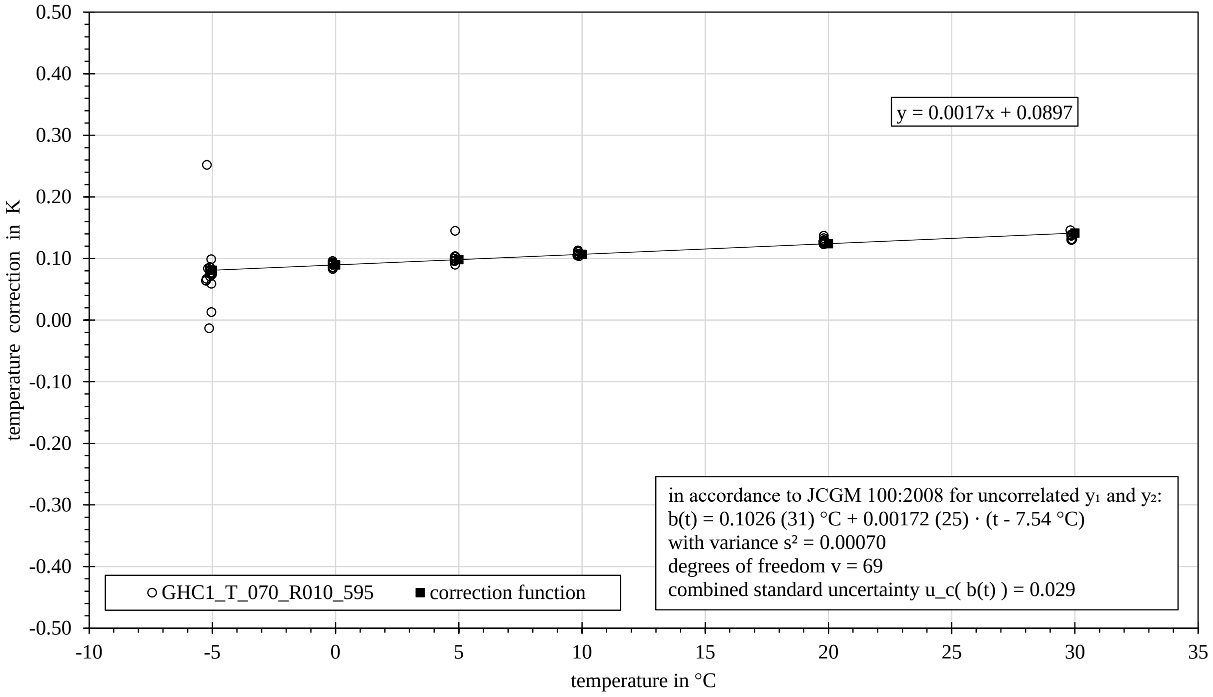

Figure 11: necessary correction of GHC1_T_070_R010_595 defined by calibration of the temperature sensor

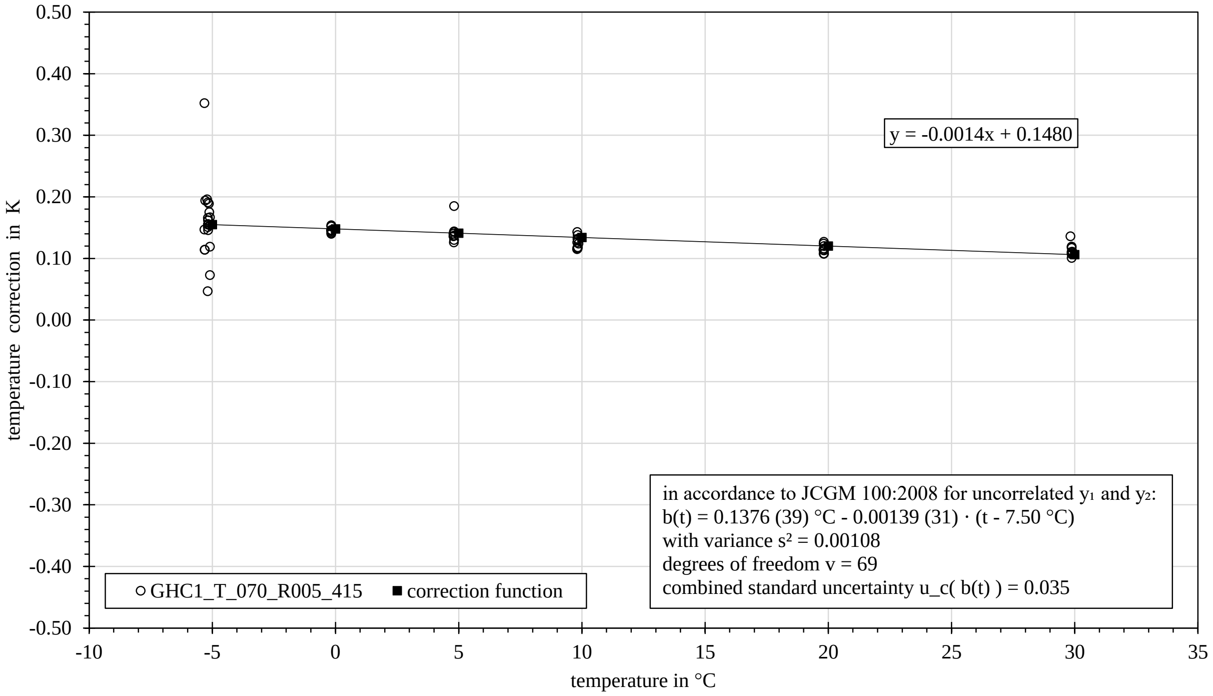

Figure 12: necessary correction of GHC1_T_070_R005_415 defined by calibration of the temperature sensor

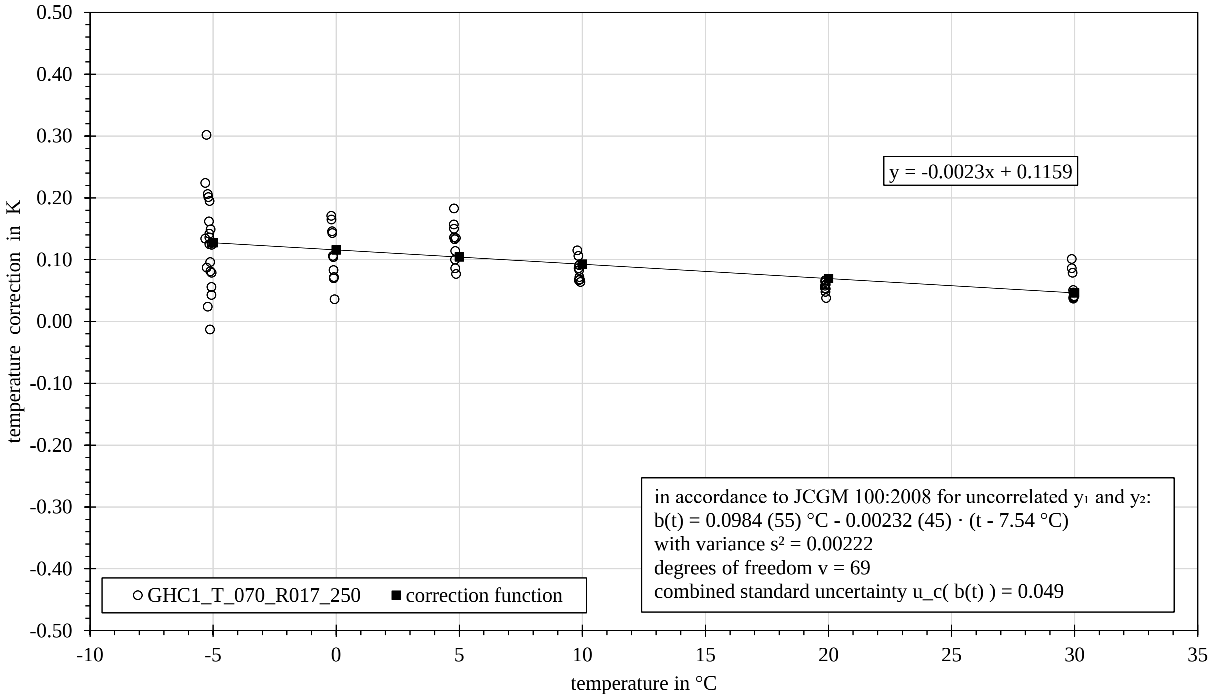

Figure 13: necessary correction of GHC1_T_070_R017_250 defined by calibration of the temperature sensor

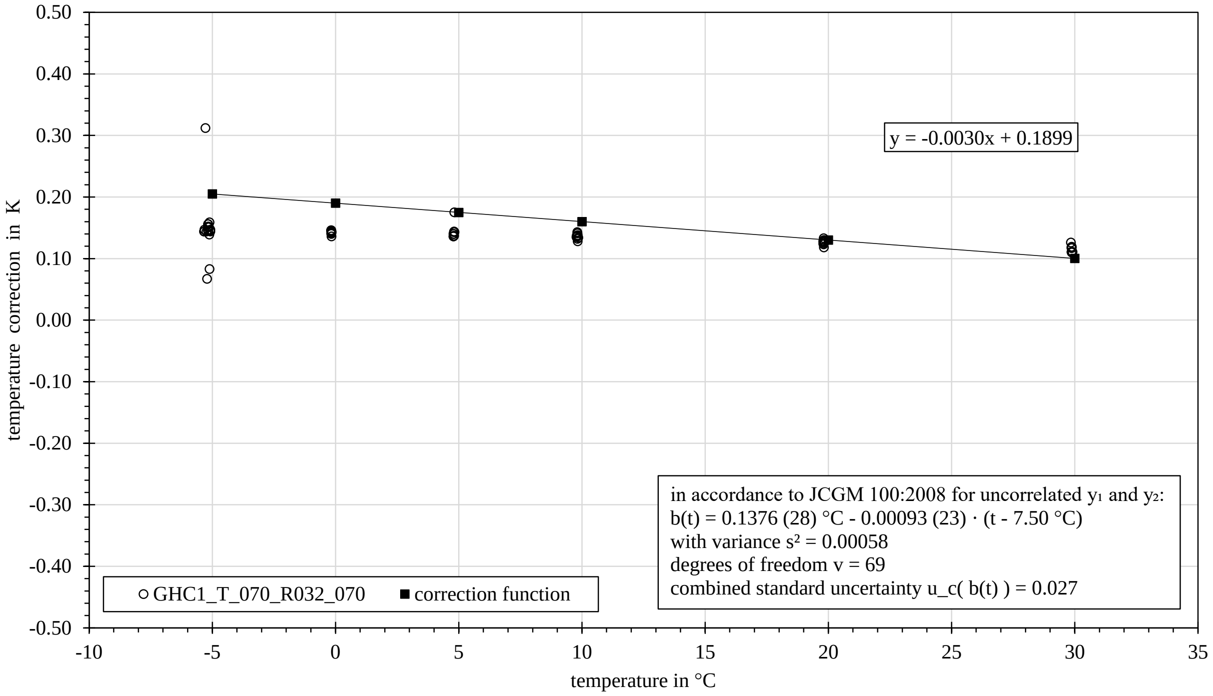

Figure 14: necessary correction of GHC1_T_070_R032_070 defined by calibration of the temperature sensor

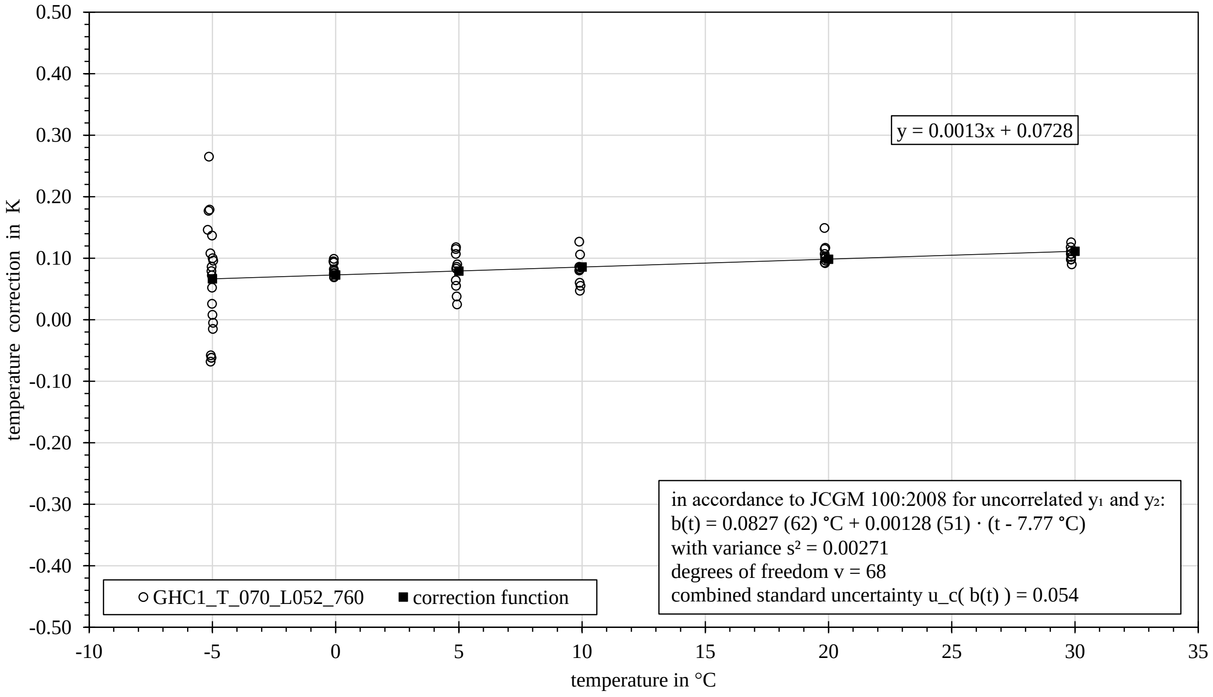

Figure 15: necessary correction of GHC1_T_070_L052_760 defined by calibration of the temperature sensor

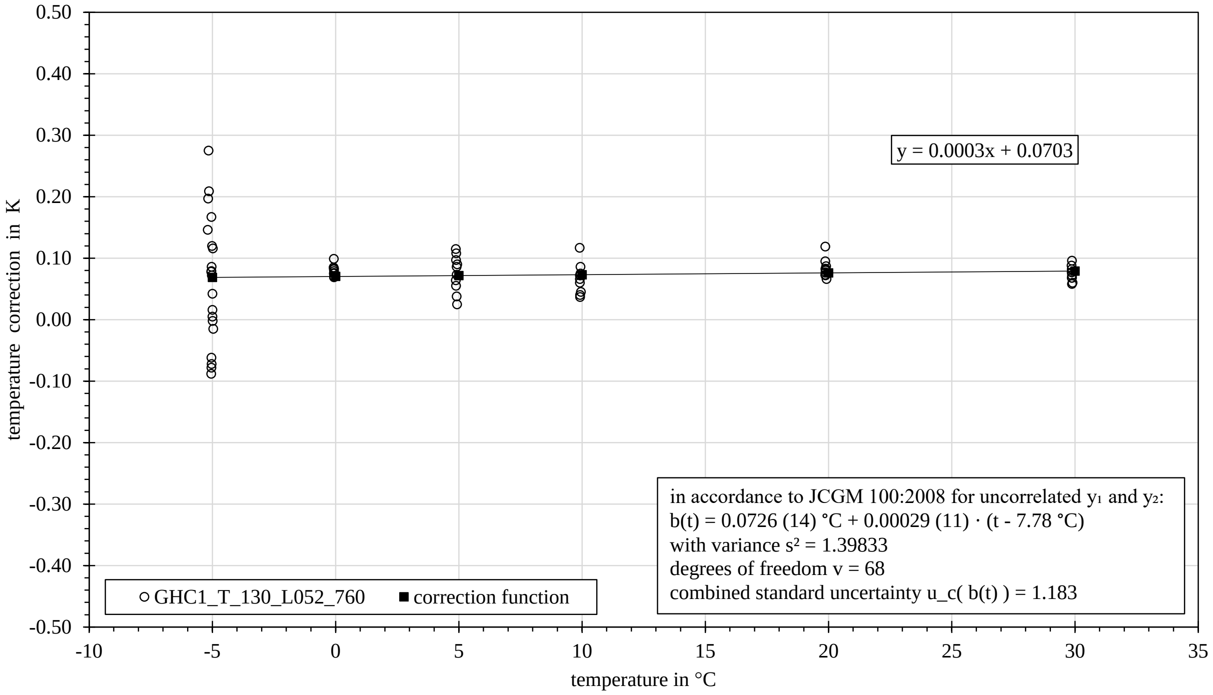

Figure 16: necessary correction of GHC1_T_130_L052_760 defined by calibration of the temperature sensor

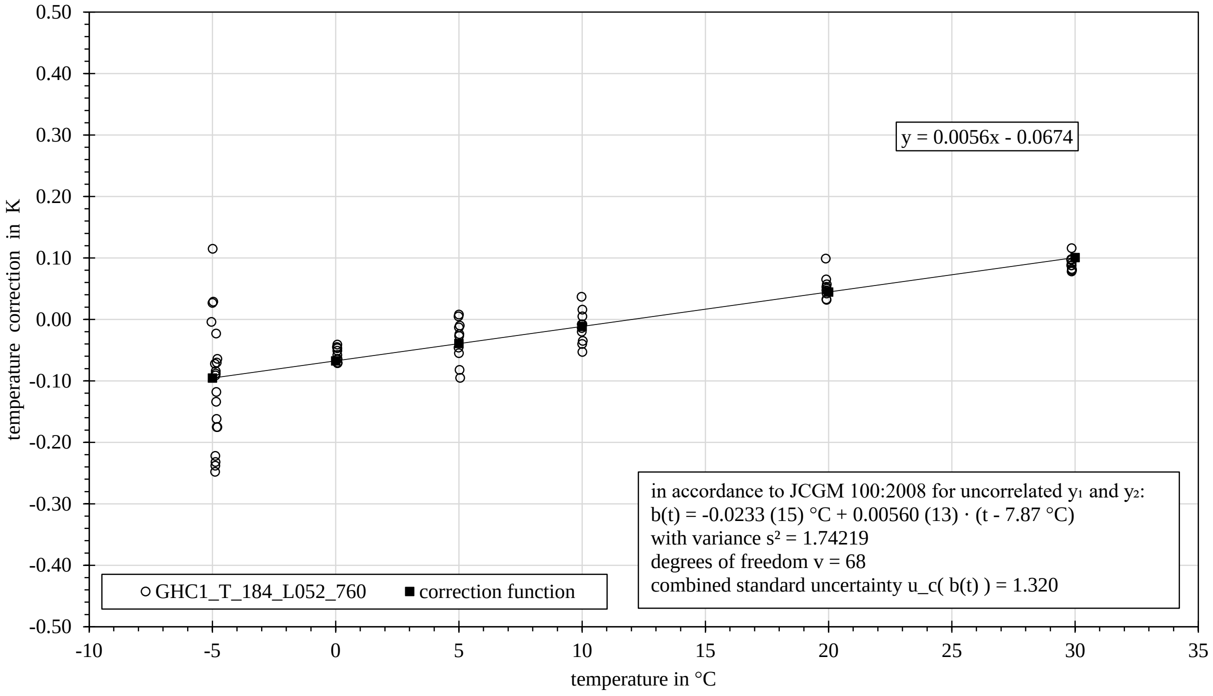

Figure 17: necessary correction of GHC1_T_184_L052_760 defined by calibration of the temperature sensor

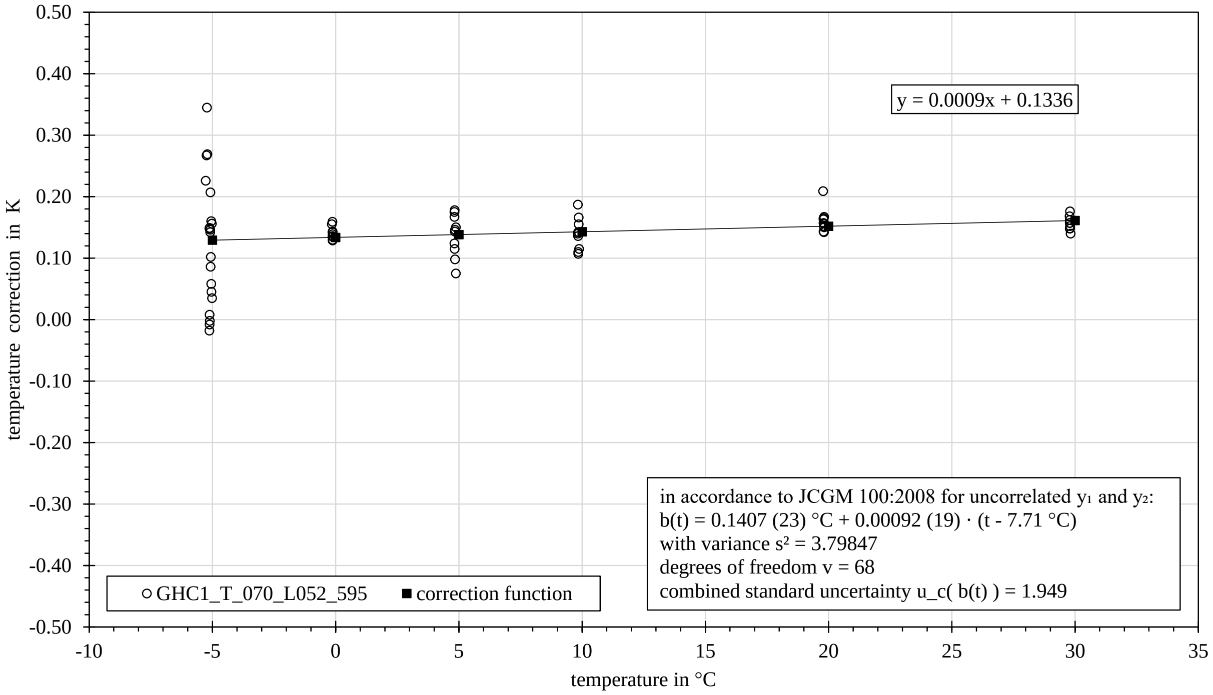

Figure 18: necessary correction of GHC1_T_070_L052_595 defined by calibration of the temperature sensor

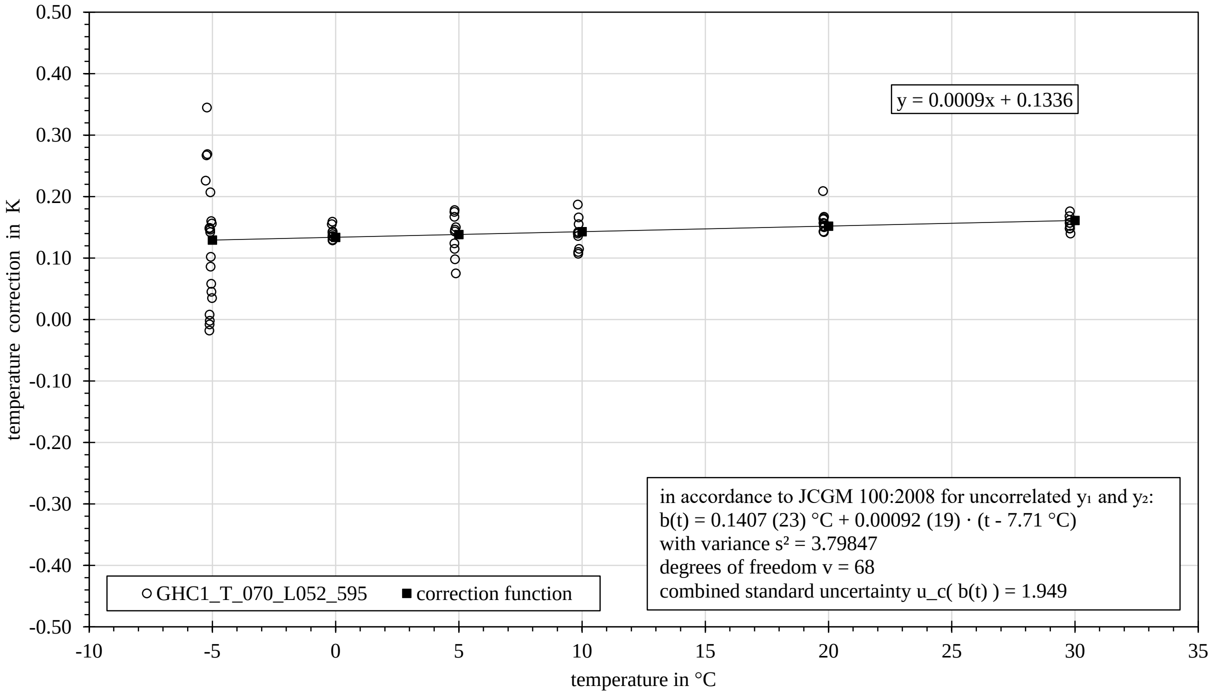

Figure 19: necessary correction of GHC1_T_130_L052_595 defined by calibration of the temperature sensor

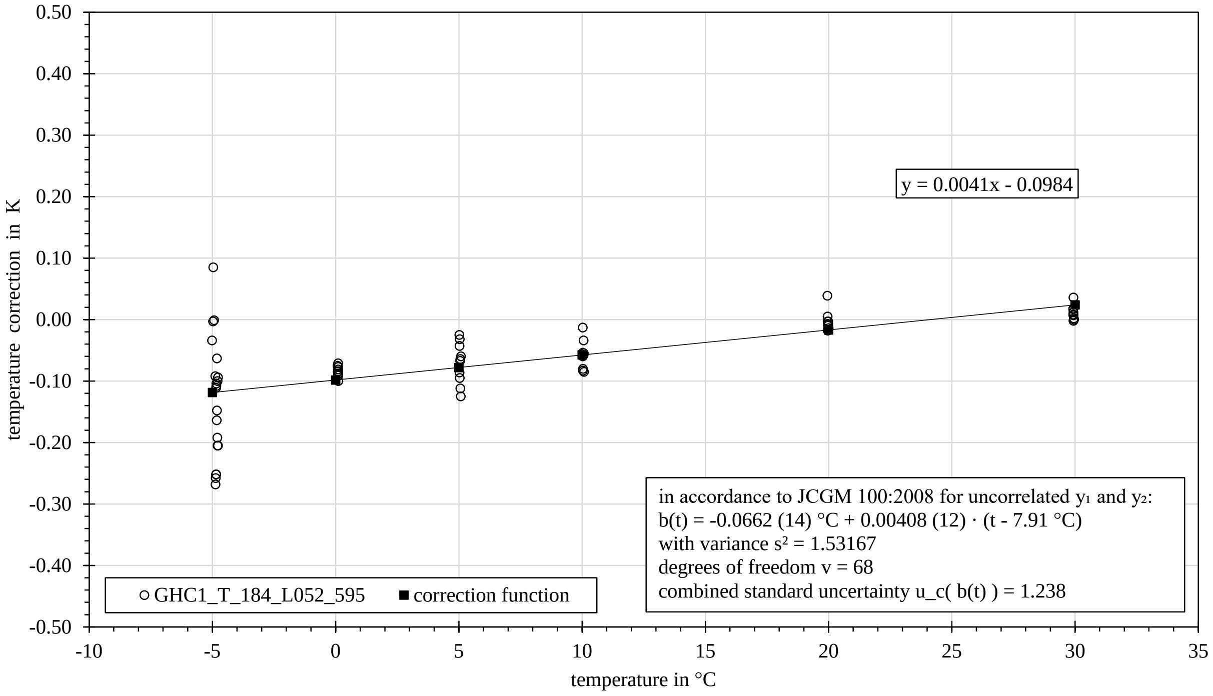

Figure 20: necessary correction of GHC1_T_184_L052_595 defined by calibration of the temperature sensor

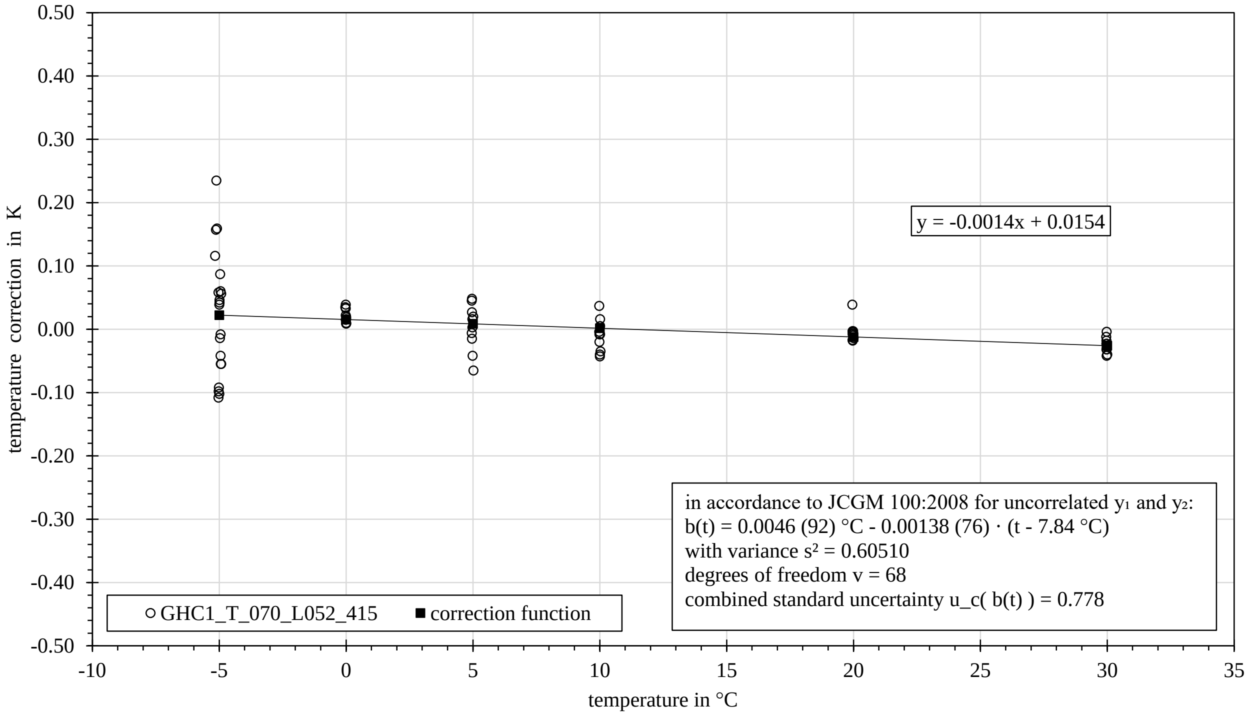

Figure 21: necessary correction of GHC1_T_070_L052_415 defined by calibration of the temperature sensor

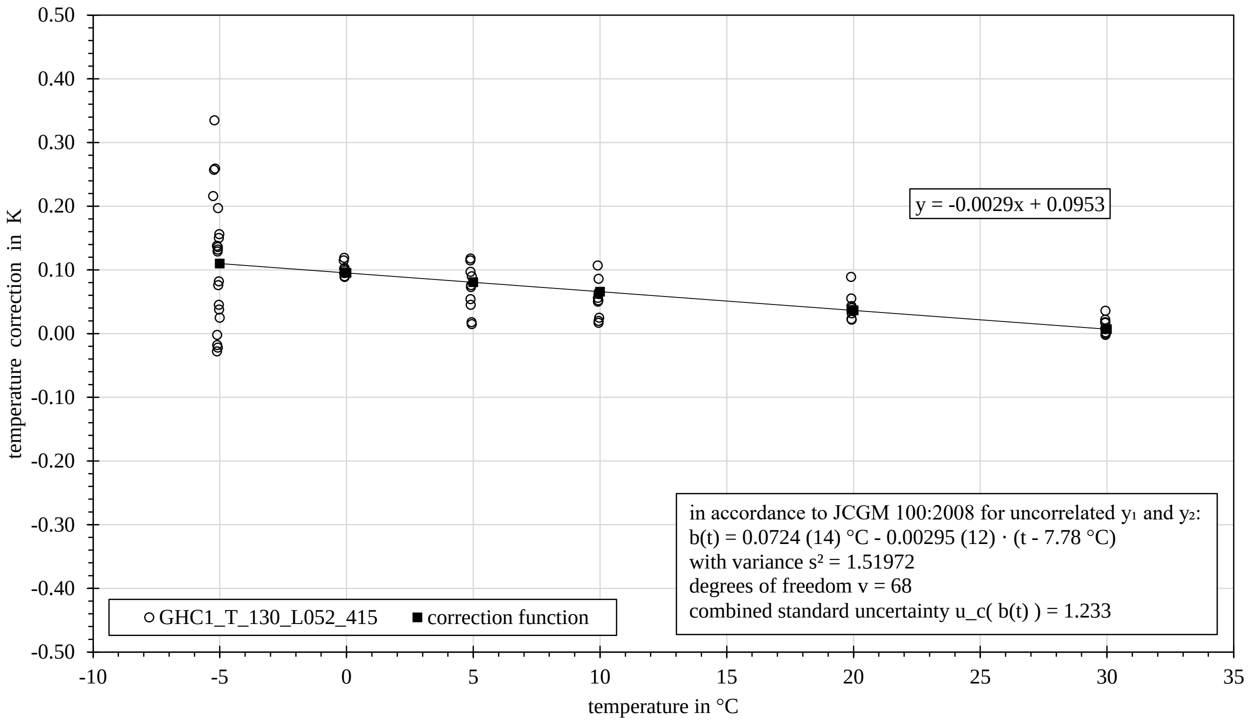

Figure 22: necessary correction of GHC1_T_130_L052_415 defined by calibration of the temperature sensor

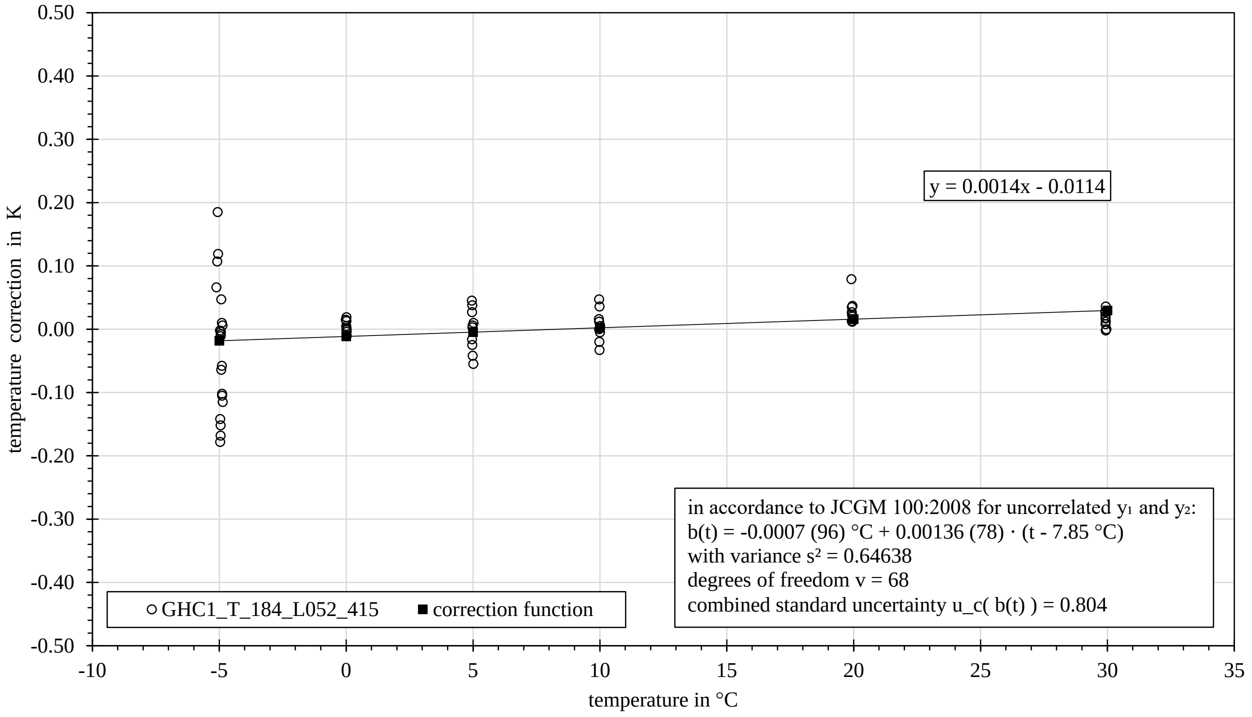

Figure 23: necessary correction of GHC1_T_184_L052_415 defined by calibration of the temperature sensor

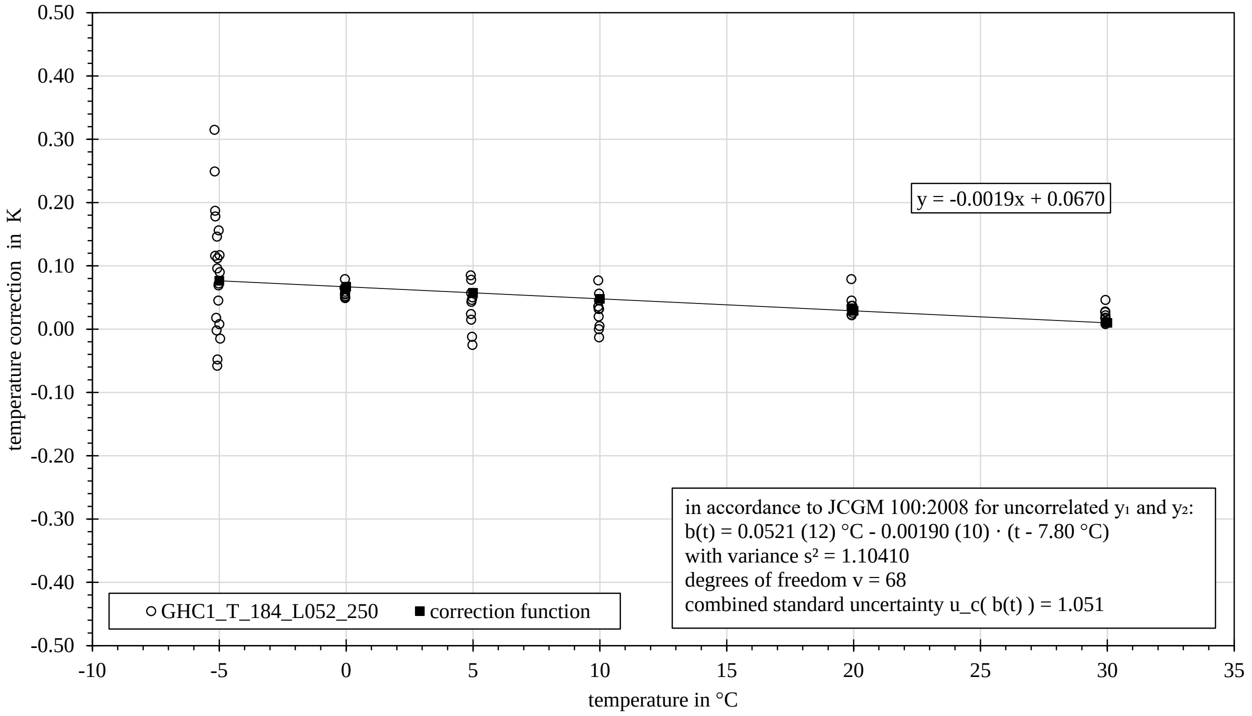

Figure 24: necessary correction of GHC1_T_184_L052_250 defined by calibration of the temperature sensor

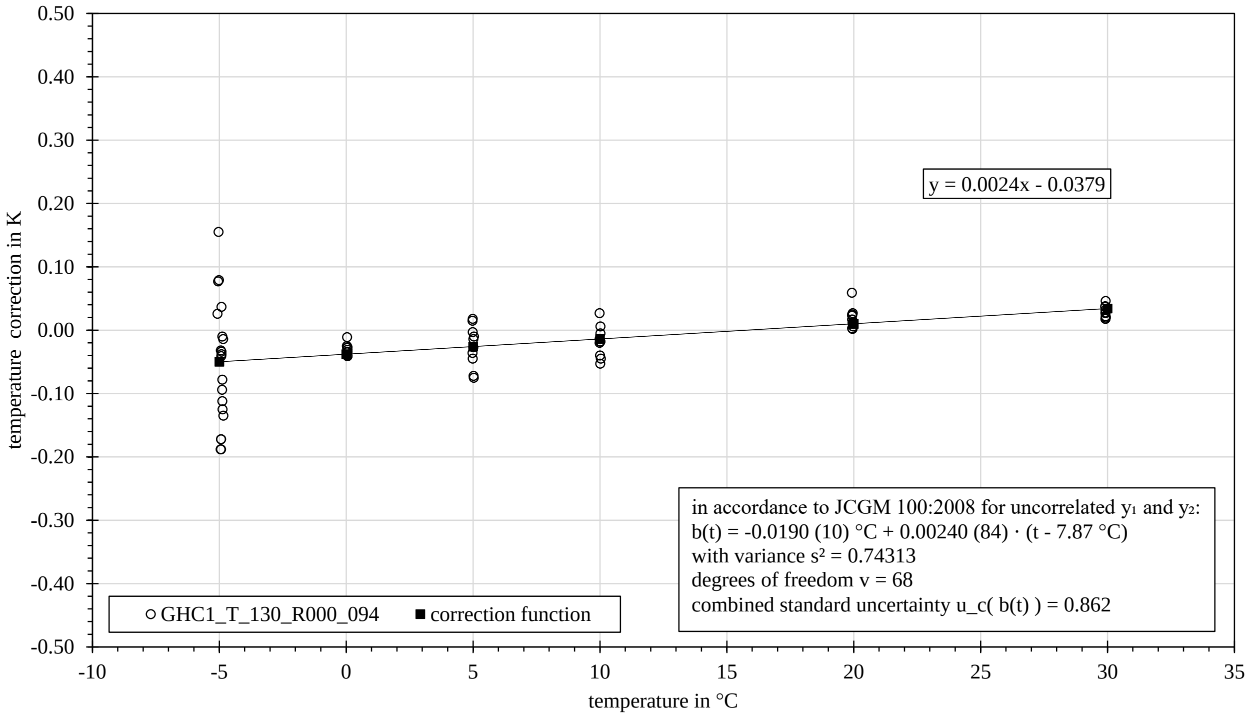

Figure 25: necessary correction of GHC1_T_130_R000_094 defined by calibration of the temperature sensor

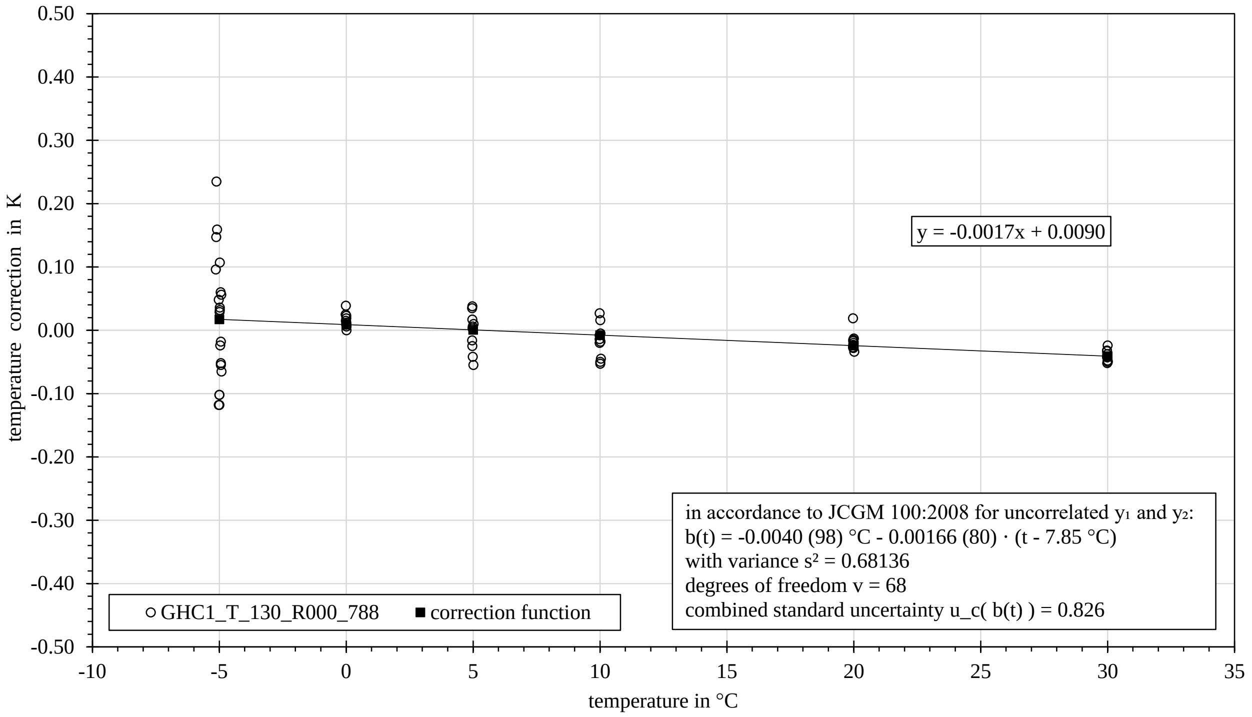

Figure 26: necessary correction of GHC1_T_130_R000_788 defined by calibration of the temperature sensor

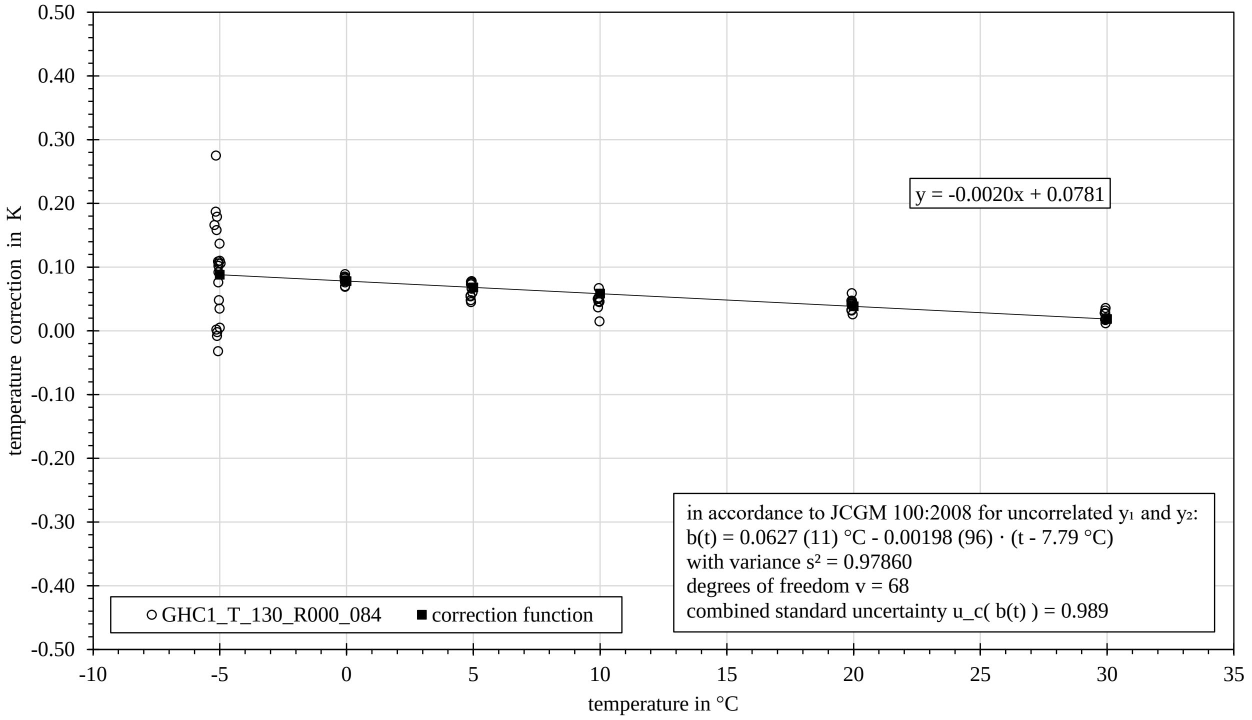

Figure 27: necessary correction of GHC1_T_130_R000_084 defined by calibration of the temperature sensor

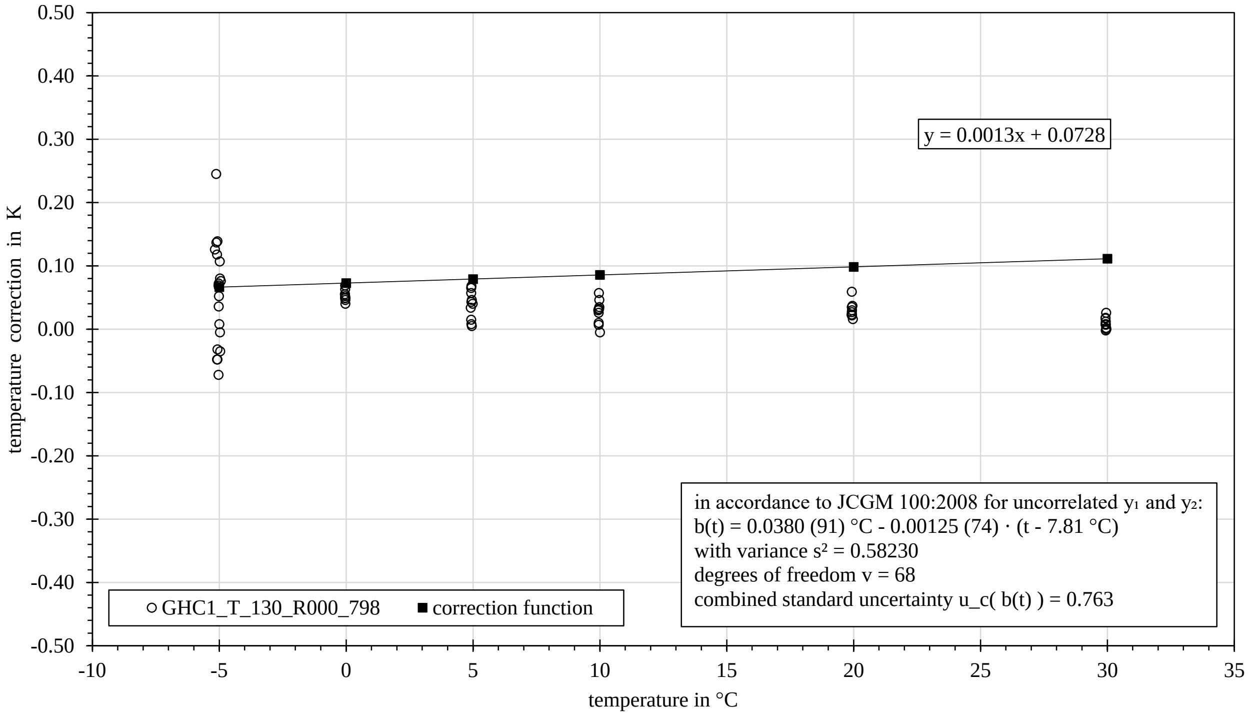

Figure 28: necessary correction of GHC1_T_130_R000_798 defined by calibration of the temperature sensor

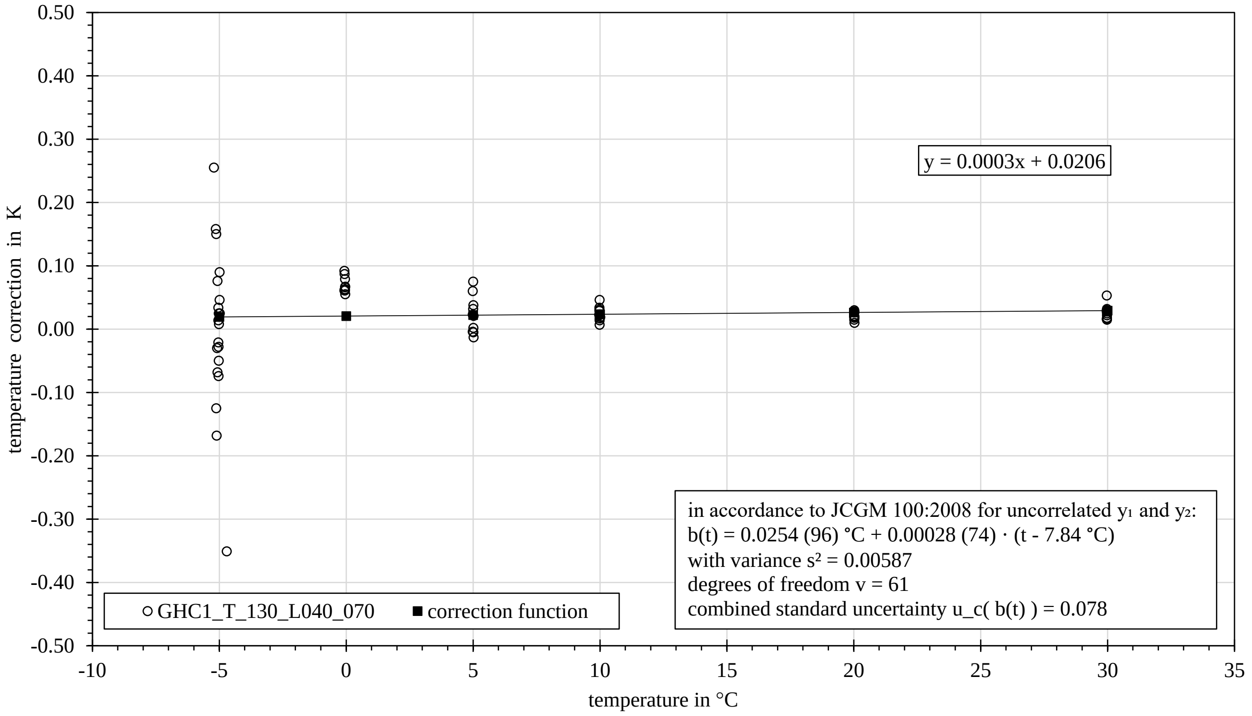

Figure 29: necessary correction of GHC1_T_130_L040_070 defined by calibration of the temperature sensor

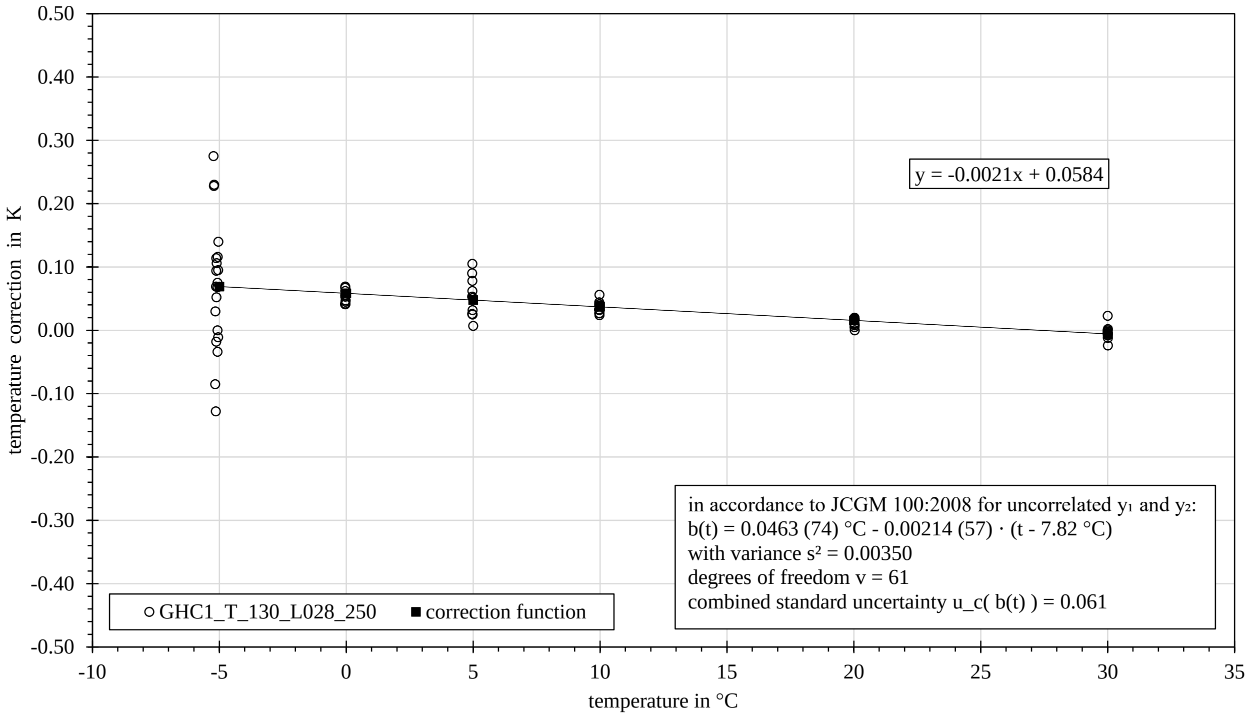

Figure 30: necessary correction of GHC1_T_130_L028_250 defined by calibration of the temperature sensor

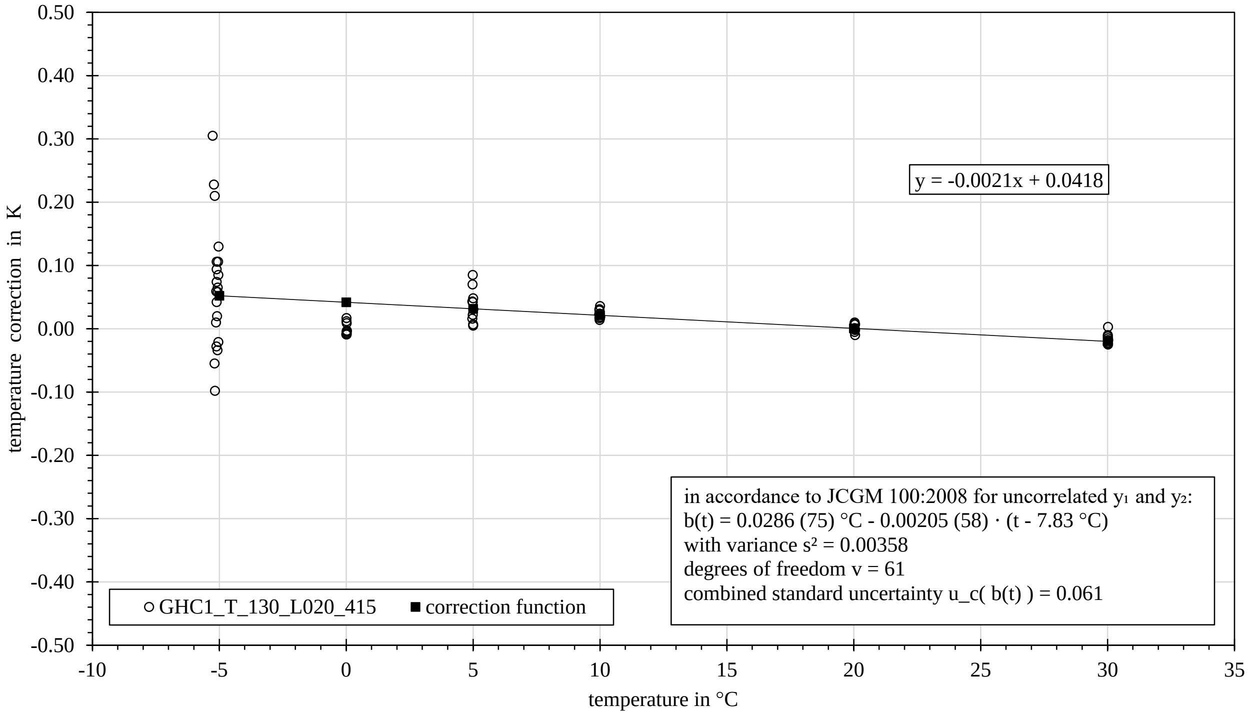

Figure 31: necessary correction of GHC1_T_130_L020_415 defined by calibration of the temperature sensor

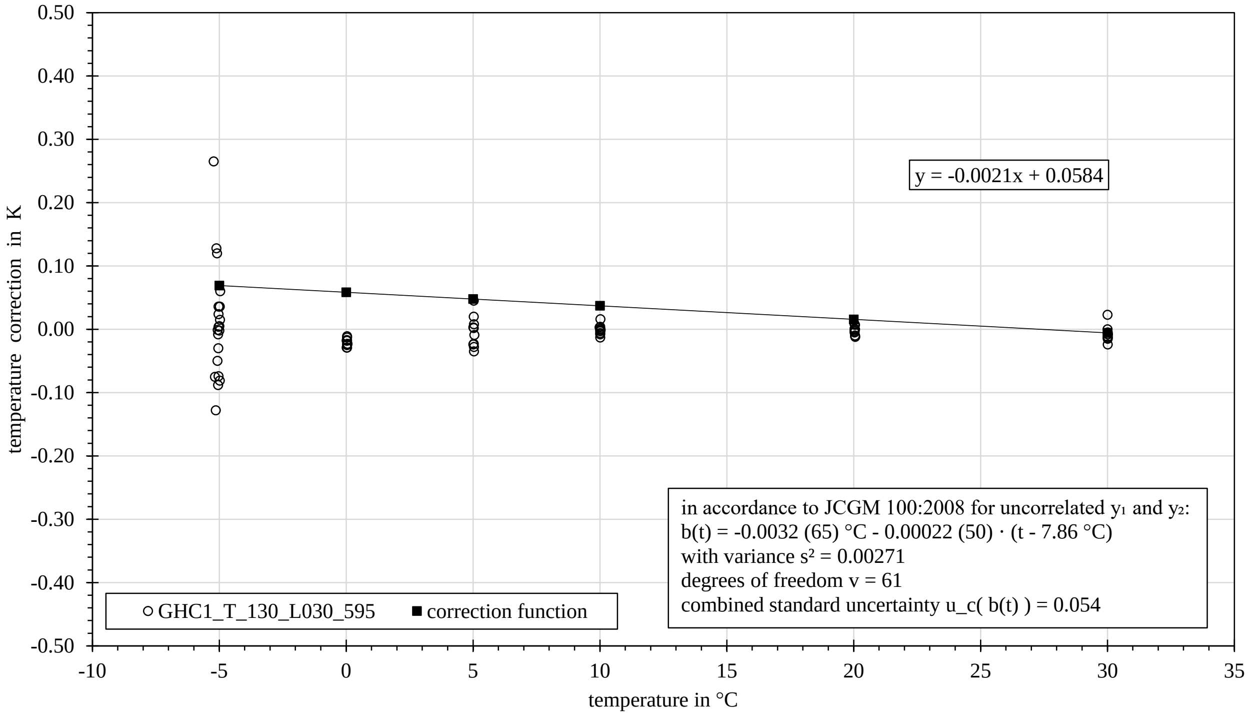

Figure 32: necessary correction of GHC1_T_130_L030_595 defined by calibration of the temperature sensor

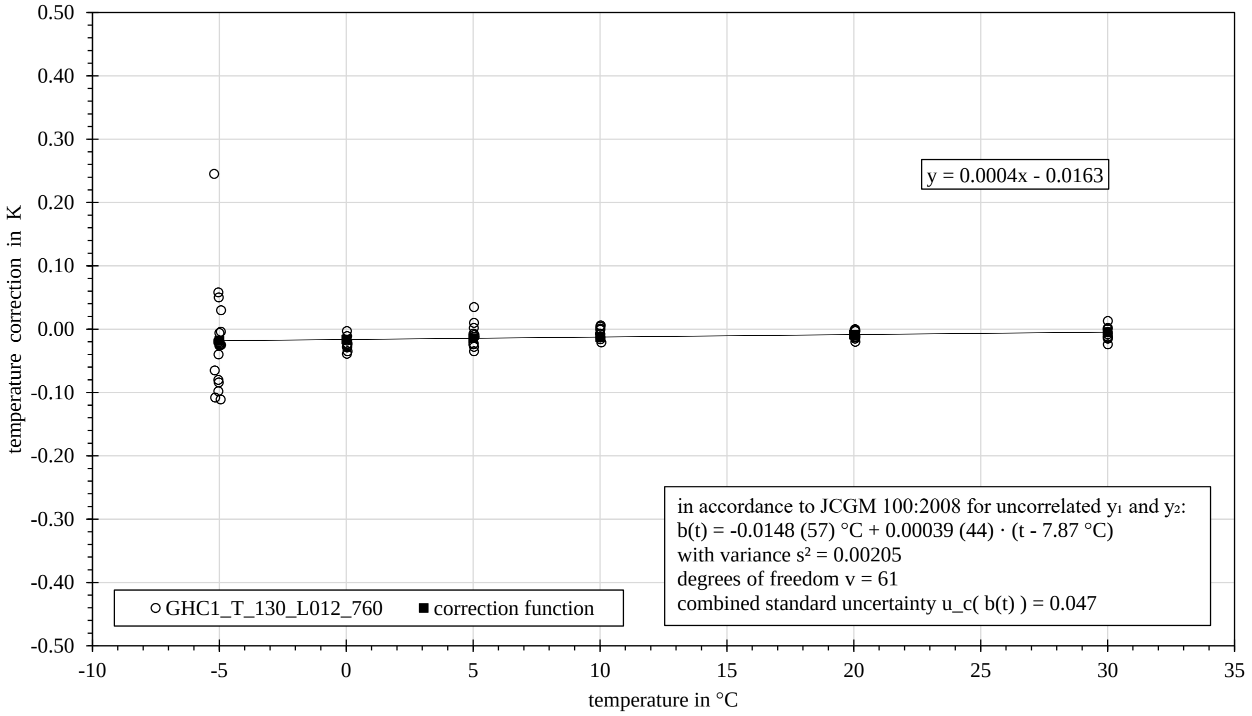

Figure 33: necessary correction of GHC1_T_130_L012_760 defined by calibration of the temperature sensor

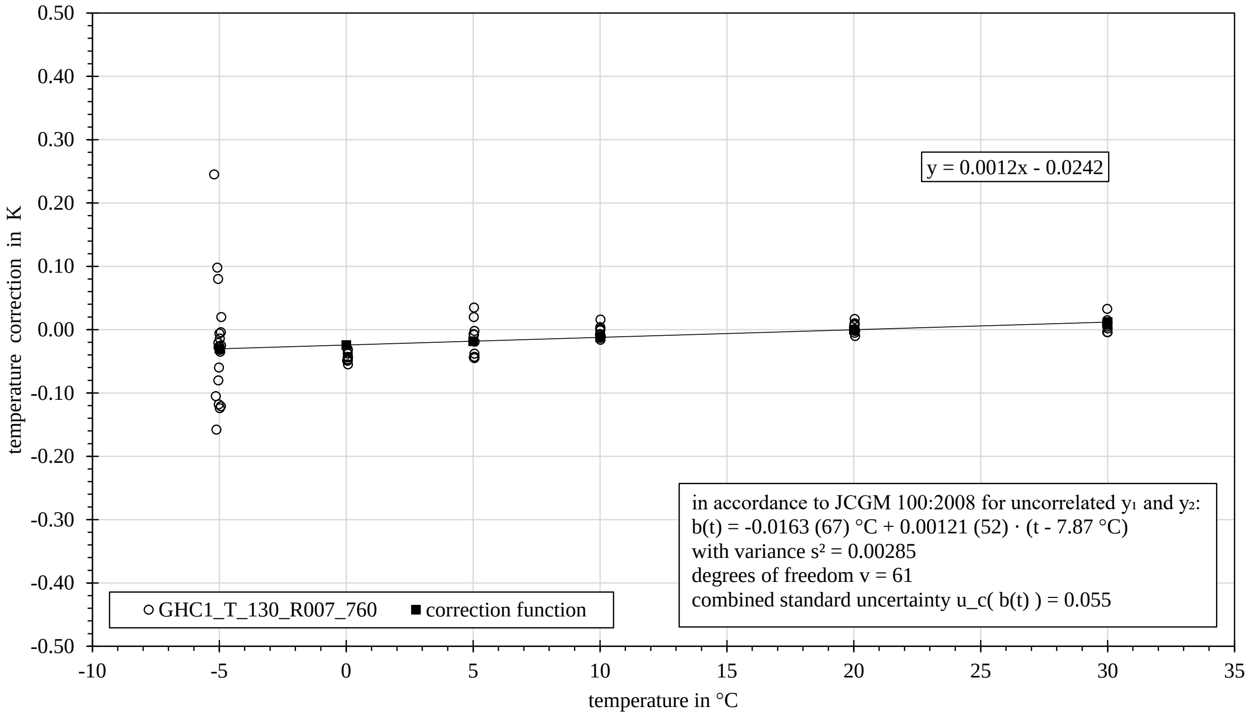

Figure 34: necessary correction of GHC1_T_130_R007_760 defined by calibration of the temperature sensor

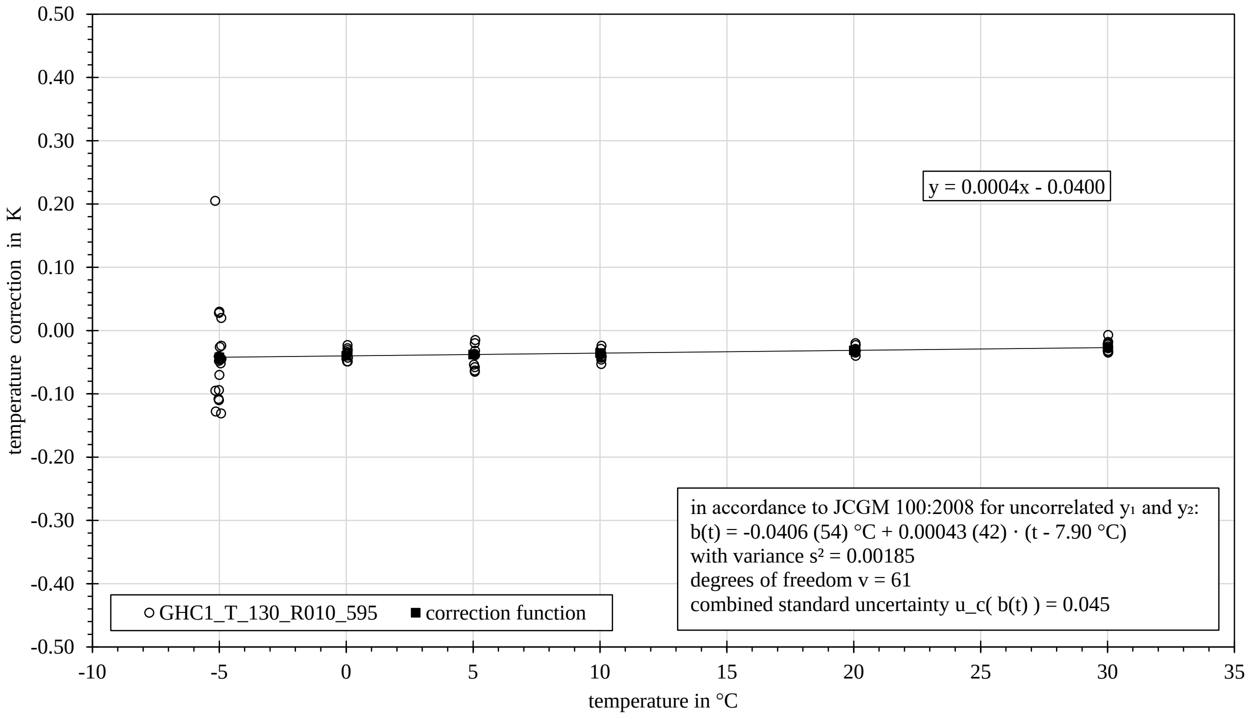

Figure 35: necessary correction of GHC1_T_130_R010_595 defined by calibration of the temperature sensor

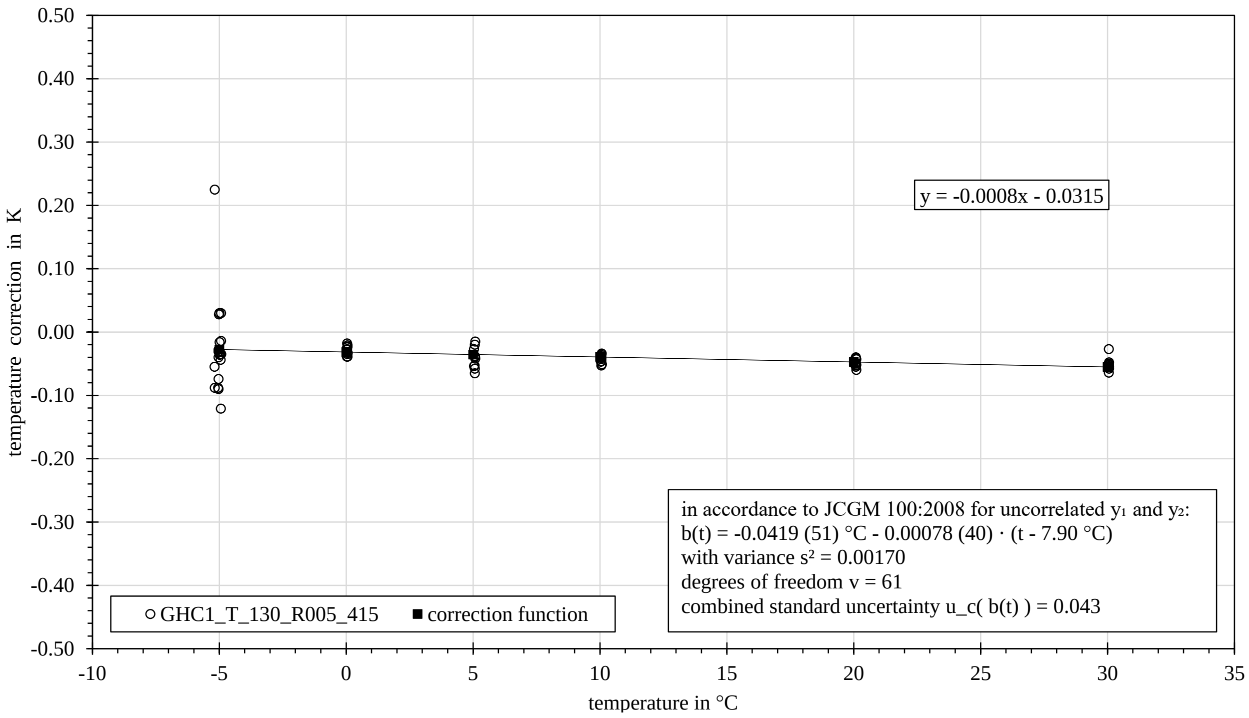

Figure 36: necessary correction of GHC1_T_130_R005_415 defined by calibration of the temperature sensor

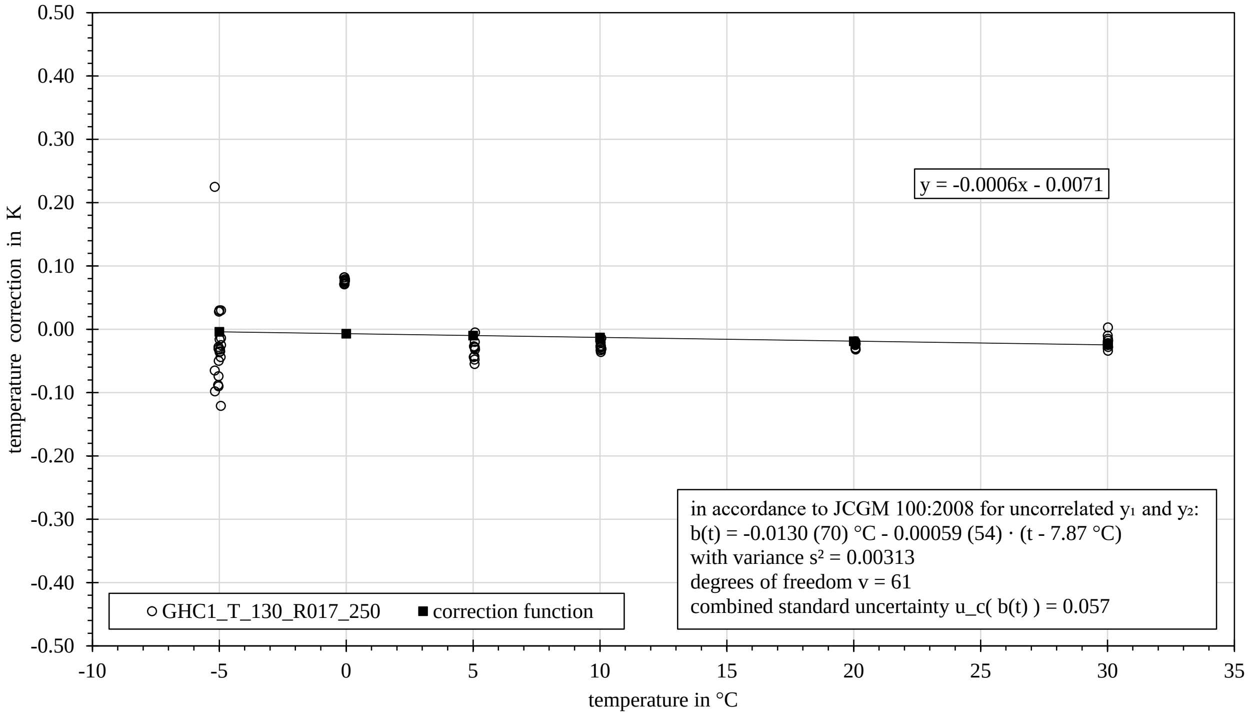

Figure 37: necessary correction of GHC1_T_130_R017_250 defined by calibration of the temperature sensor

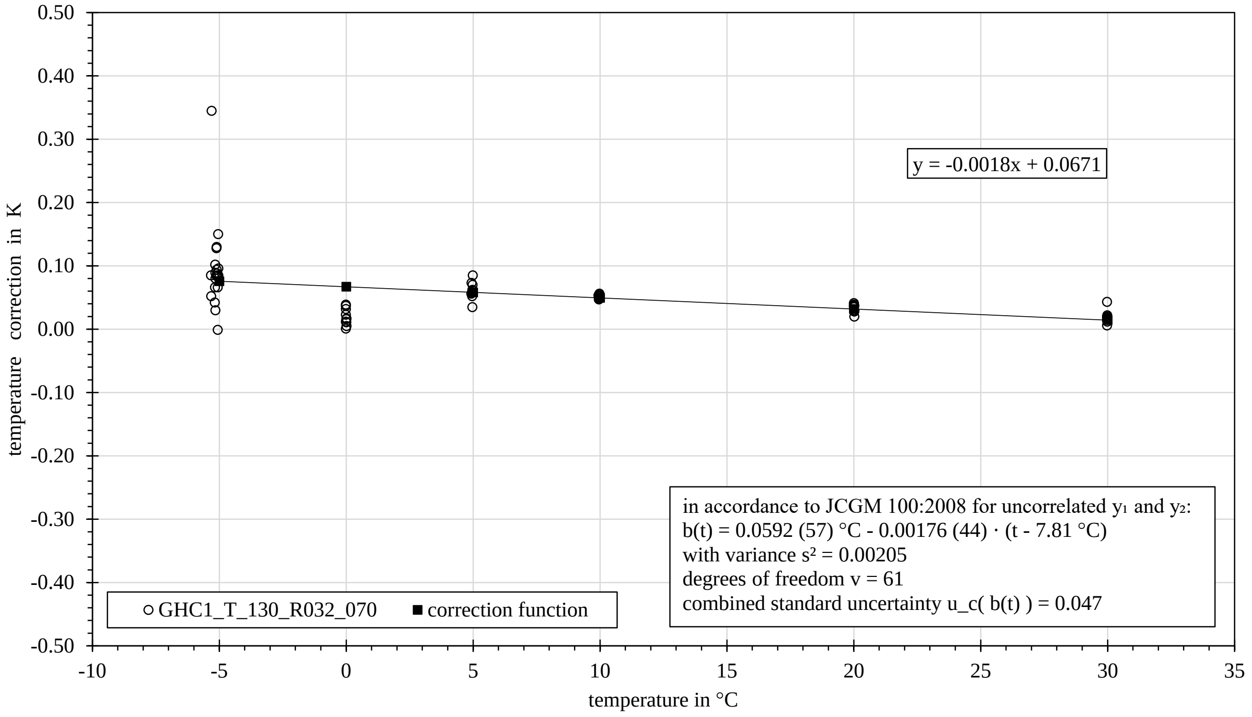

Figure 38: necessary correction of GHC1_T_130_R032_070 defined by calibration of the temperature sensor

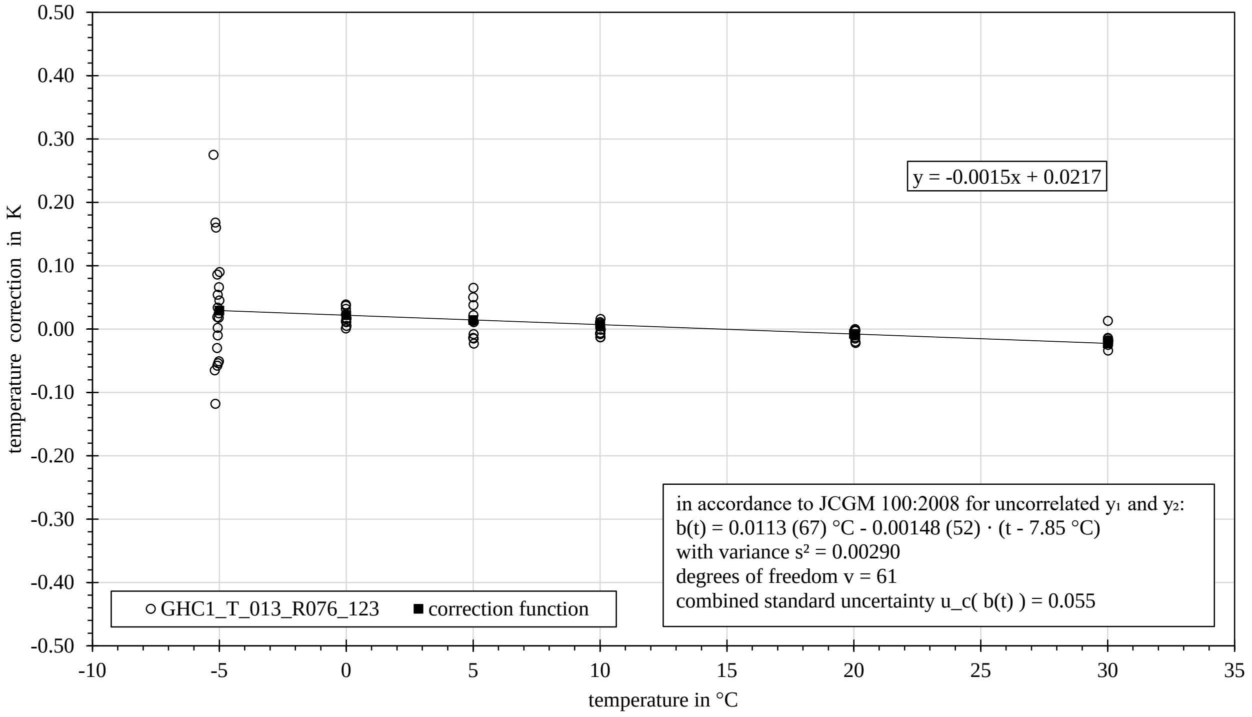

Figure 39: necessary correction of GHC1_T_013_R076_123 defined by calibration of the temperature sensor

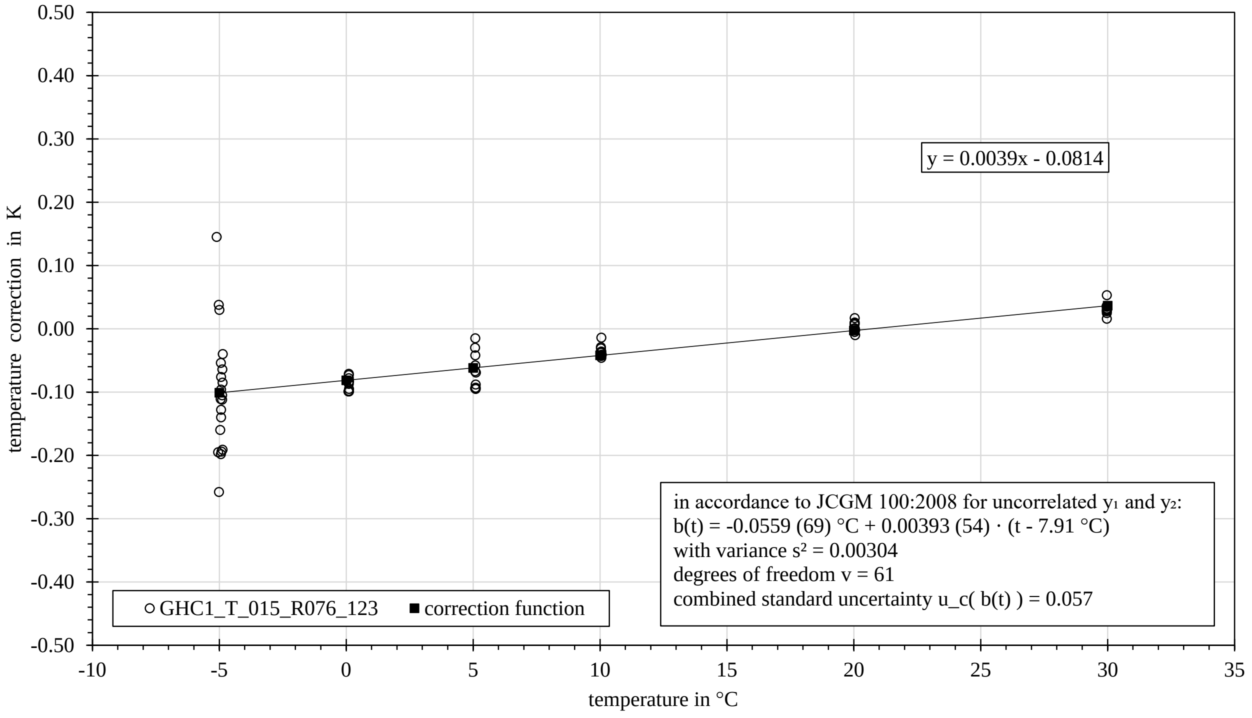

Figure 40: necessary correction of GHC1_T_015_R076_123 defined by calibration of the temperature sensor

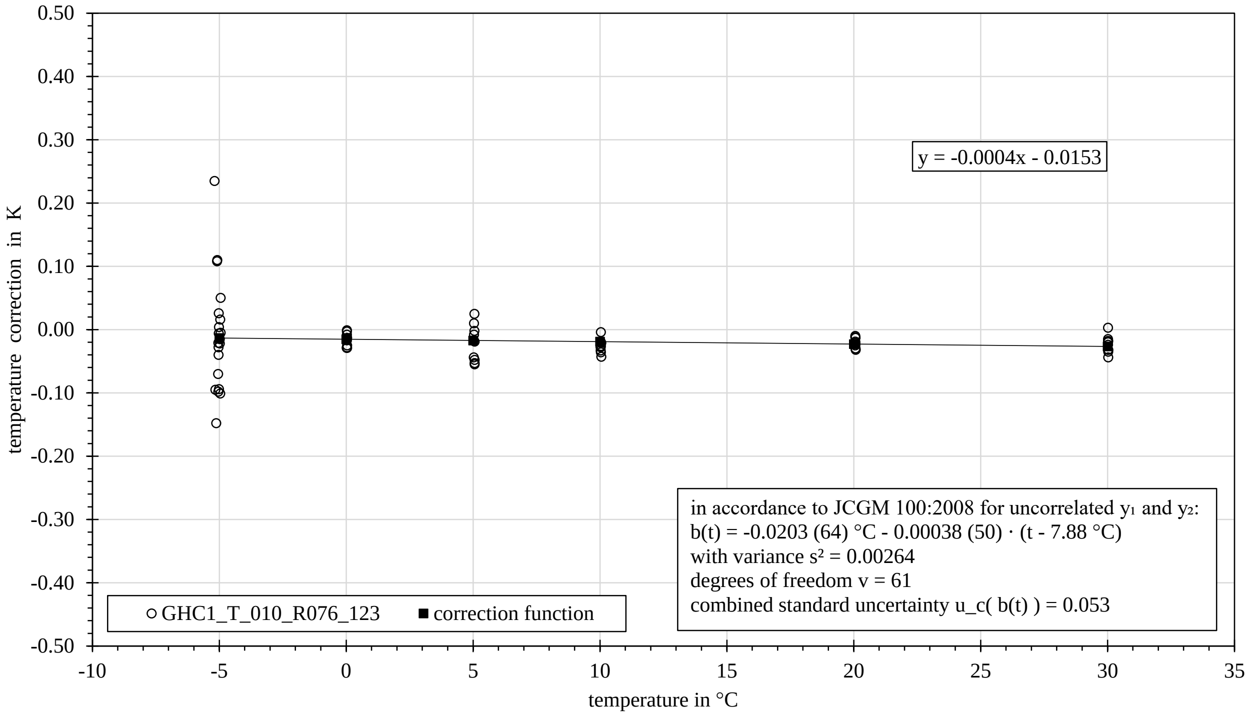

Figure 41: necessary correction of GHC1_T_010_R076_123 defined by calibration of the temperature sensor

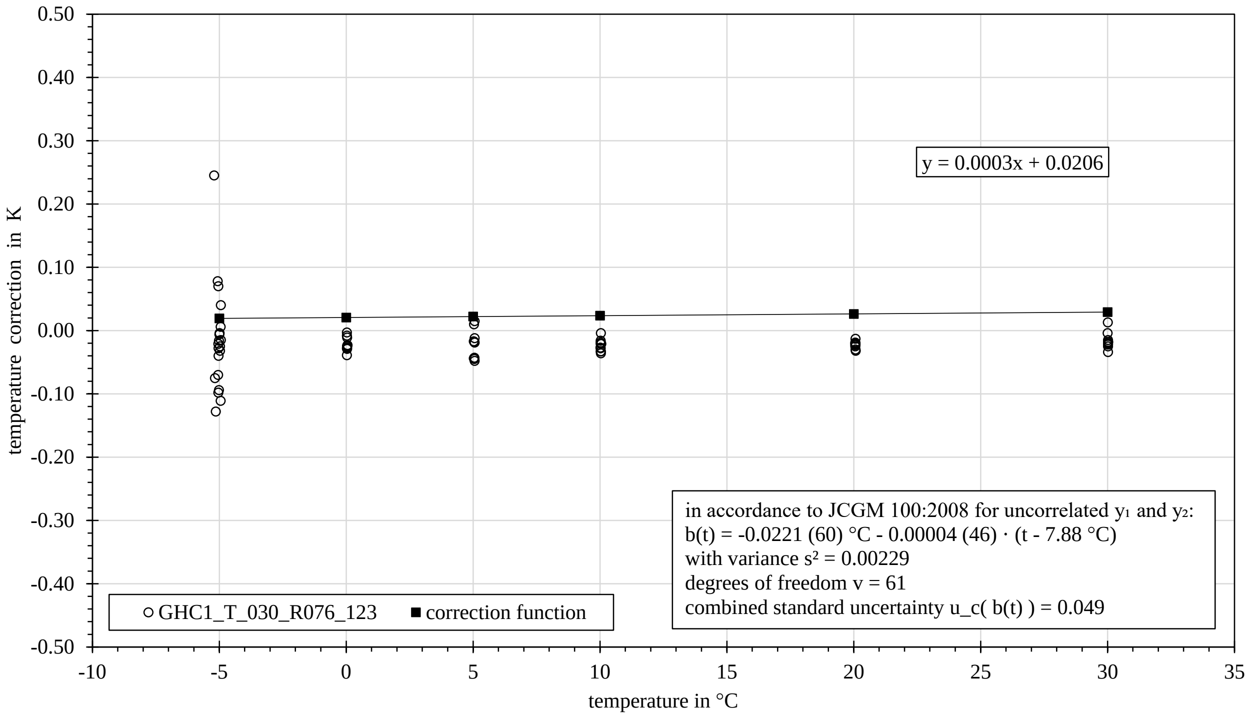

Figure 42: necessary correction of GHC1_T_030_R076_123 defined by calibration of the temperature sensor

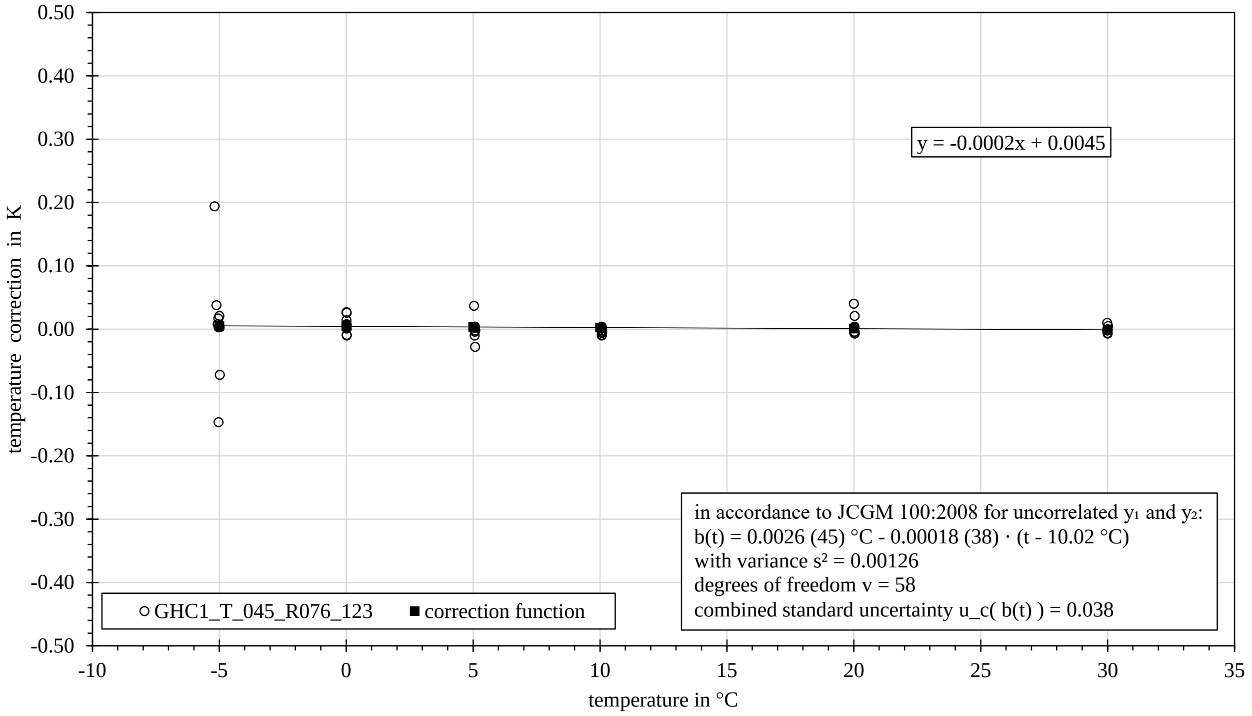

Figure 43: necessary correction of GHC1_T_045_R076_123 defined by calibration of the temperature sensor

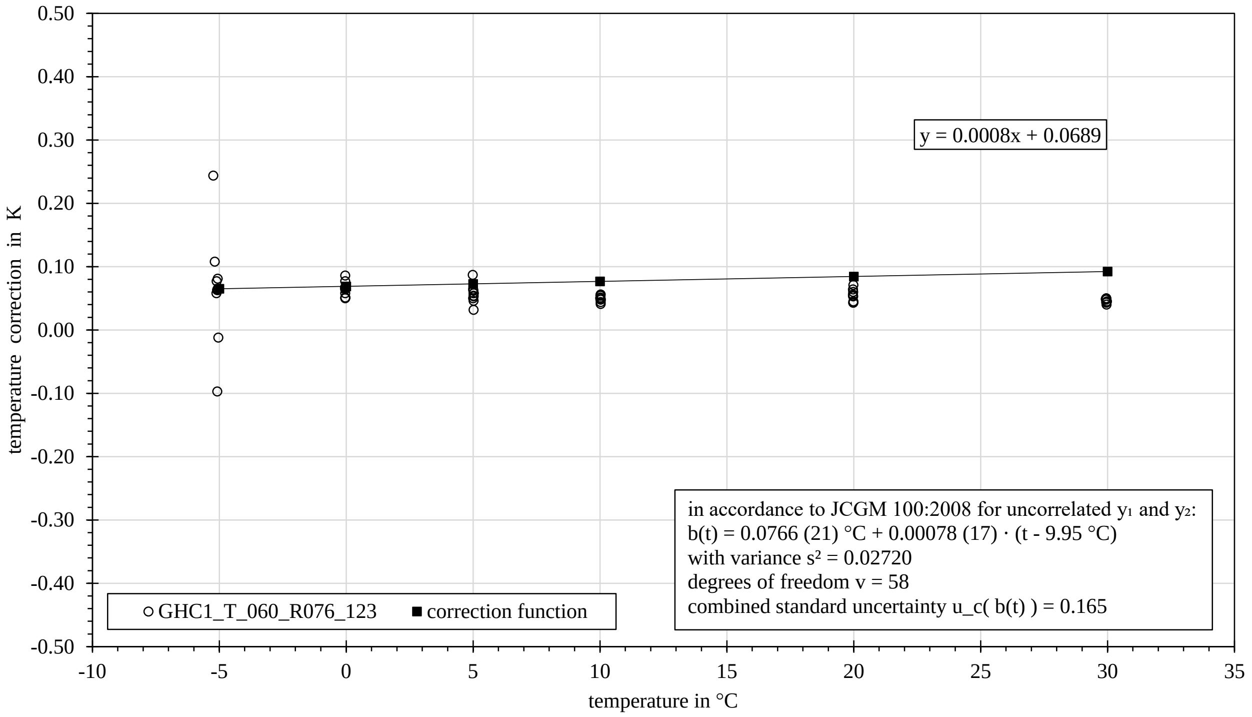

Figure 44: necessary correction of GHC1_T_060_R076_123 defined by calibration of the temperature sensor

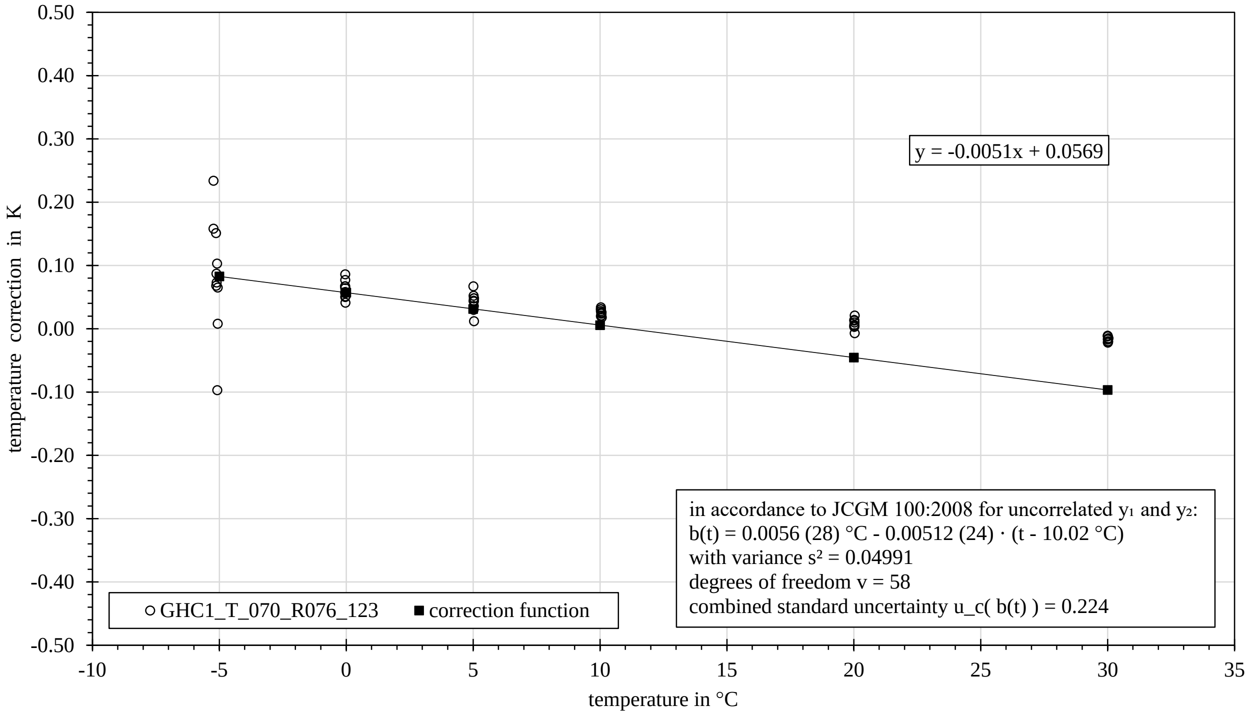

Figure 45: necessary correction of GHC1_T_070_R076_123 defined by calibration of the temperature sensor

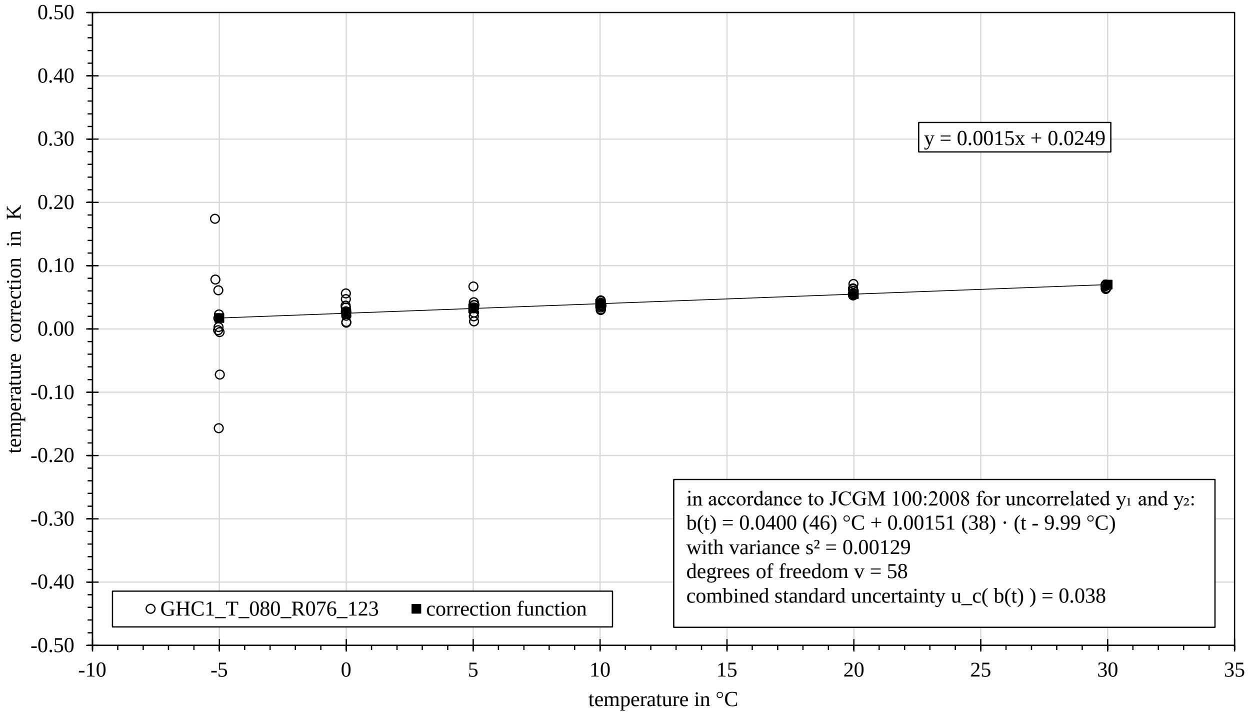

Figure 46: necessary correction of GHC1_T_080_R076_123 defined by calibration of the temperature sensor

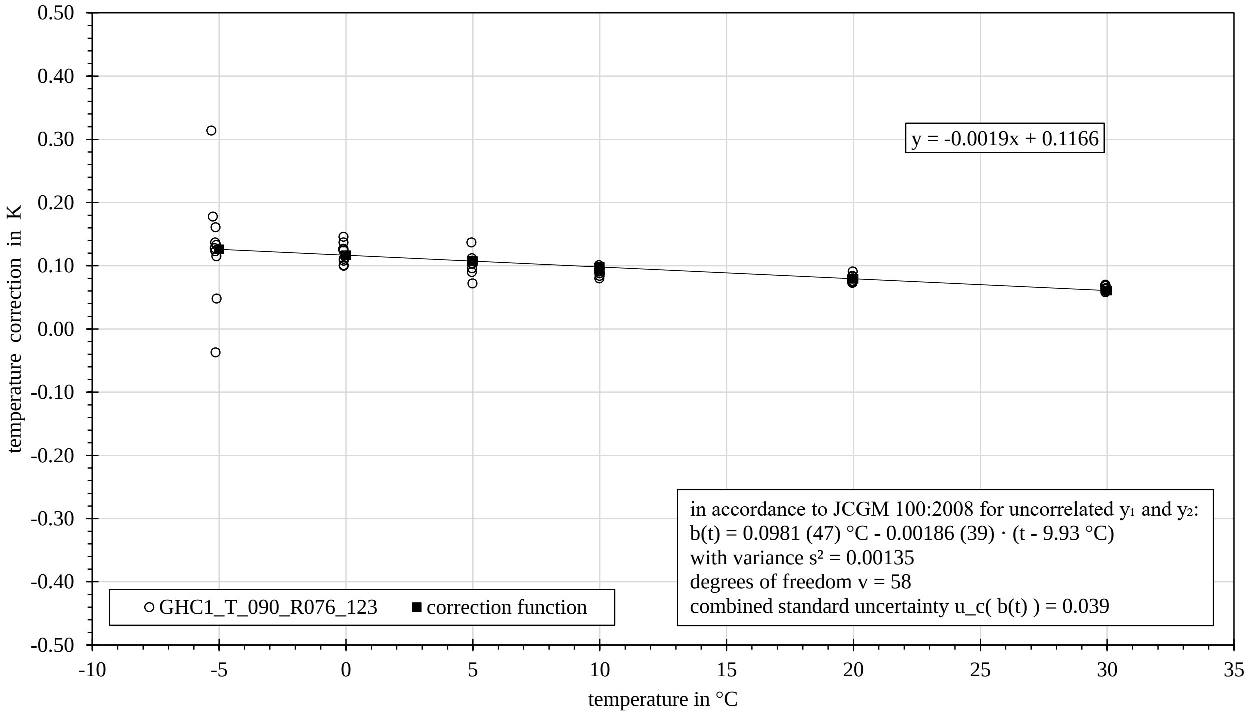

Figure 47: necessary correction of GHC1_T_090_R076_123 defined by calibration of the temperature sensor

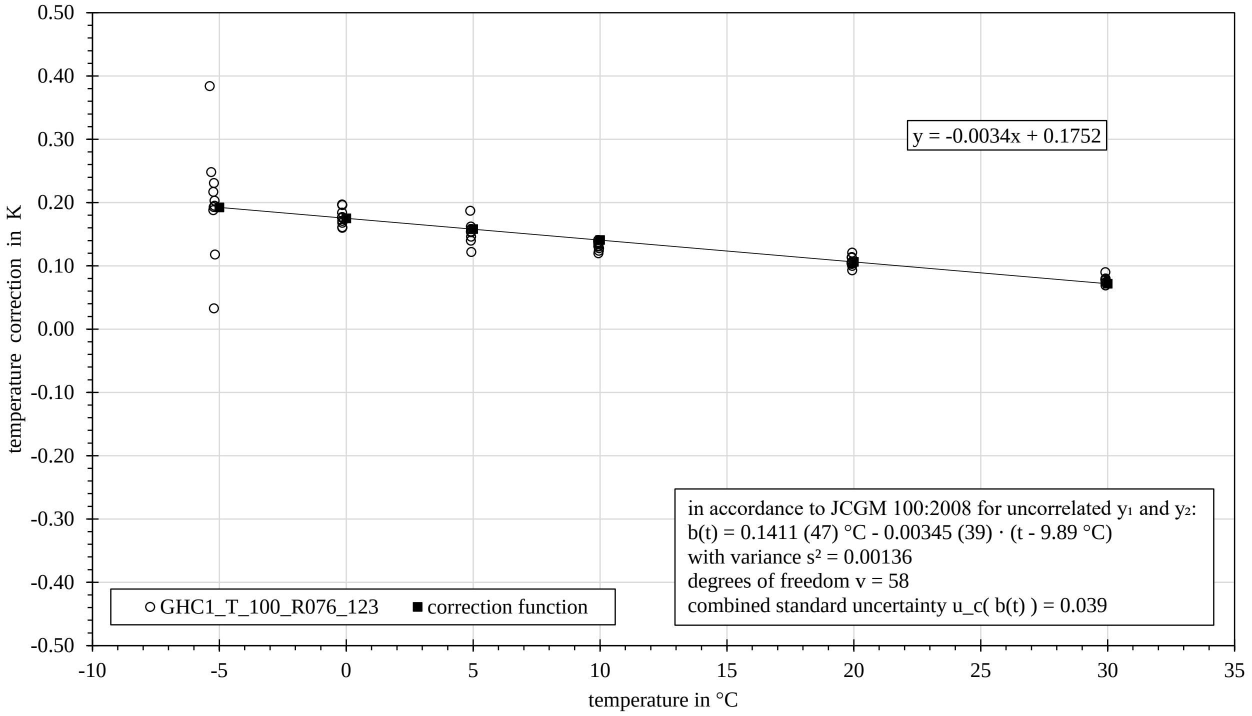

Figure 48: necessary correction of GHC1_T_100_R076_123 defined by calibration of the temperature sensor

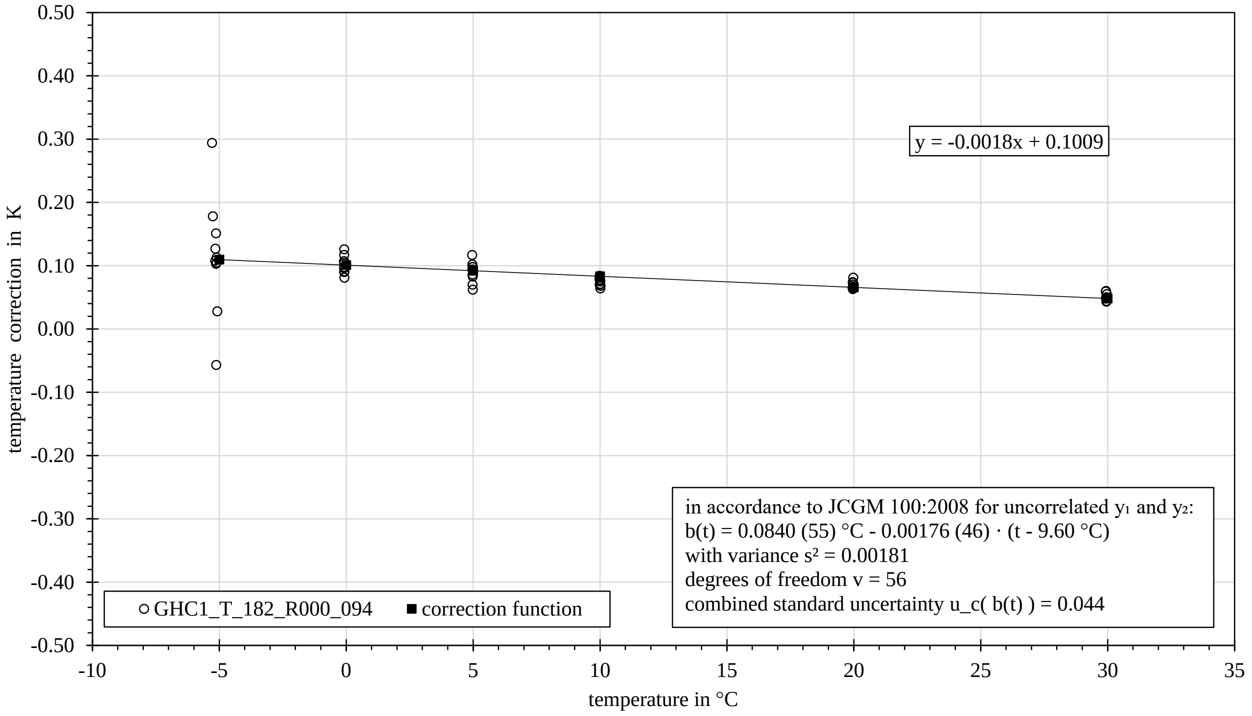

Figure 49: necessary correction of GHC1_T_182_R000_094 defined by calibration of the temperature sensor

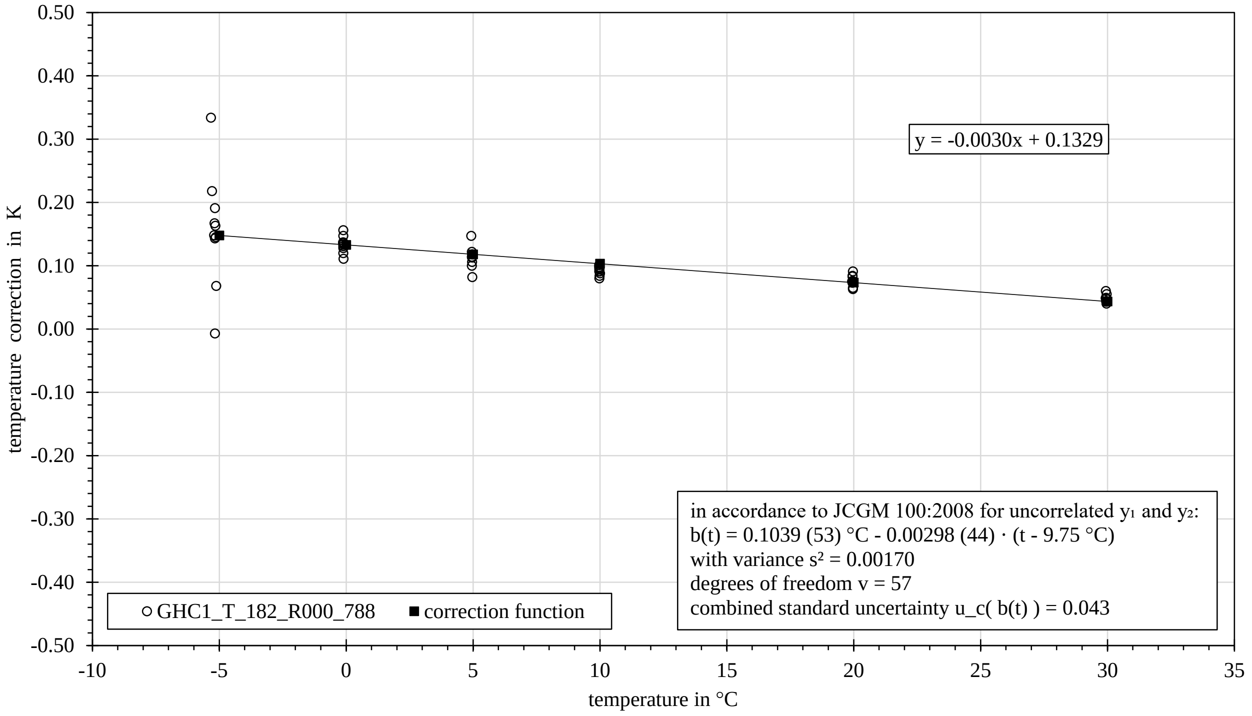

Figure 50: necessary correction of GHC1_T_182_R000_788 defined by calibration of the temperature sensor

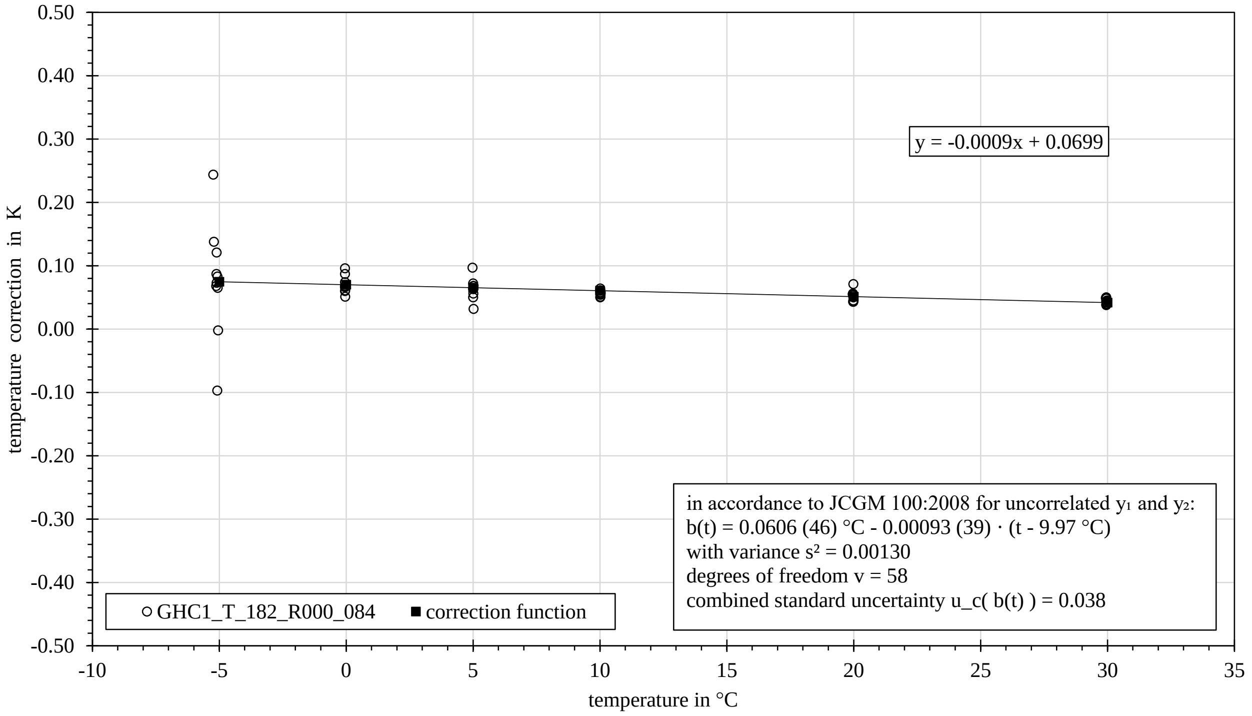

Figure 51: necessary correction of GHC1_T_182_R000_084 defined by calibration of the temperature sensor

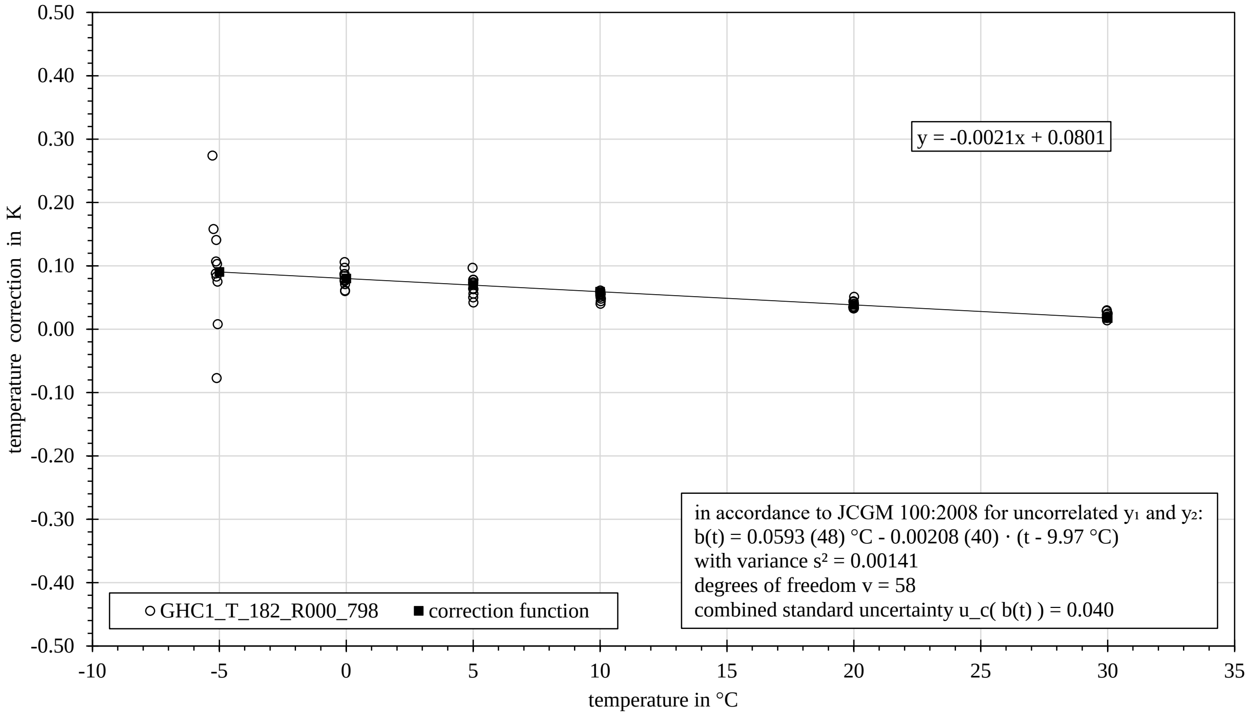

Figure 52: necessary correction of GHC1_T_182_R000_798 defined by calibration of the temperature sensor

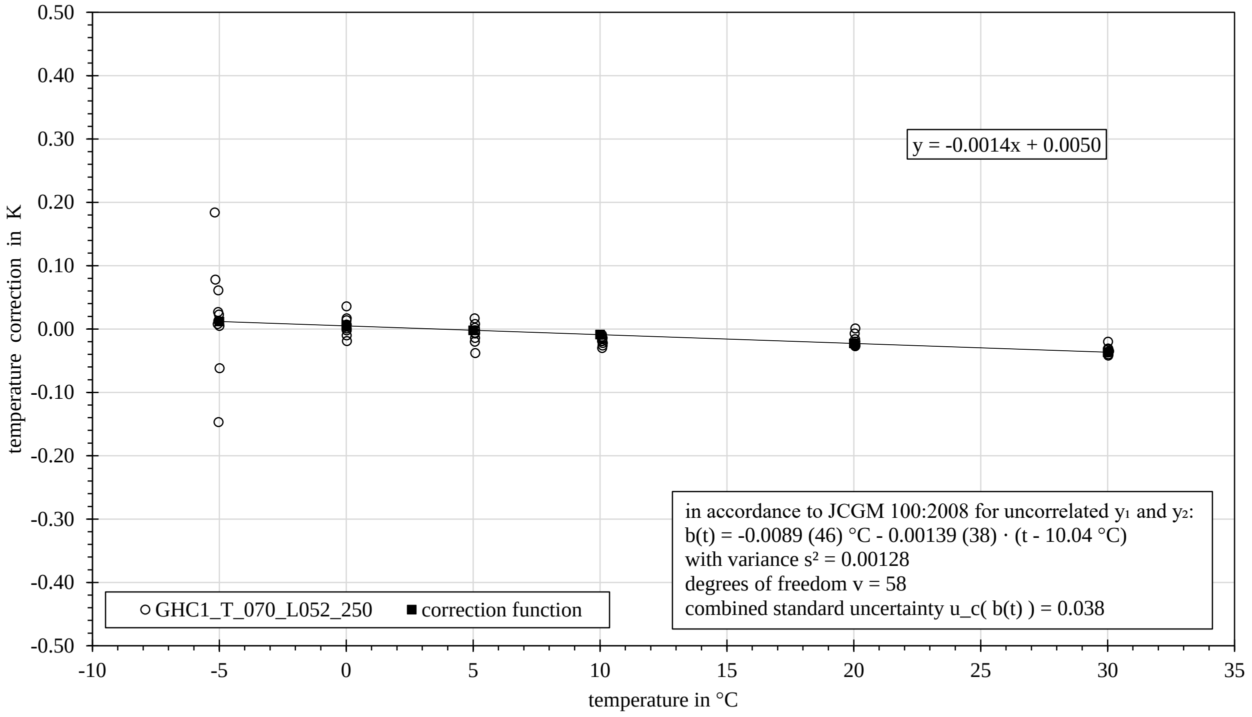

Figure 53: necessary correction of GHC1_T_070_L052_250 defined by calibration of the temperature sensor

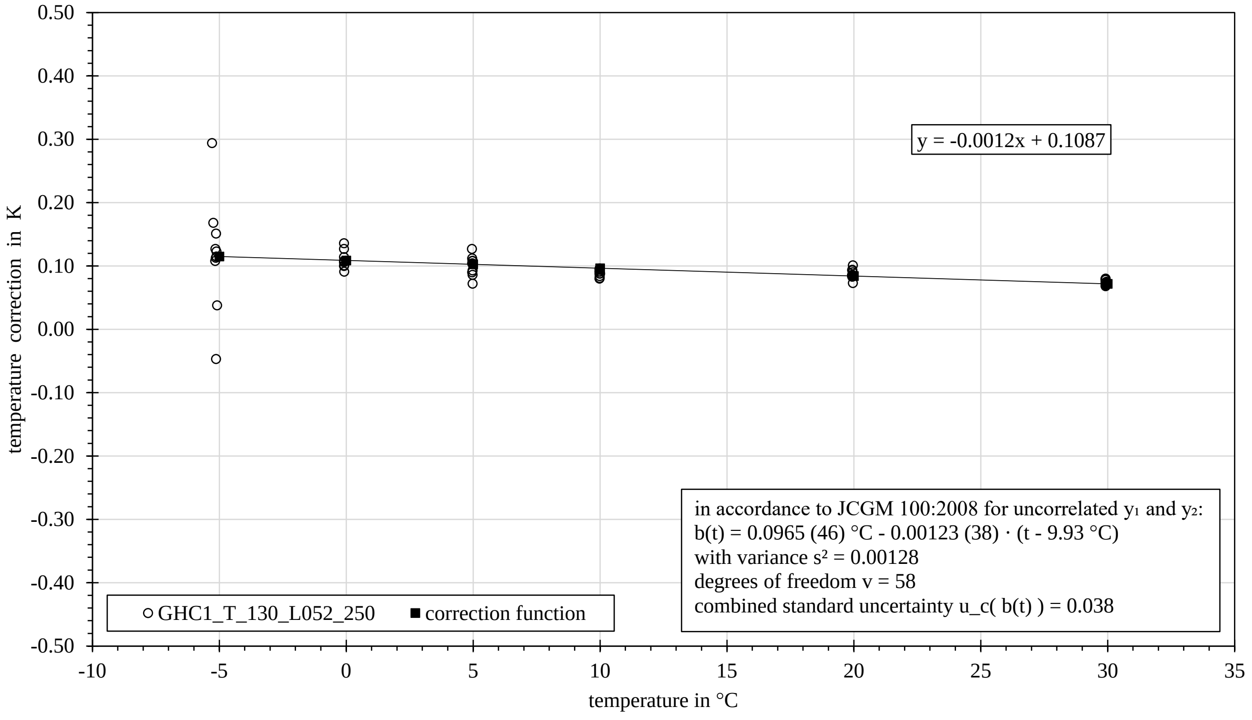

Figure 54: necessary correction of GHC1_T_130_L052_250 defined by calibration of the temperature sensor

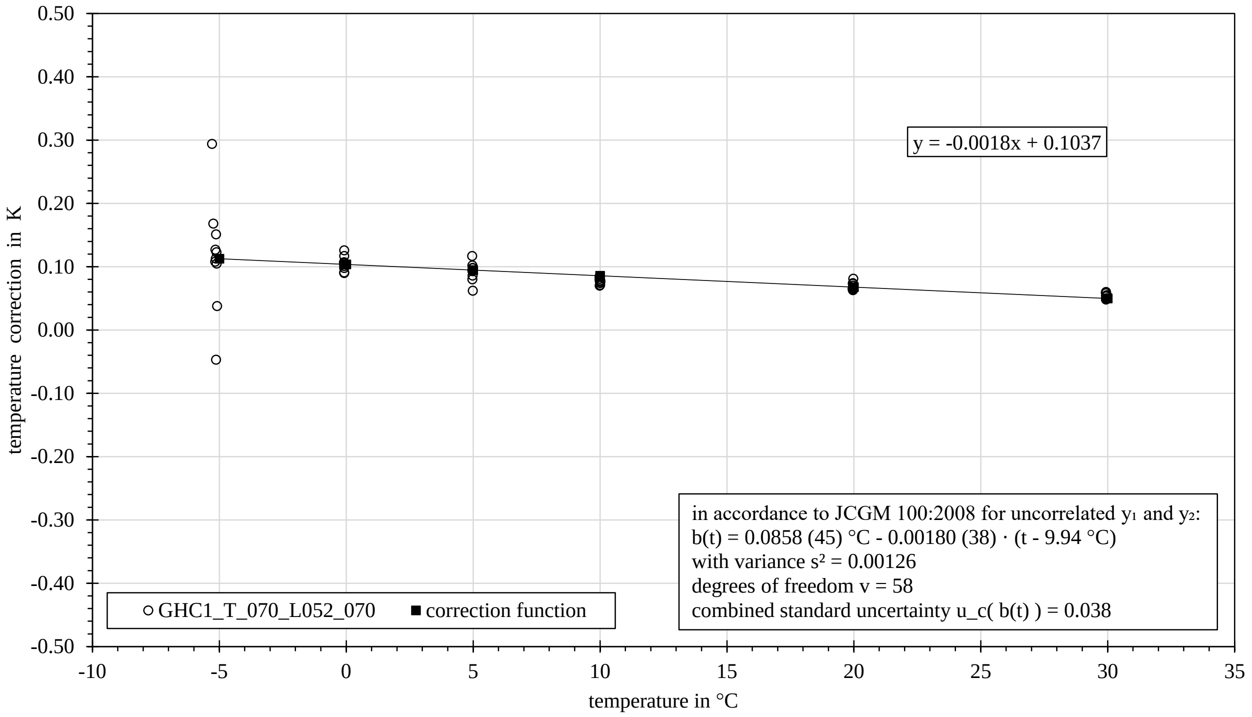

Figure 55: necessary correction of GHC1_T_070_L052_070 defined by calibration of the temperature sensor

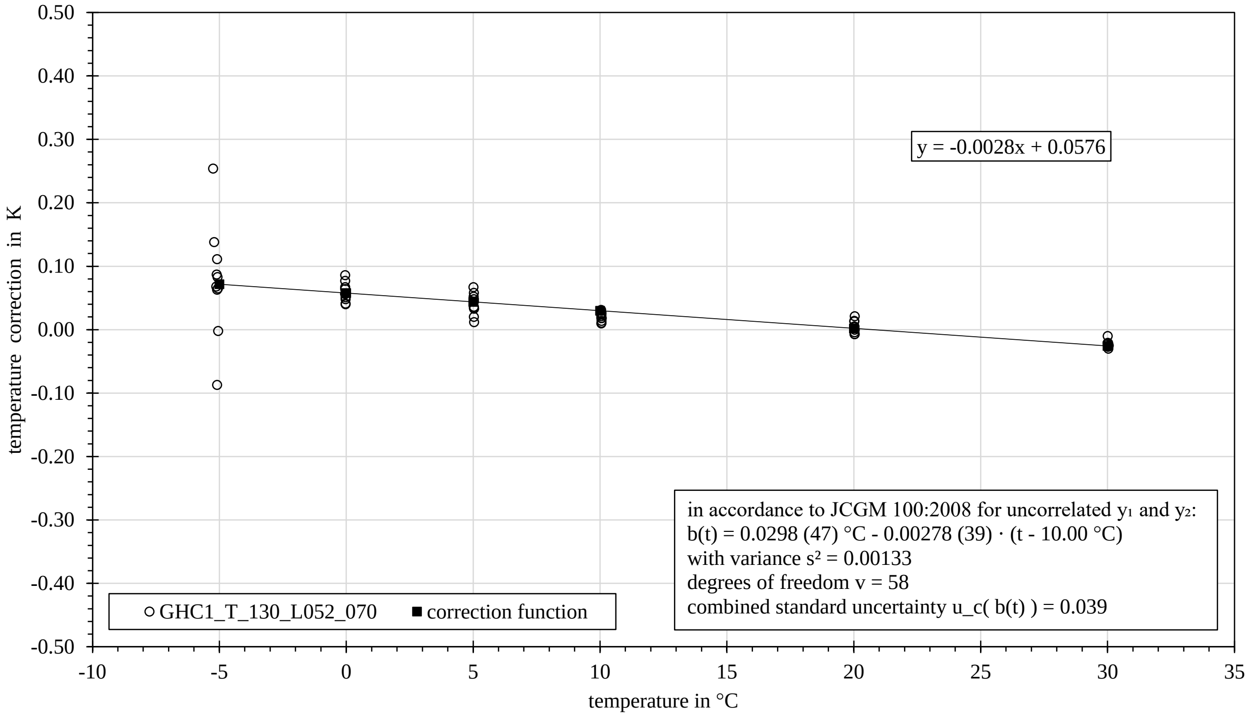

Figure 56: necessary correction of GHC1_T_130_L052_070 defined by calibration of the temperature sensor

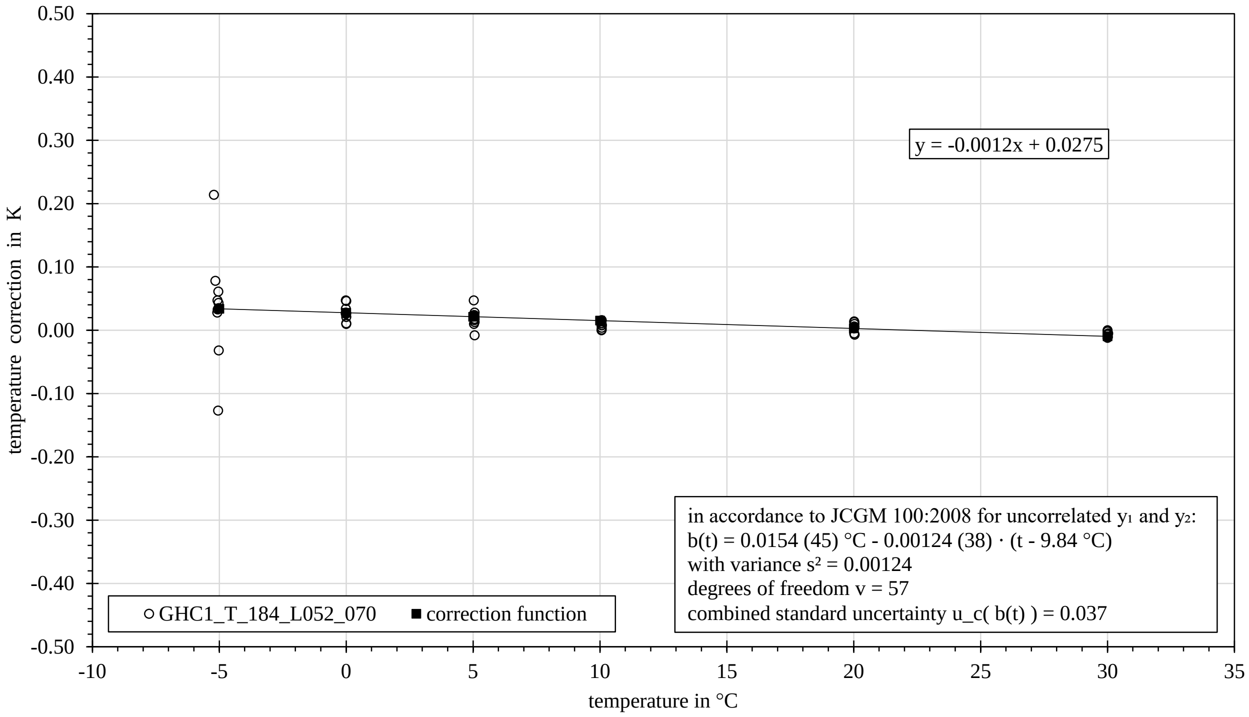

Figure 57: necessary correction of GHC1_T_184_L052_070 defined by calibration of the temperature sensor

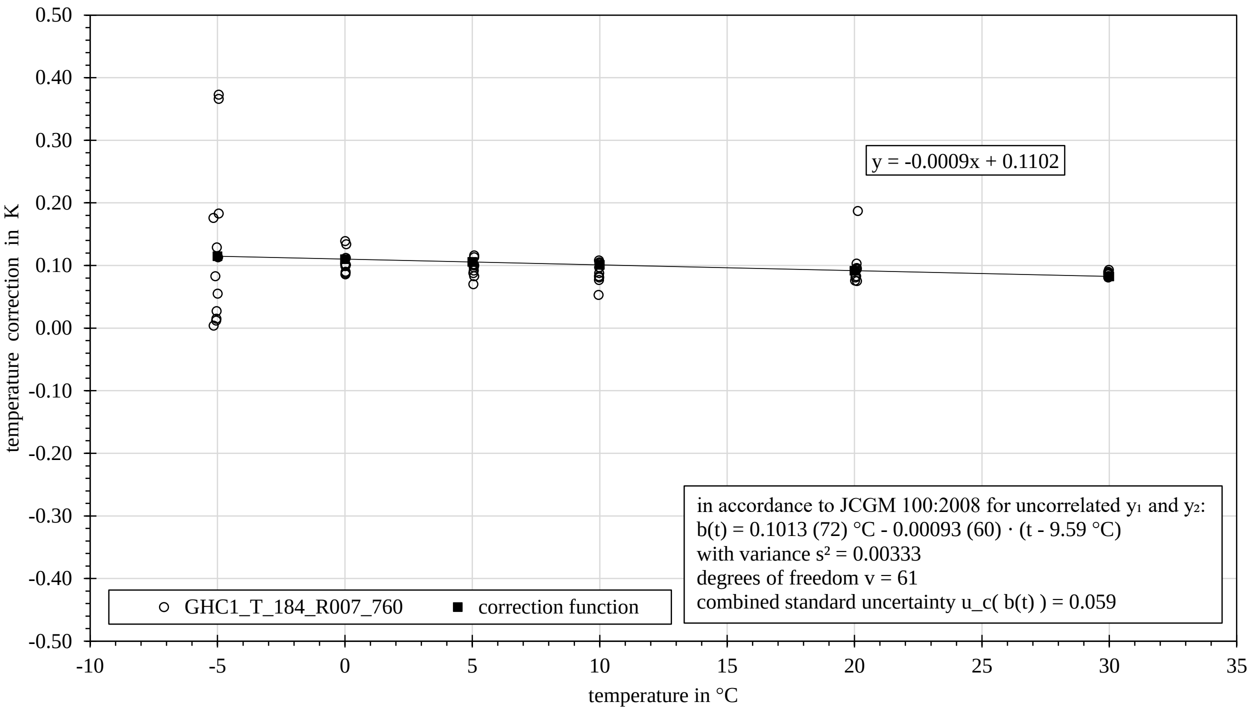

Figure 58: necessary correction of GHC1_T_184_R007_760 defined by calibration of the temperature sensor

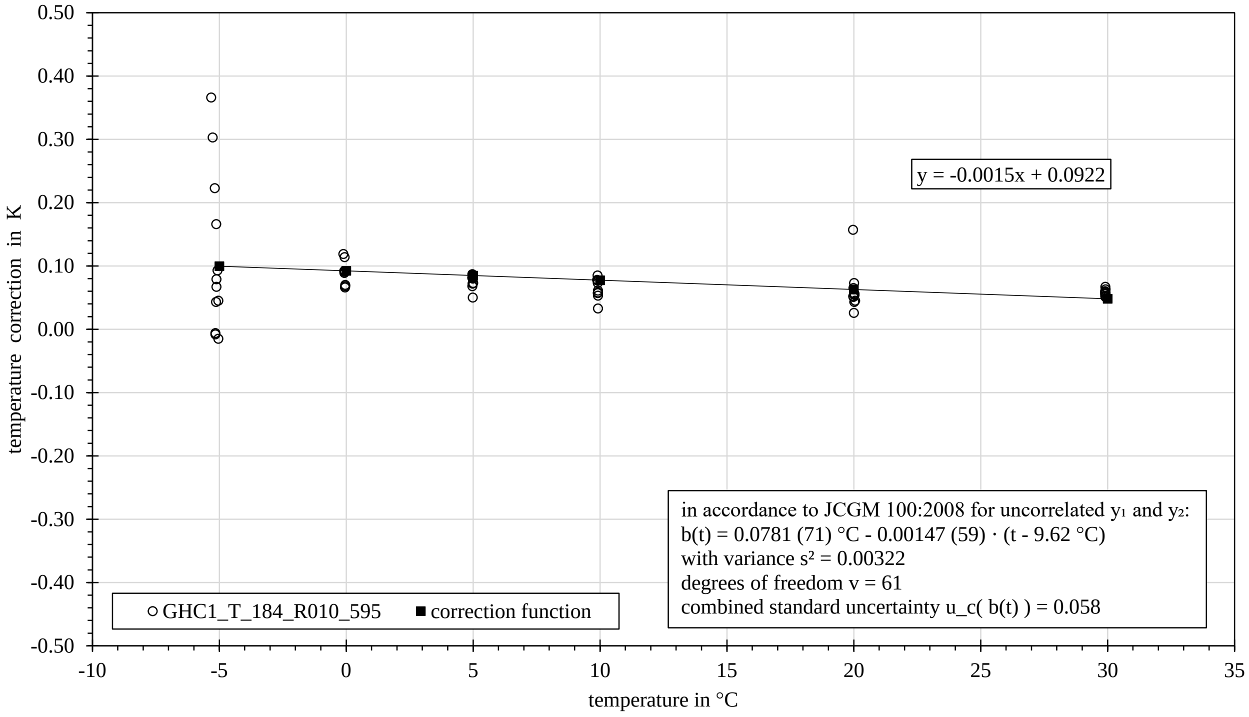

Figure 59: necessary correction of GHC1_T_184_R010_595 defined by calibration of the temperature sensor

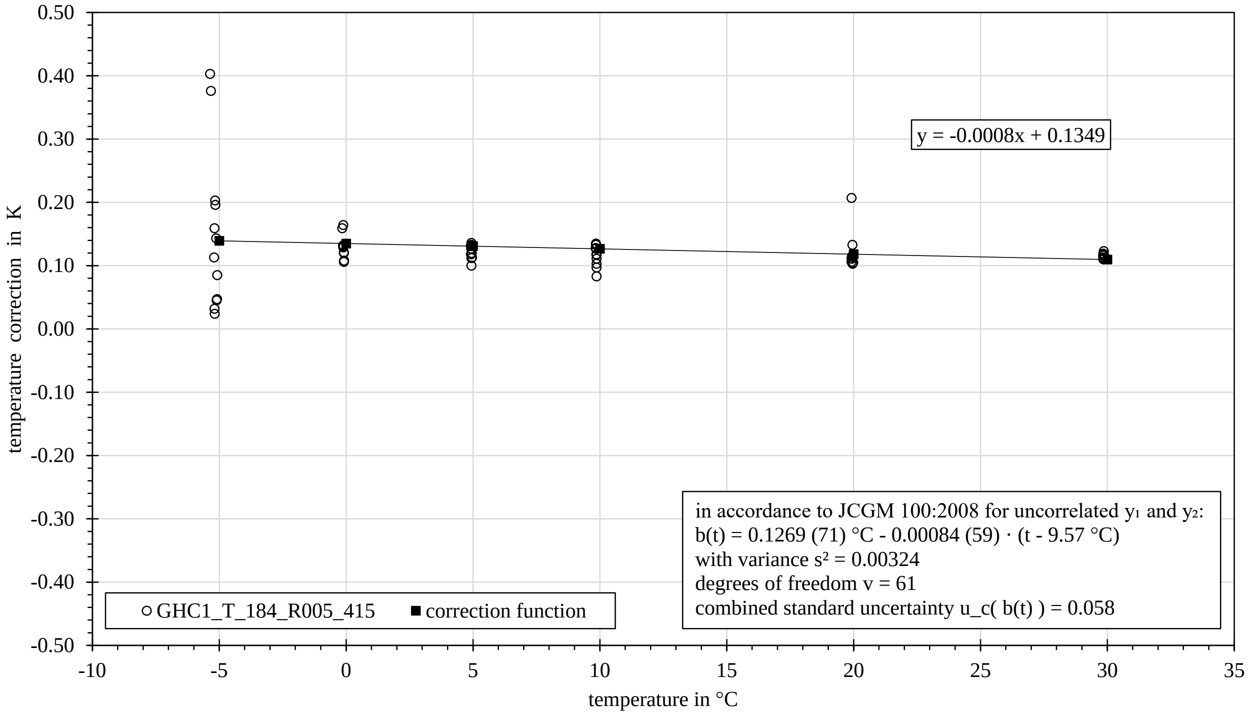

Figure 60: necessary correction of GHC1_T_184_R005_415 defined by calibration of the temperature sensor

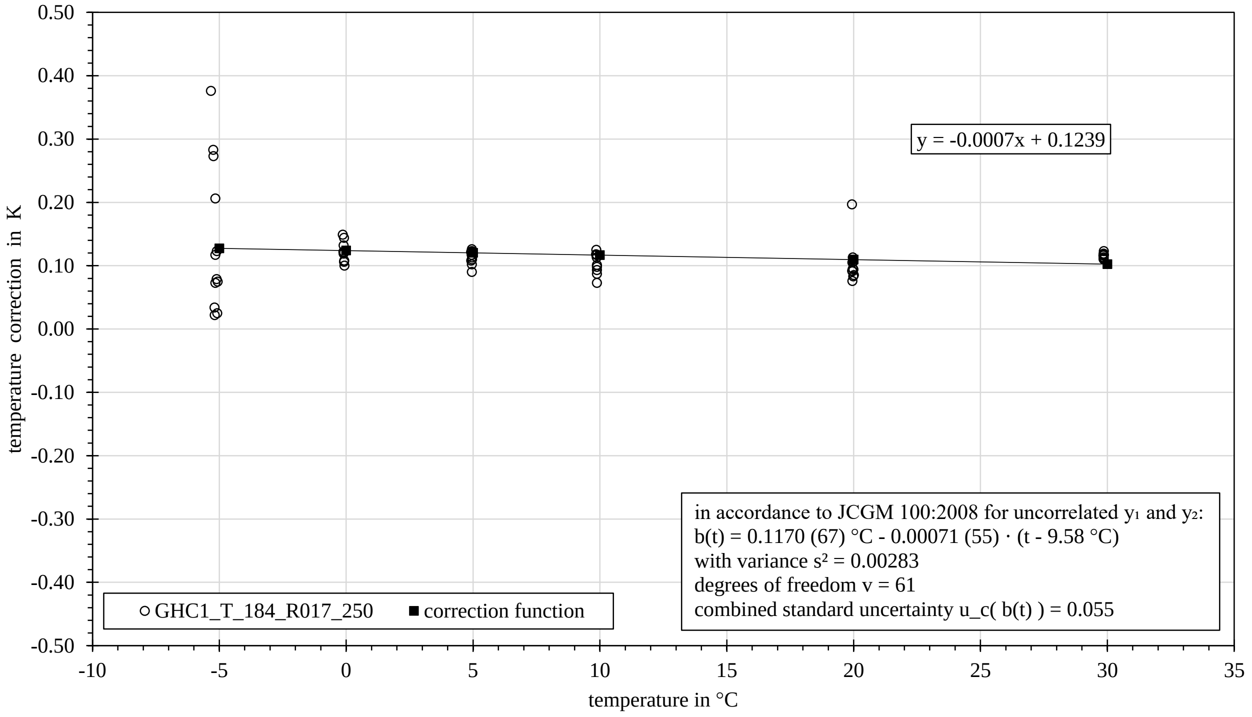

Figure 61: necessary correction of GHC1_T_184_R017_250 defined by calibration of the temperature sensor

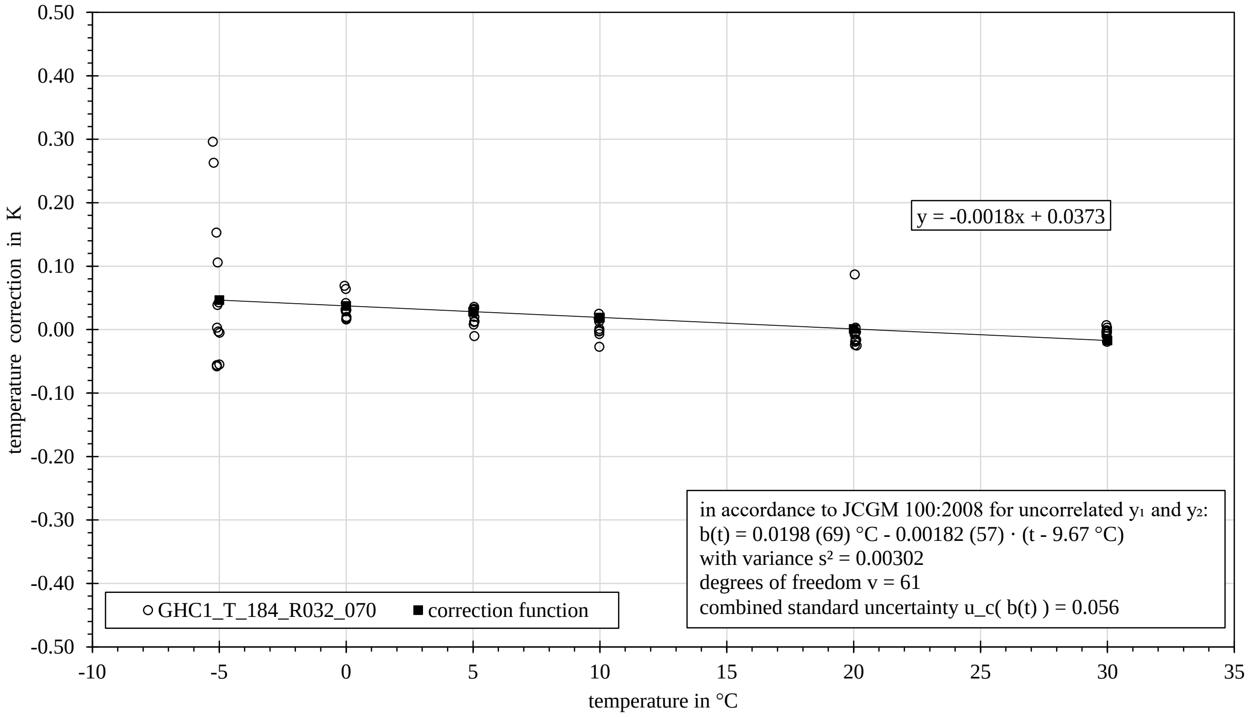

Figure 62: necessary correction of GHC1_T_184_R032_070 defined by calibration of the temperature sensor

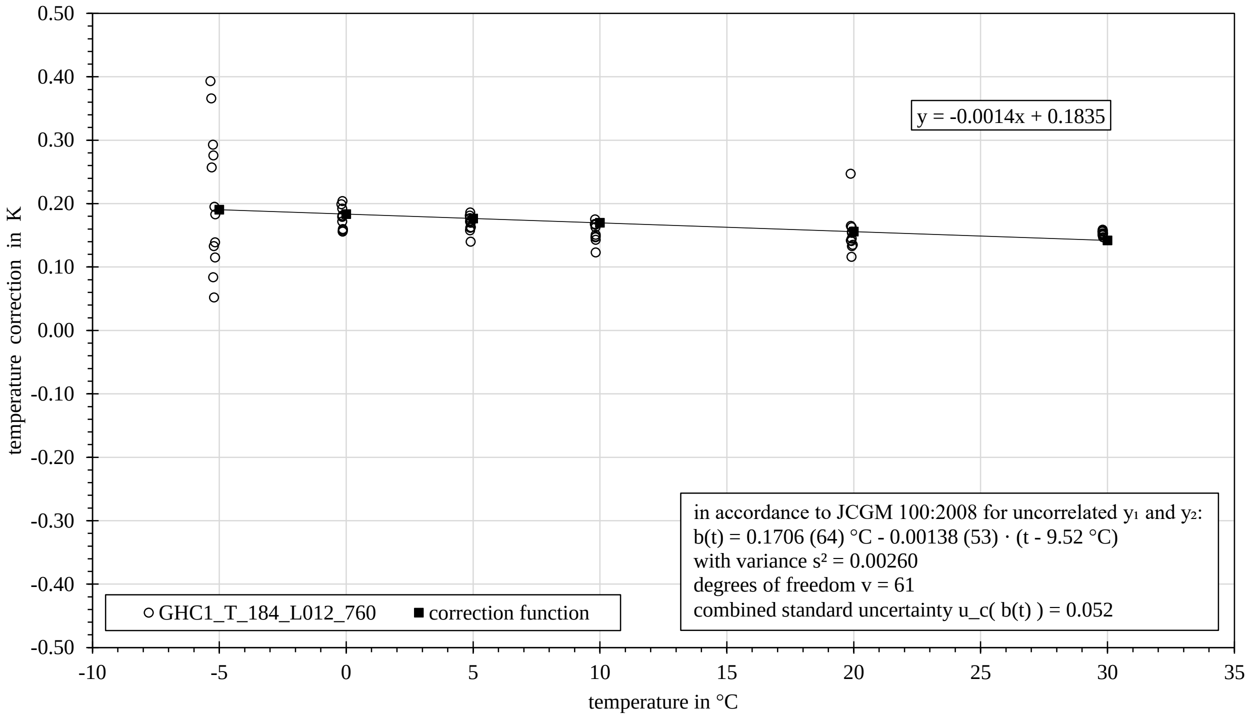

Figure 63: necessary correction of GHC1_T_184_L012_760 defined by calibration of the temperature sensor

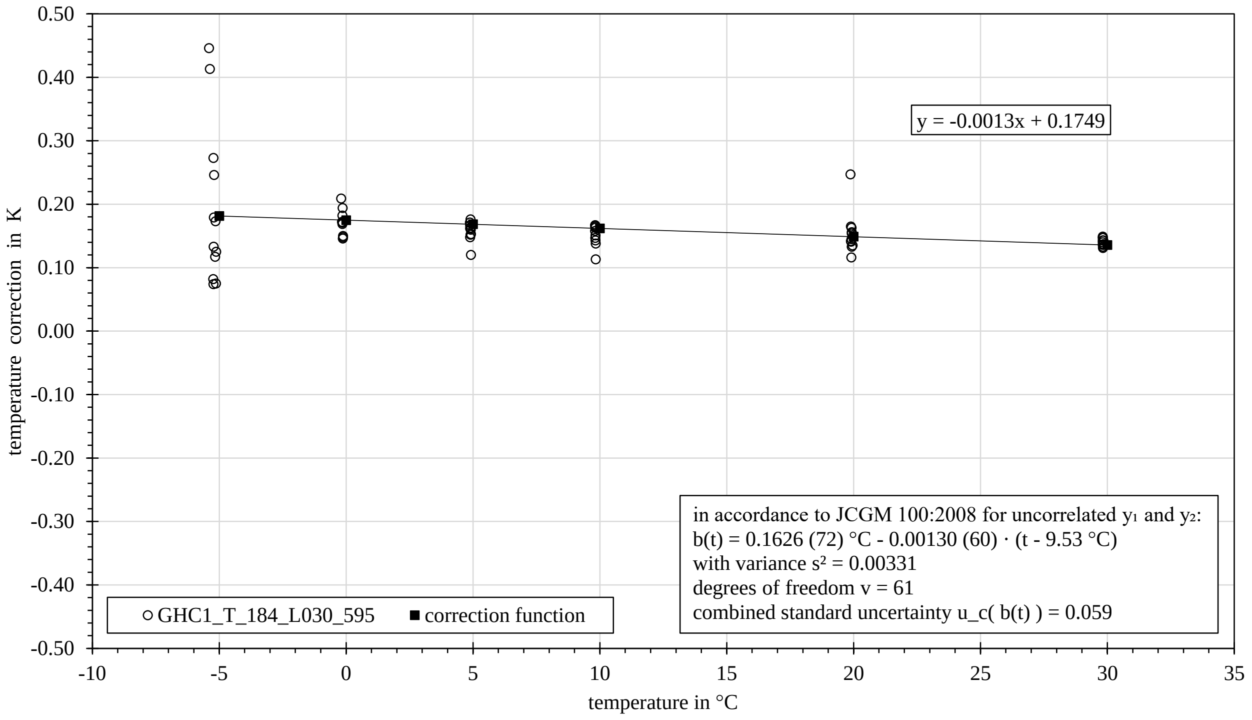

Figure 64: necessary correction of GHC1_T_184_L030_595 defined by calibration of the temperature sensor

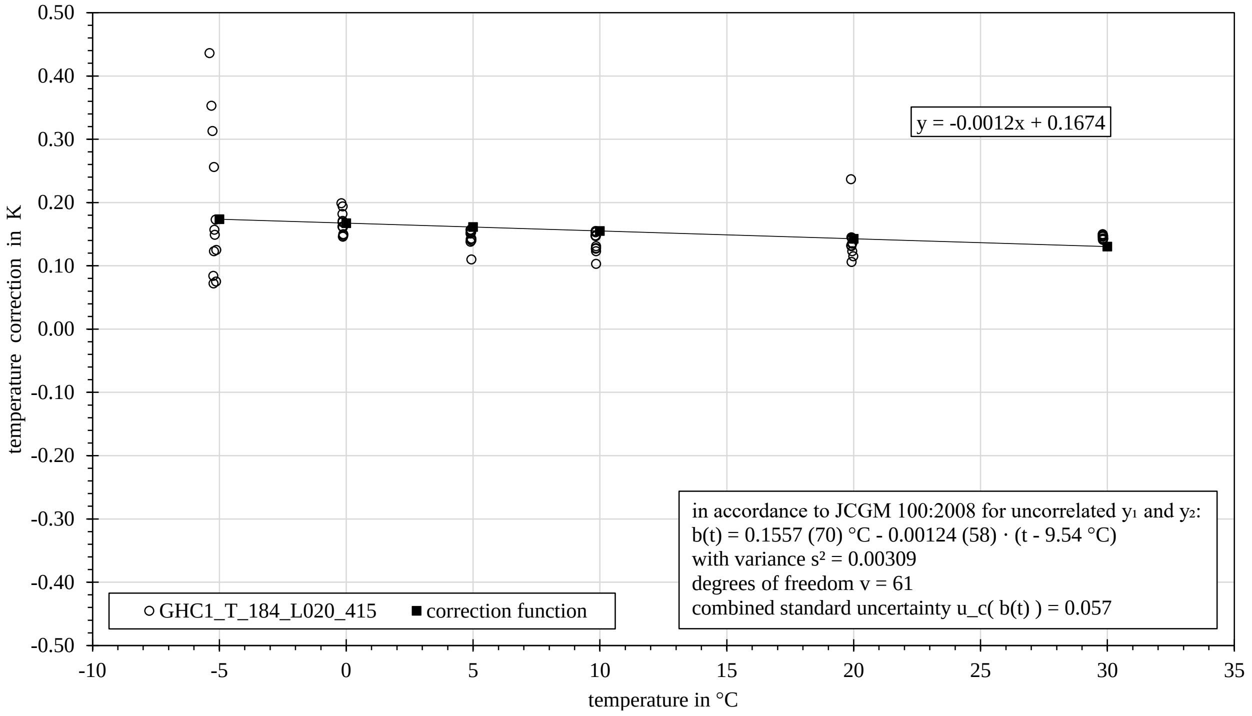

Figure 65: necessary correction of GHC1_T_184_L020_415 defined by calibration of the temperature sensor

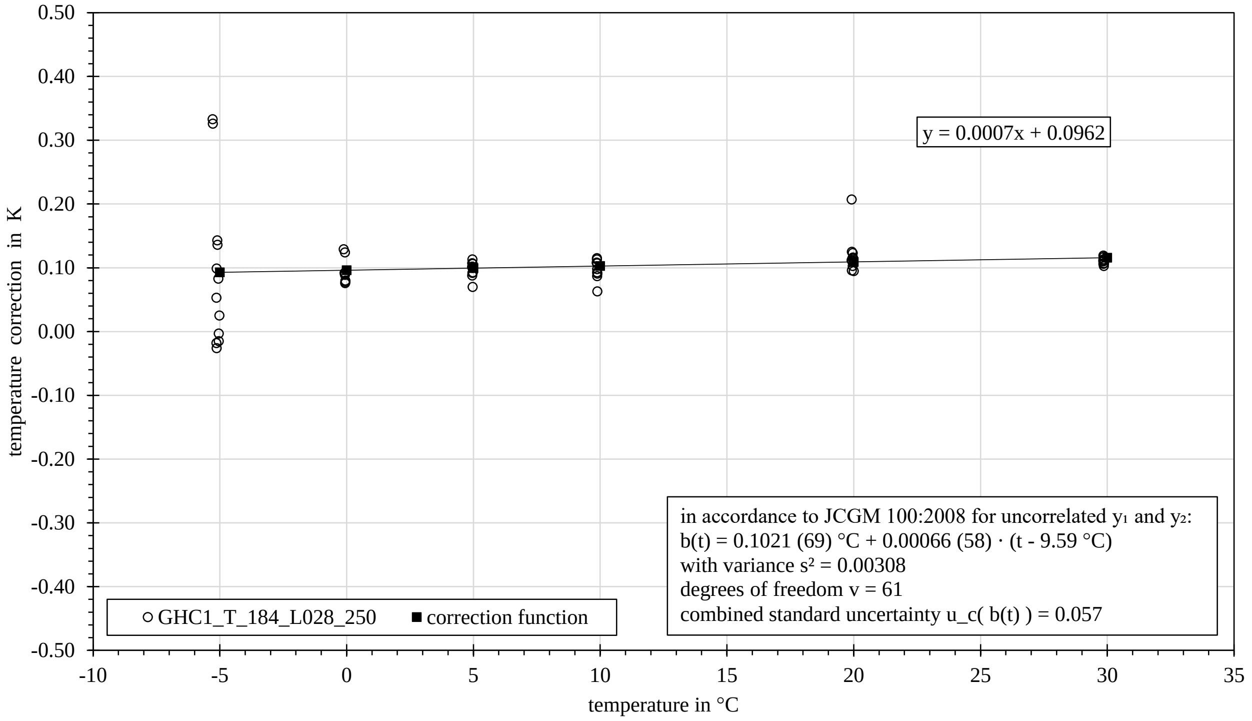

Figure 66: necessary correction of GHC1_T_184_L028_250 defined by calibration of the temperature sensor

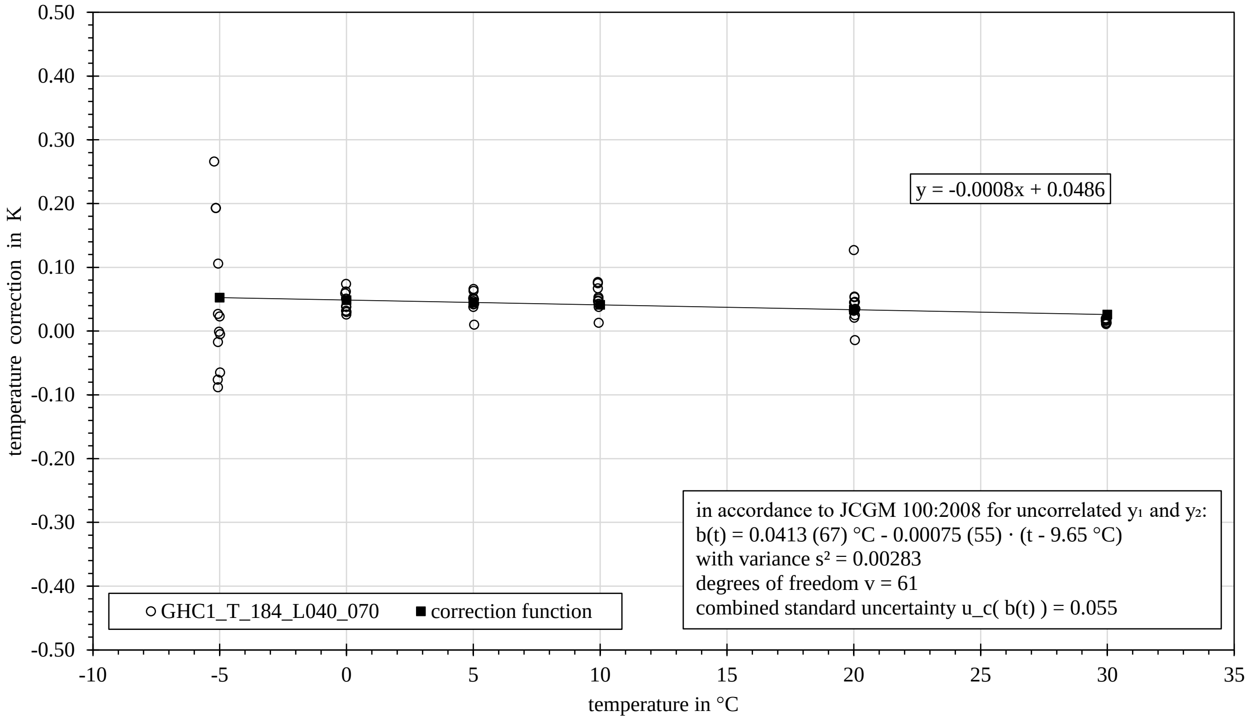

Figure 67: necessary correction of GHC1_T_184_L040_070 defined by calibration of the temperature sensor

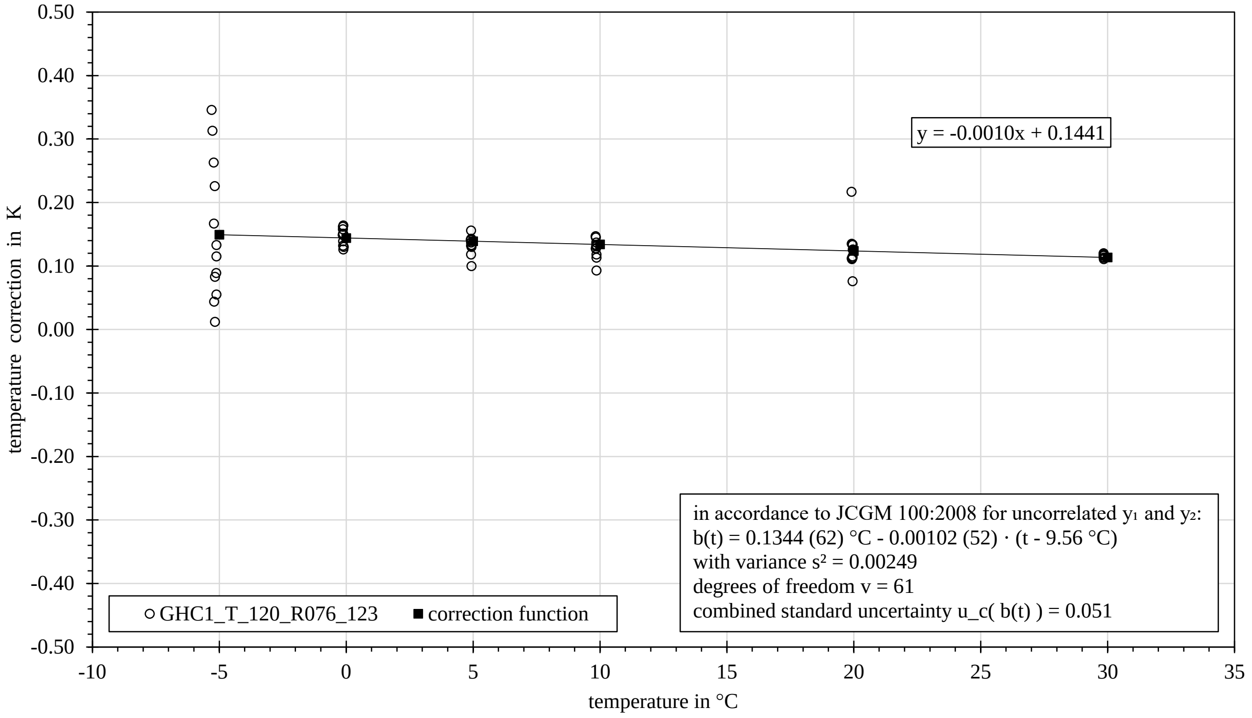

Figure 68: necessary correction of GHC1_T_120_R076_123 defined by calibration of the temperature sensor

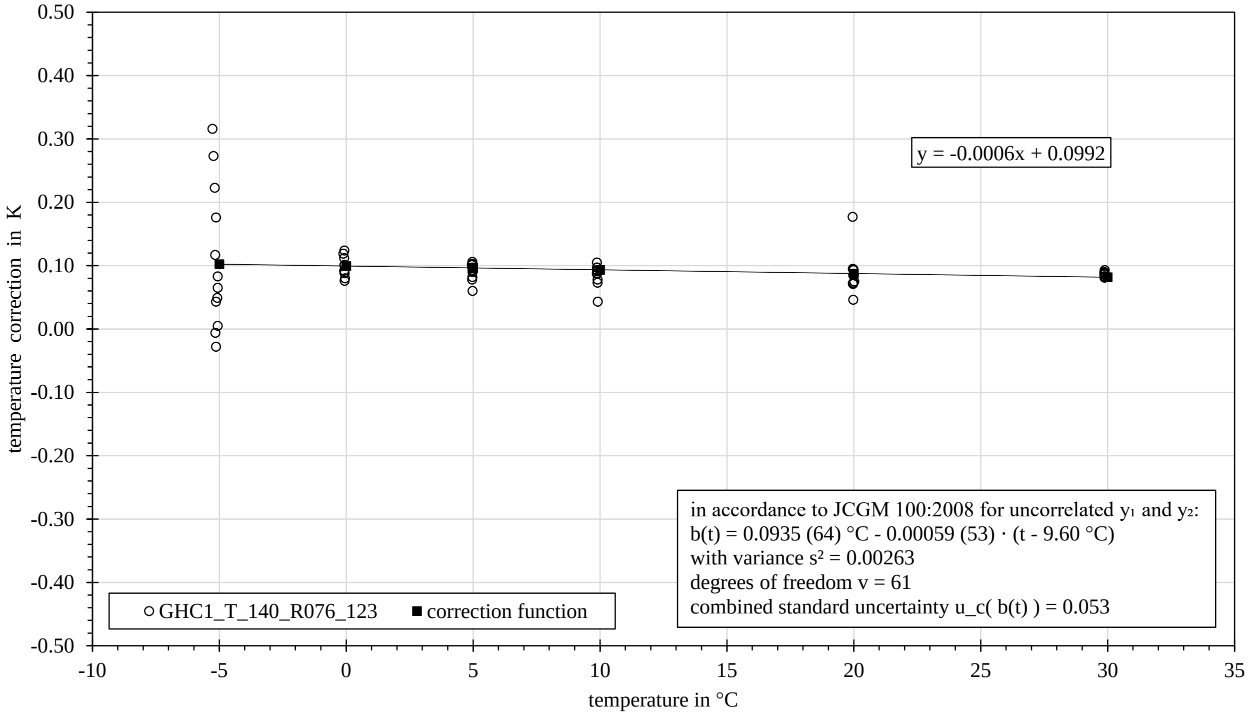

Figure 69: necessary correction of GHC1_T_140_R076_123 defined by calibration of the temperature sensor

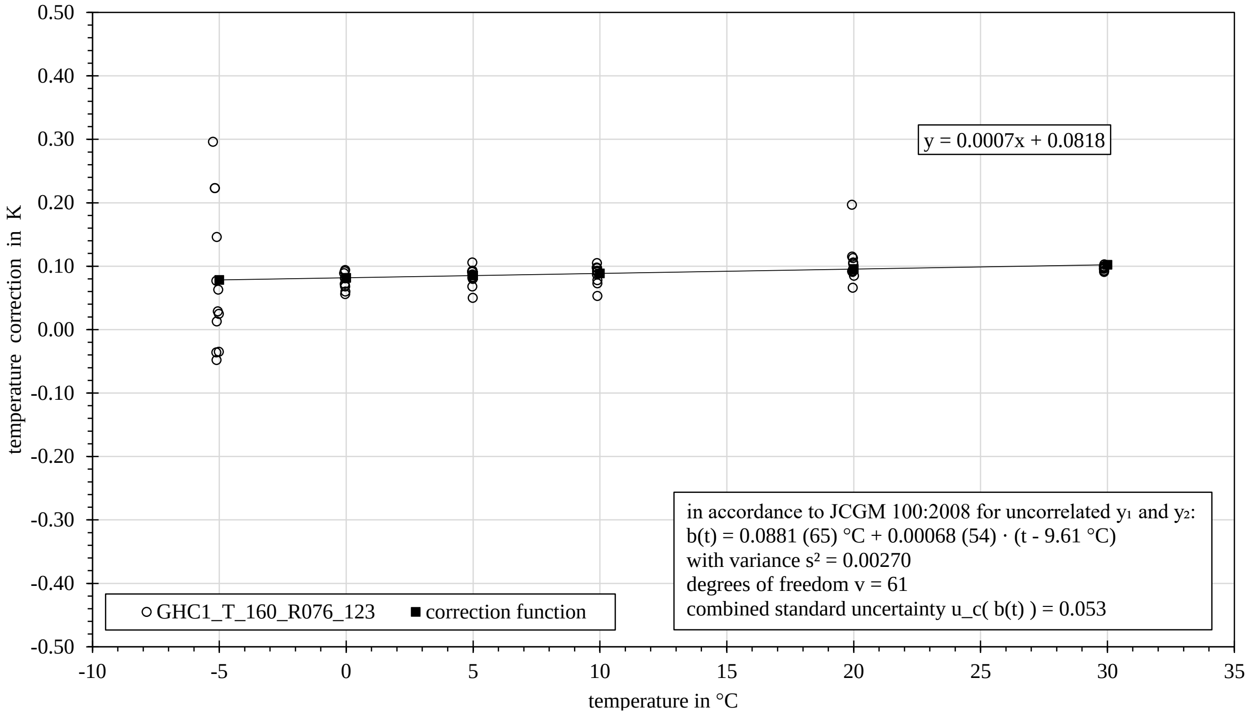

Figure 70: necessary correction of GHC1_T_160_R076_123 defined by calibration of the temperature sensor

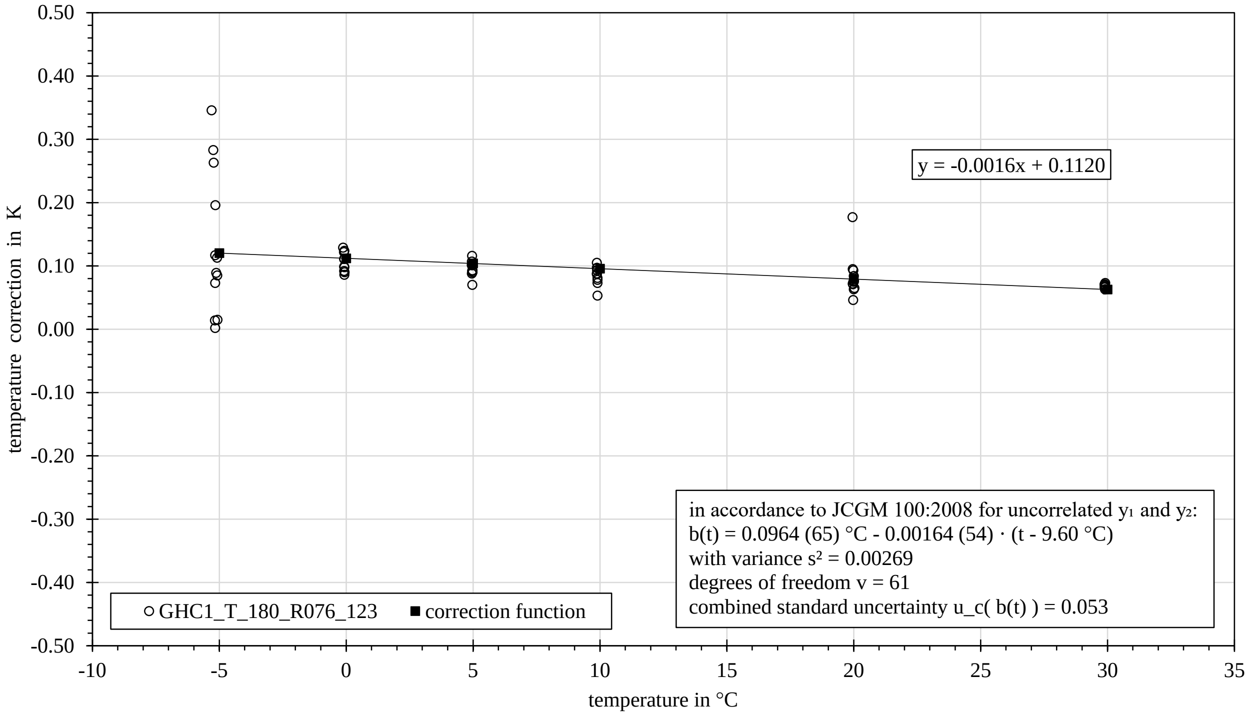

Figure 71: necessary correction of GHC1_T_180_R076_123 defined by calibration of the temperature sensor

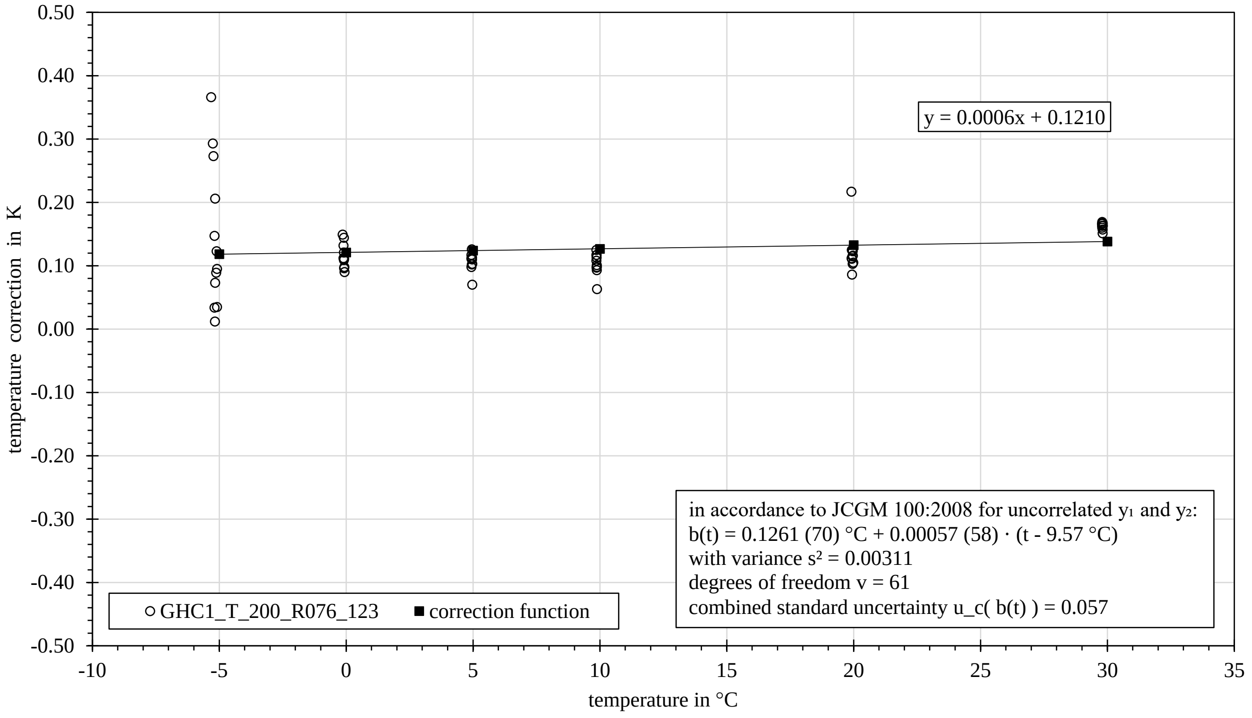

Figure 72: necessary correction of GHC1_T_200_R076_123 defined by calibration of the temperature sensor

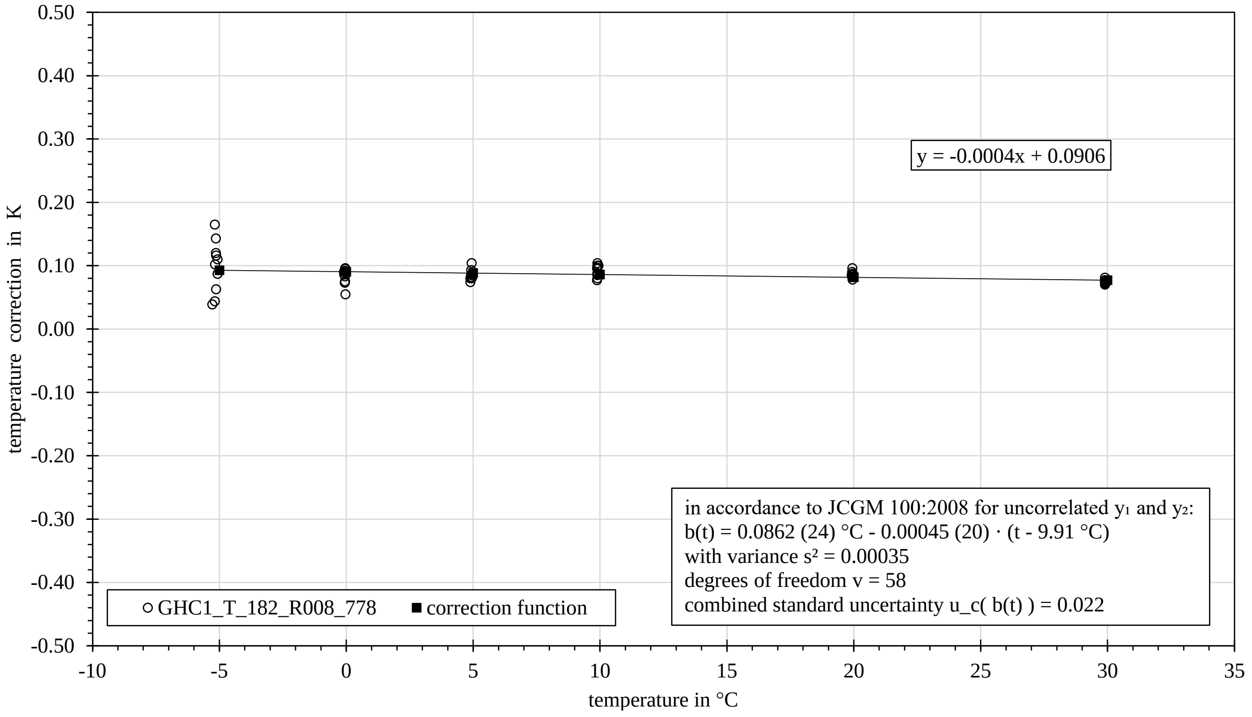

Figure 73: necessary correction of GHC1_T_182_R008_778 defined by calibration of the temperature sensor

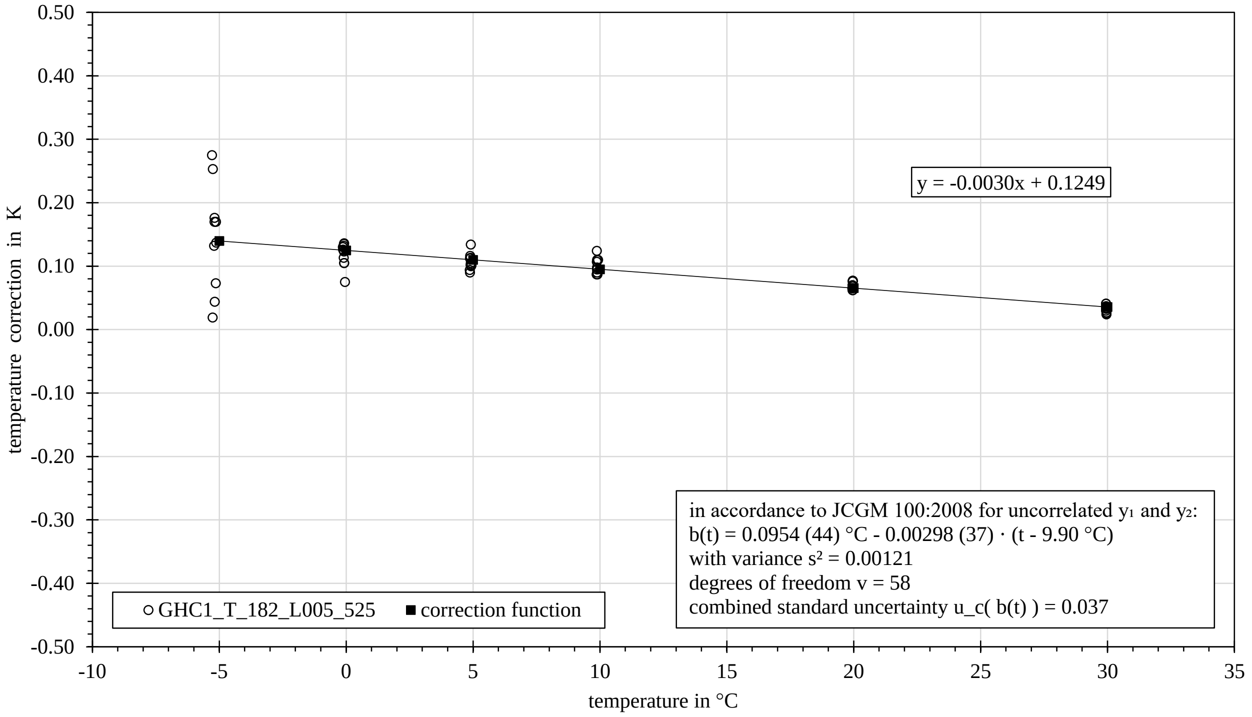

Figure 74: necessary correction of GHC1_T_182_L005_525 defined by calibration of the temperature sensor

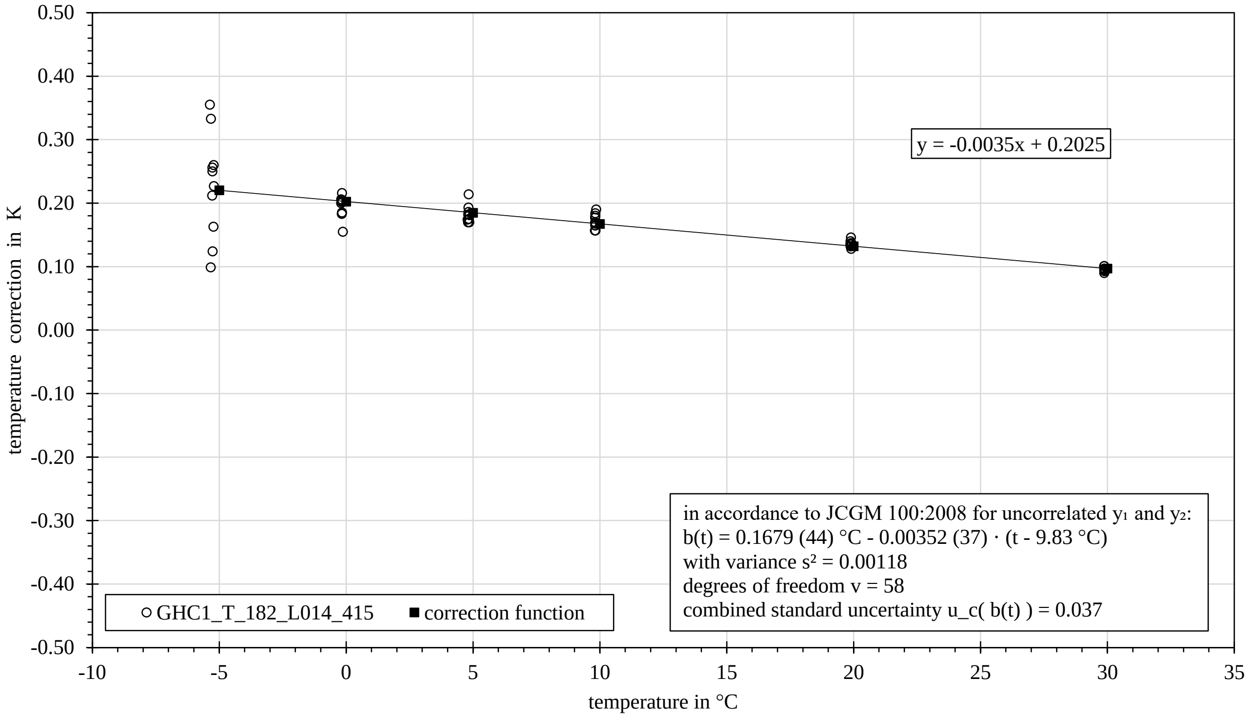

Figure 75: necessary correction of GHC1_T_182_L014_415 defined by calibration of the temperature sensor

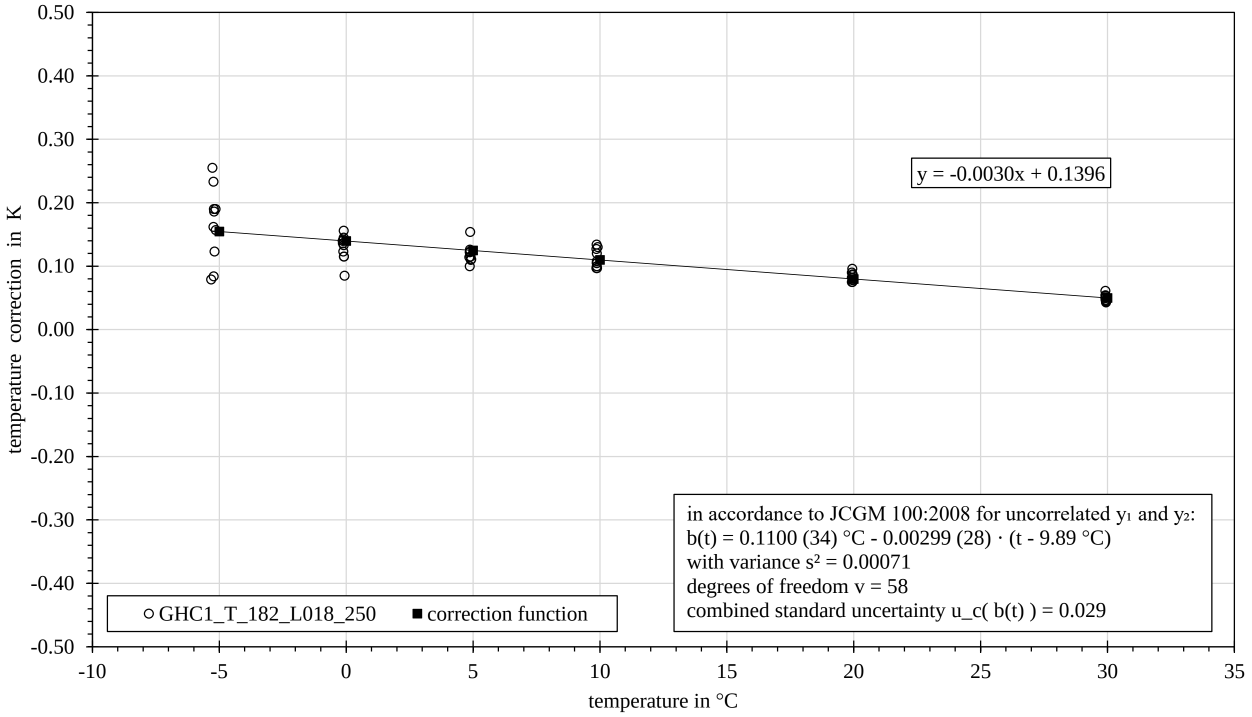

Figure 76: necessary correction of GHC1_T_182_L018_250 defined by calibration of the temperature sensor

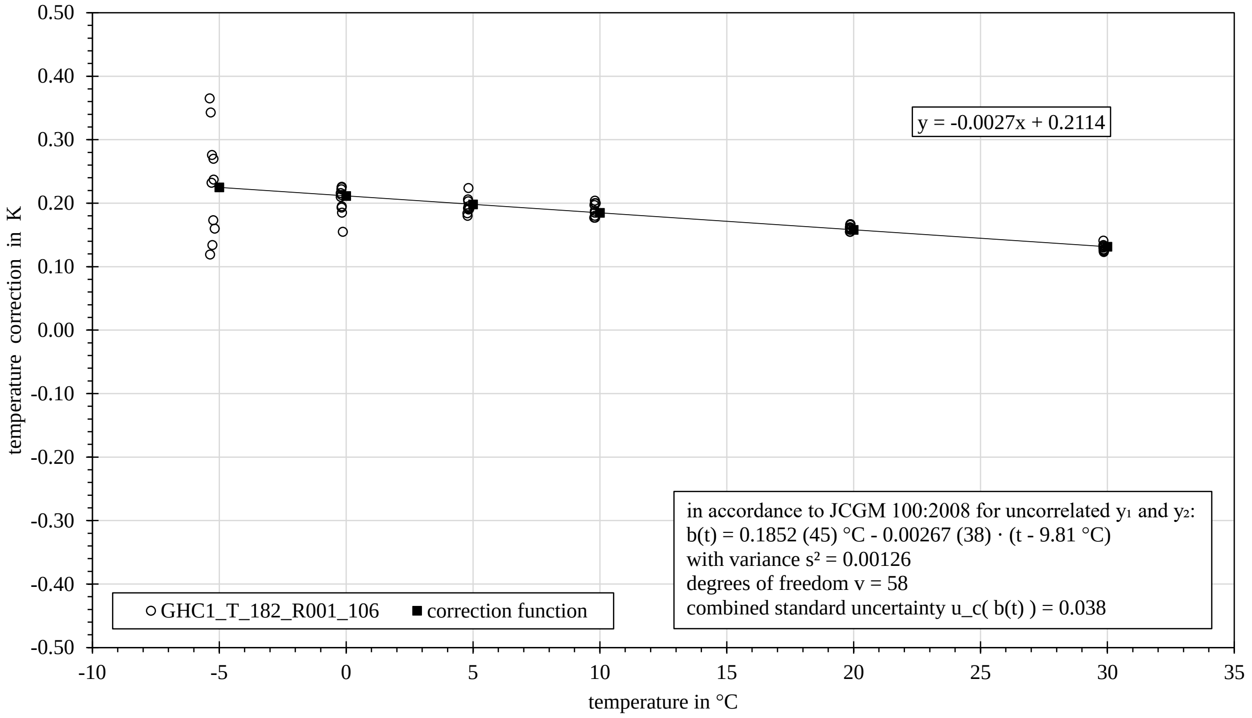

Figure 77: necessary correction of GHC1_T_182_R001_106 defined by calibration of the temperature sensor

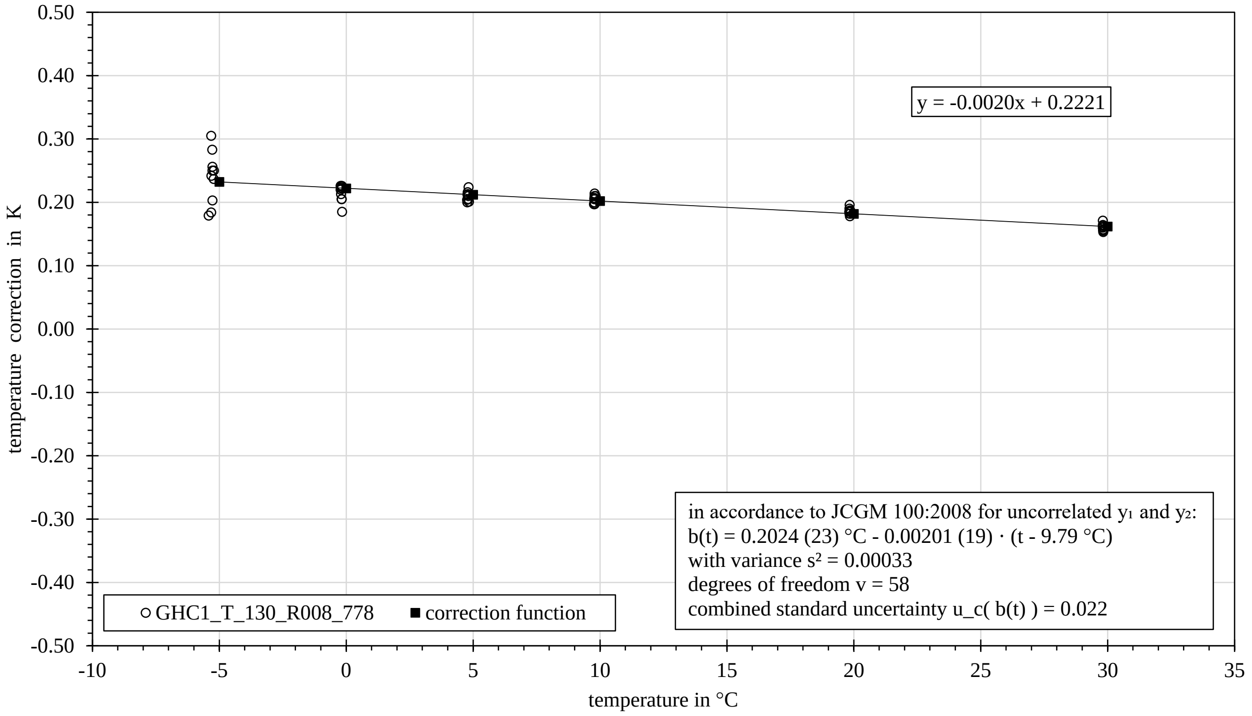

Figure 78: necessary correction of GHC1_T_130_R008_778 defined by calibration of the temperature sensor

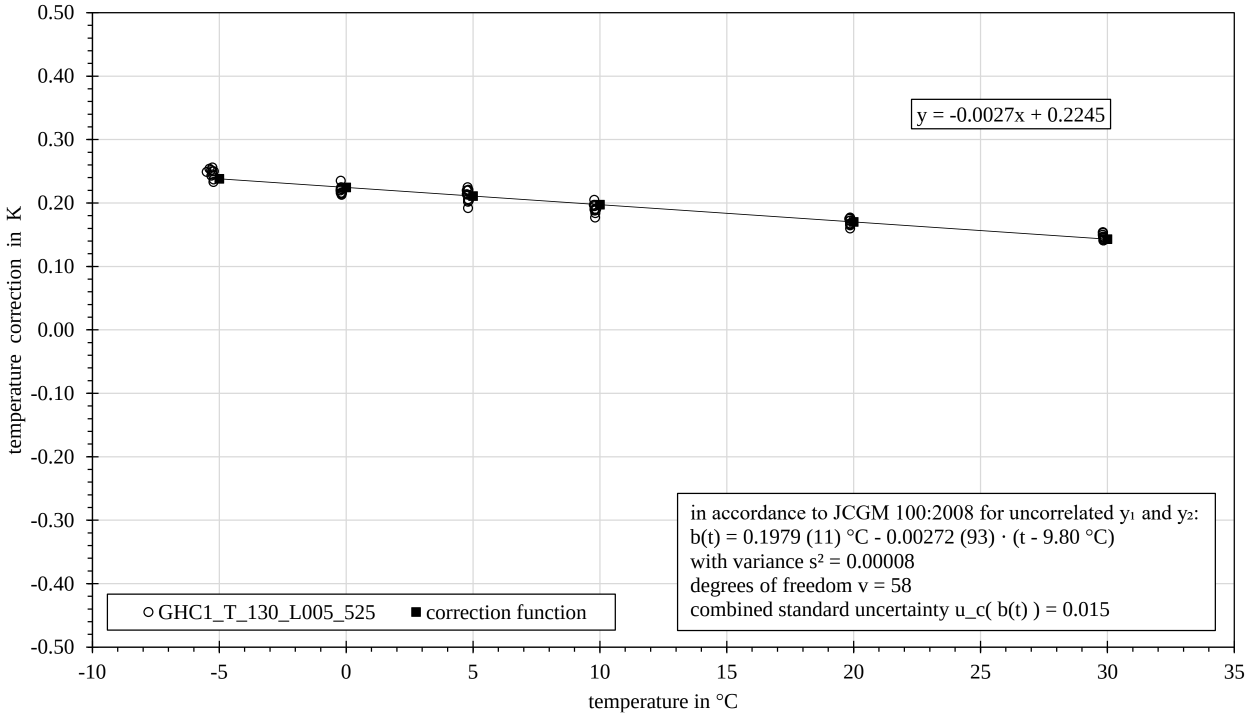

Figure 79: necessary correction of GHC1_T_130_L005_525 defined by calibration of the temperature sensor

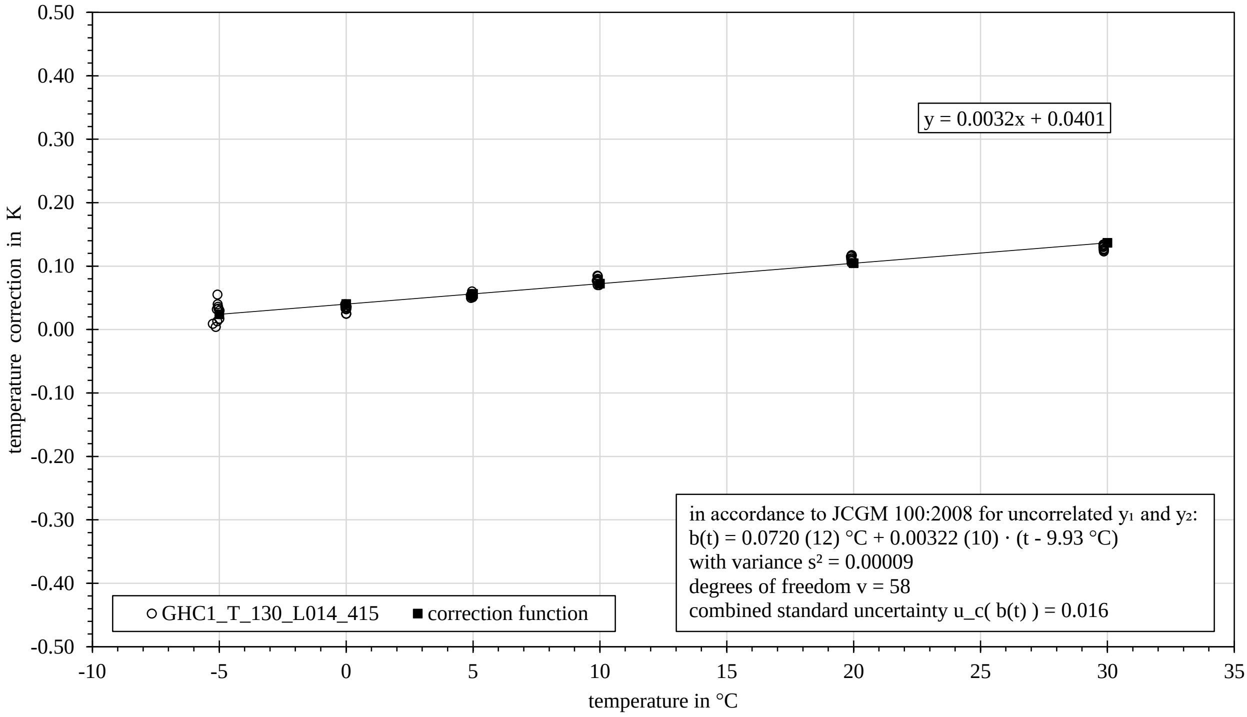

Figure 80: necessary correction of GHC1_T_130_L014_415 defined by calibration of the temperature sensor

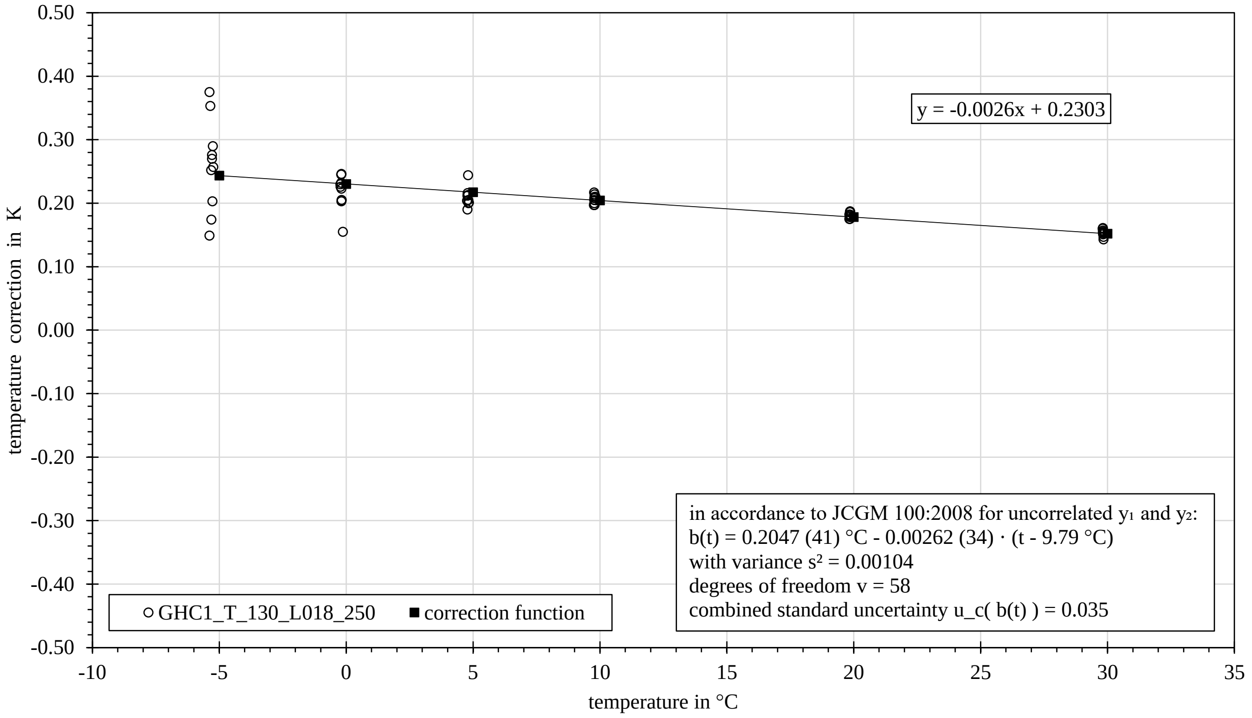

Figure 81: necessary correction of GHC1_T_130_L018_250 defined by calibration of the temperature sensor

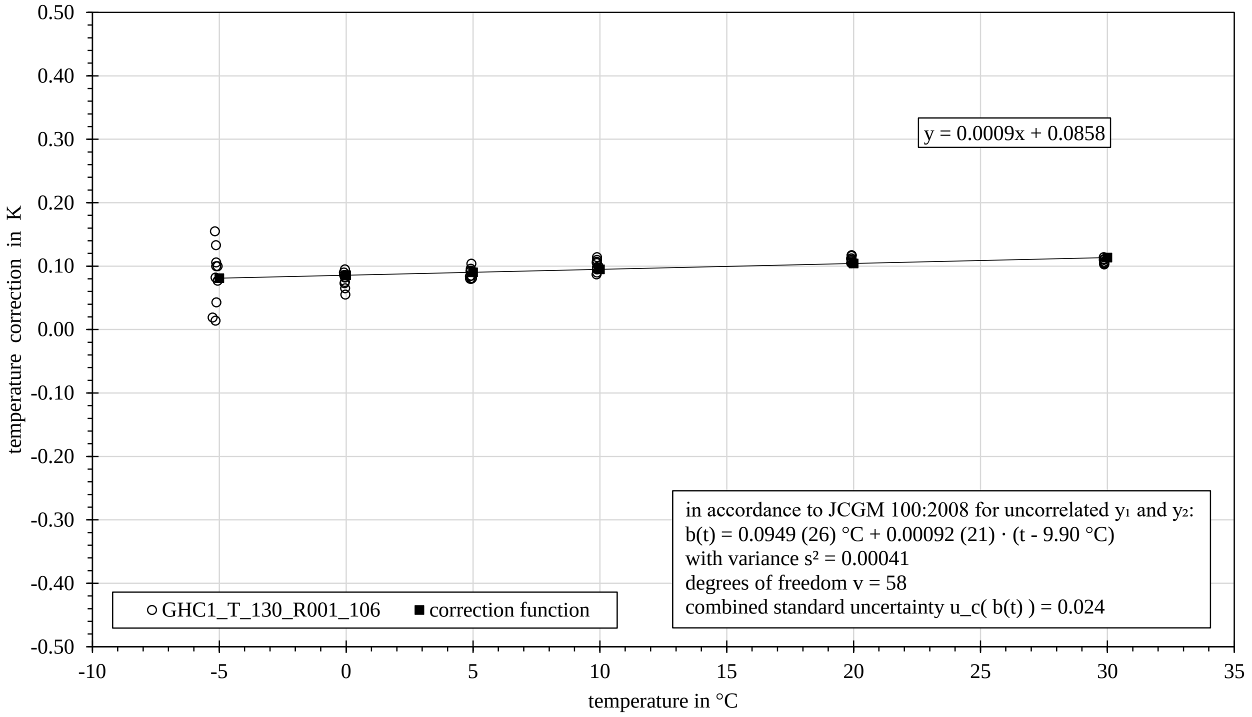

Figure 82: necessary correction of GHC1_T_130_R001_106 defined by calibration of the temperature sensor

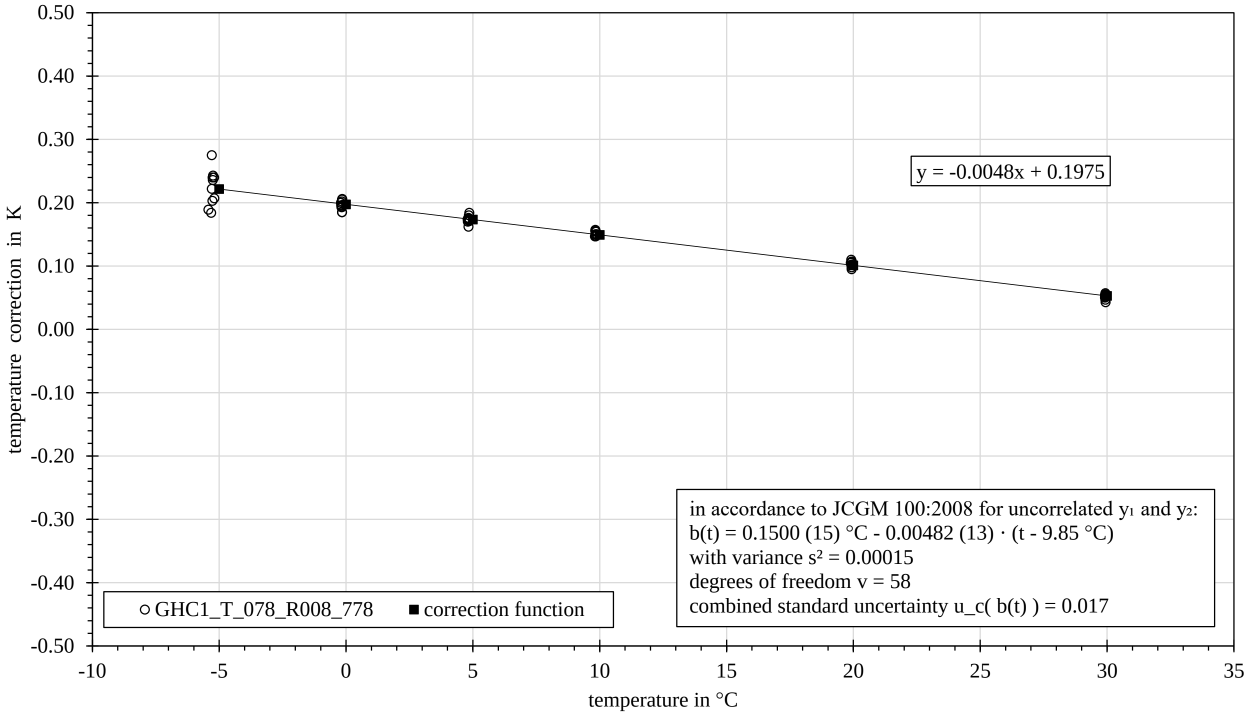

Figure 83: necessary correction of GHC1_T_078_R008_778 defined by calibration of the temperature sensor

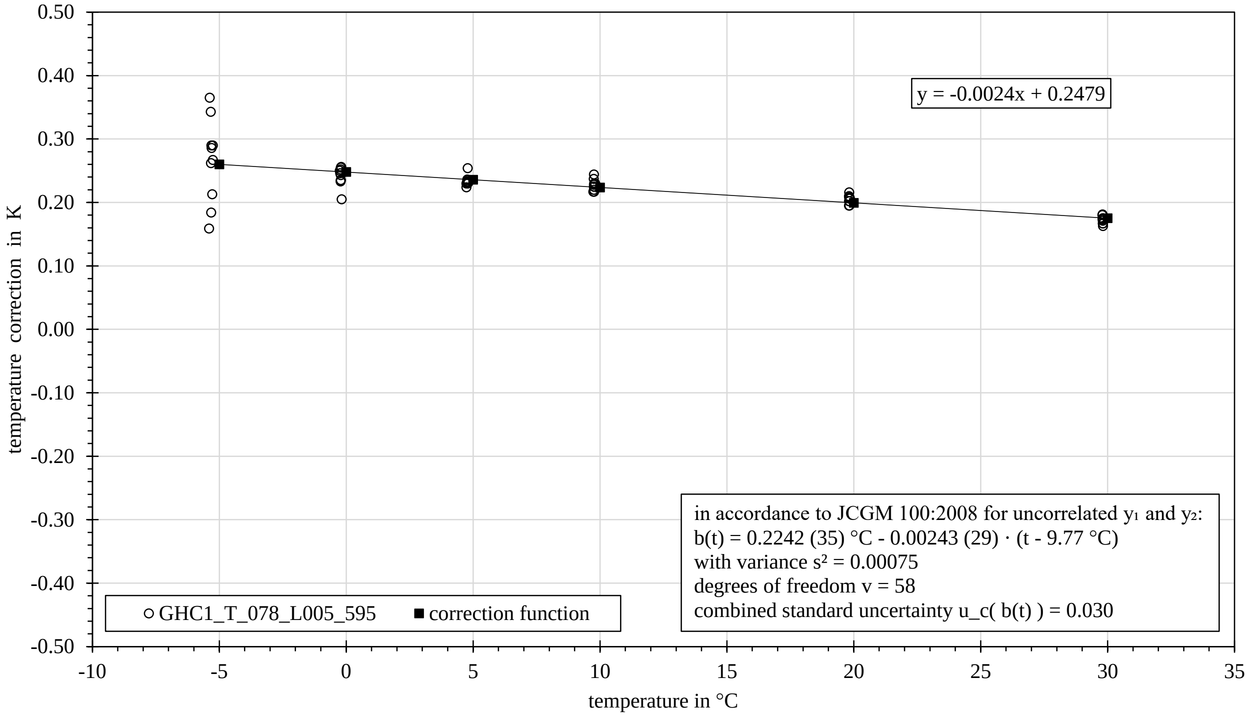

Figure 84: necessary correction of GHC1_T_078_L005_595 defined by calibration of the temperature sensor

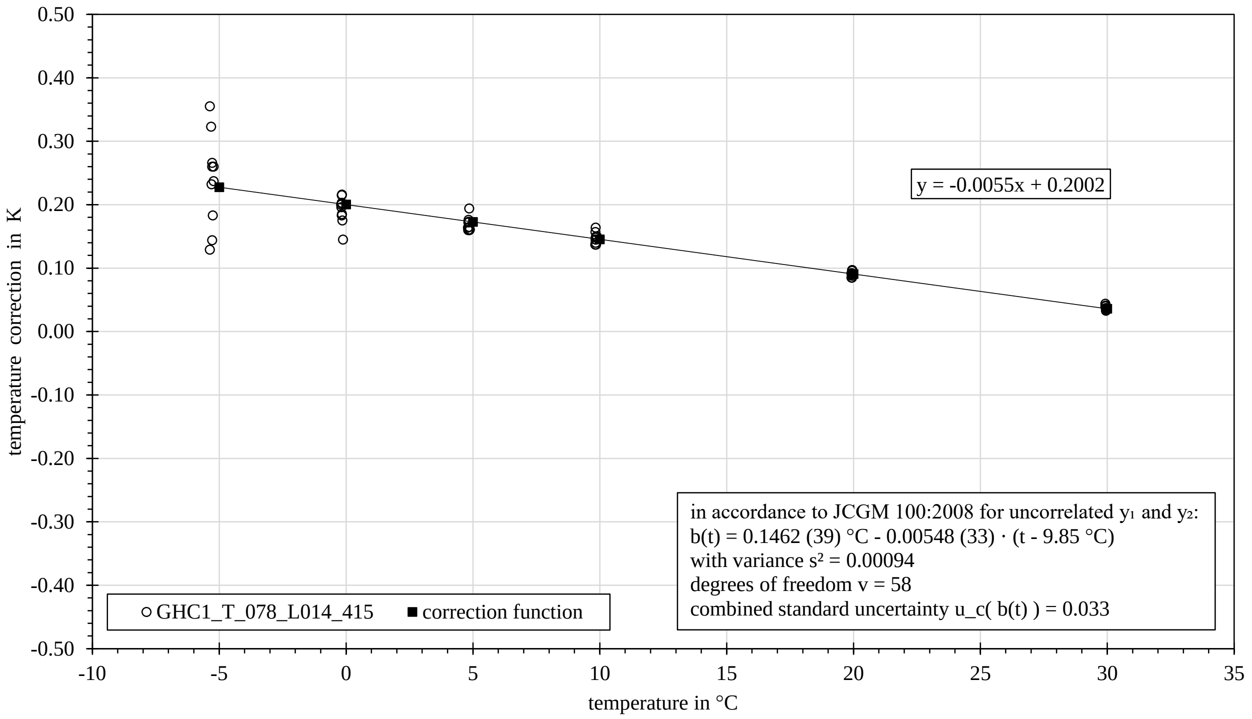

Figure 85: necessary correction of GHC1_T_078_L014_415 defined by calibration of the temperature sensor

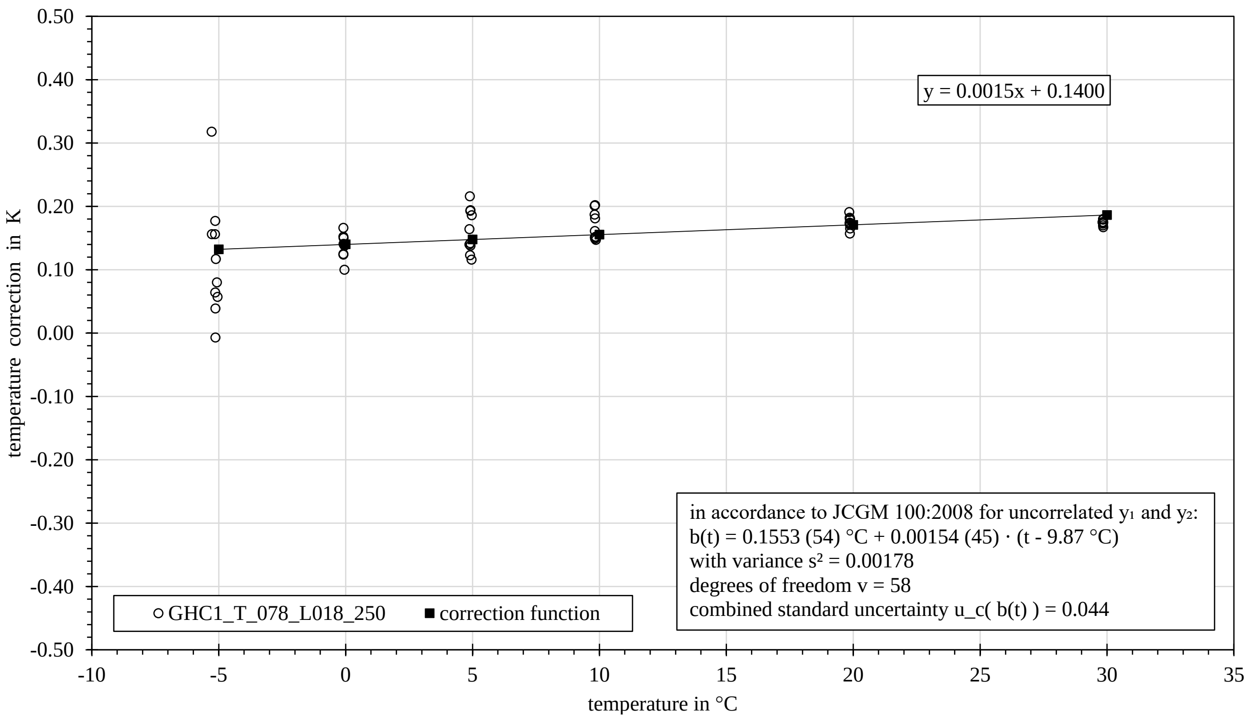

Figure 86: necessary correction of GHC1_T_078_L018_250 defined by calibration of the temperature sensor

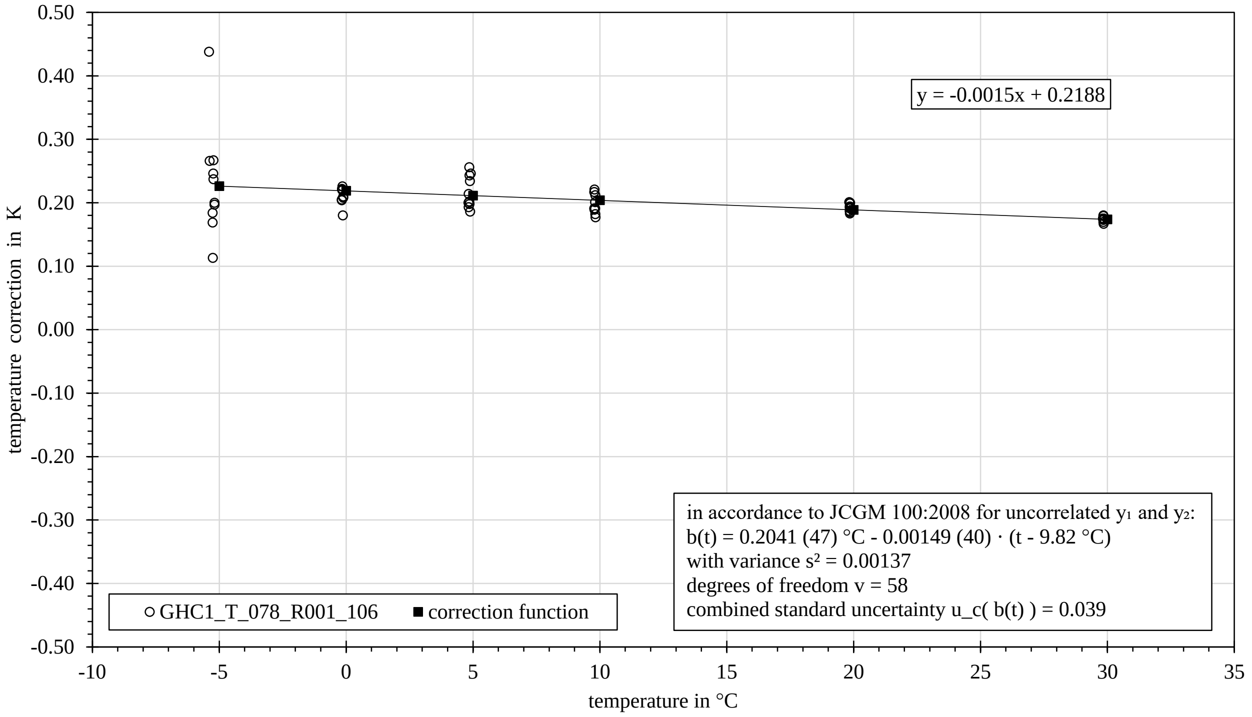

Figure 87: necessary correction of GHC1_T_078_R001_106 defined by calibration of the temperature sensor

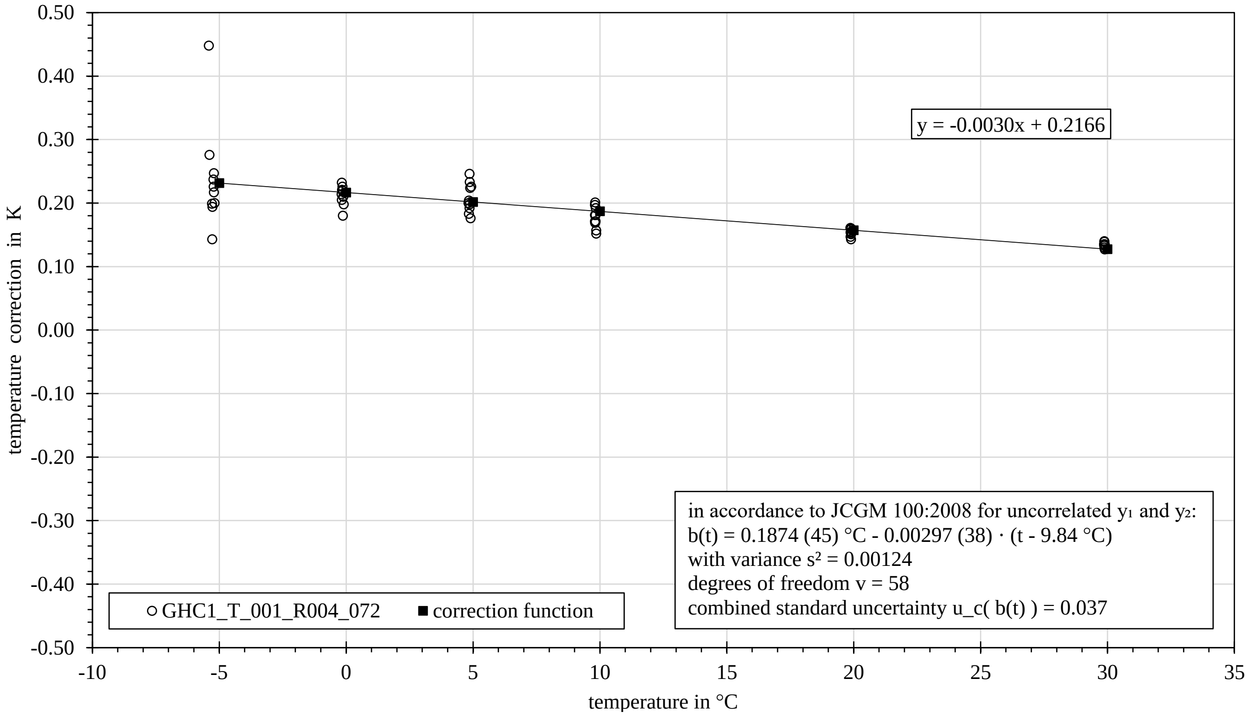

Figure 88: necessary correction of GHC1_T_001_R004_072 defined by calibration of the temperature sensor

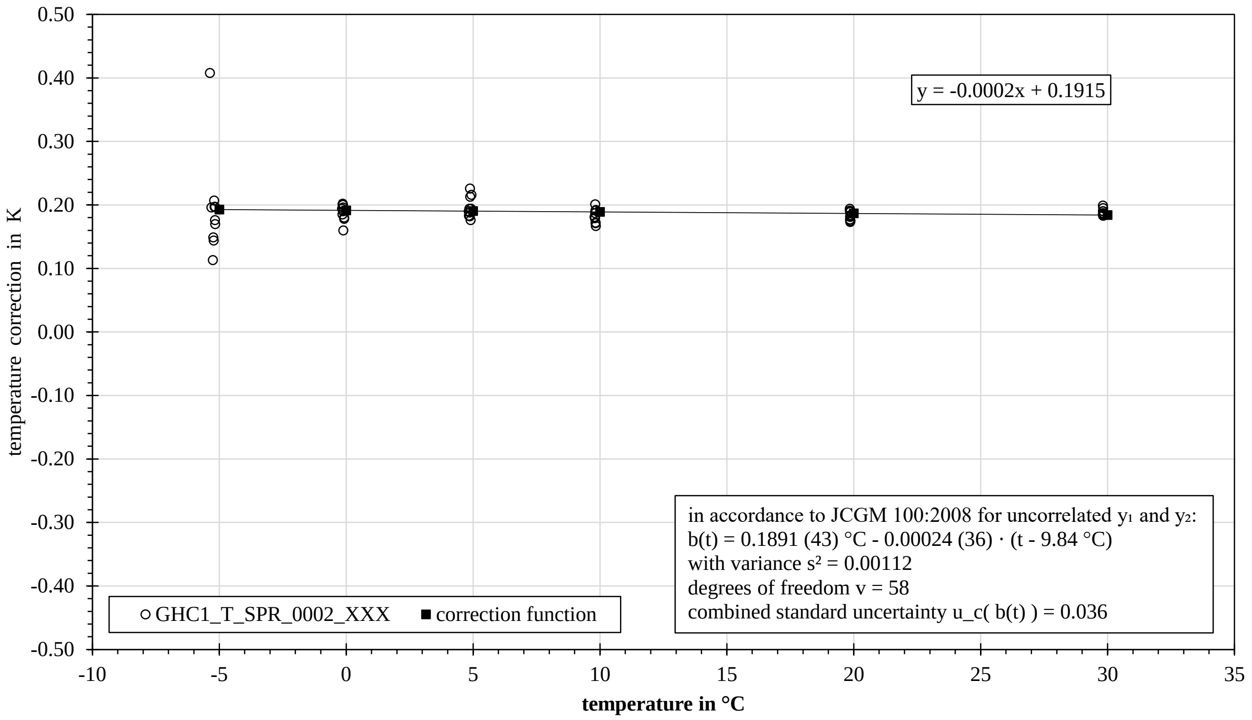

Figure 89: necessary correction of GHC1_T_SPR_0002_XXX defined by calibration of the temperature sensor

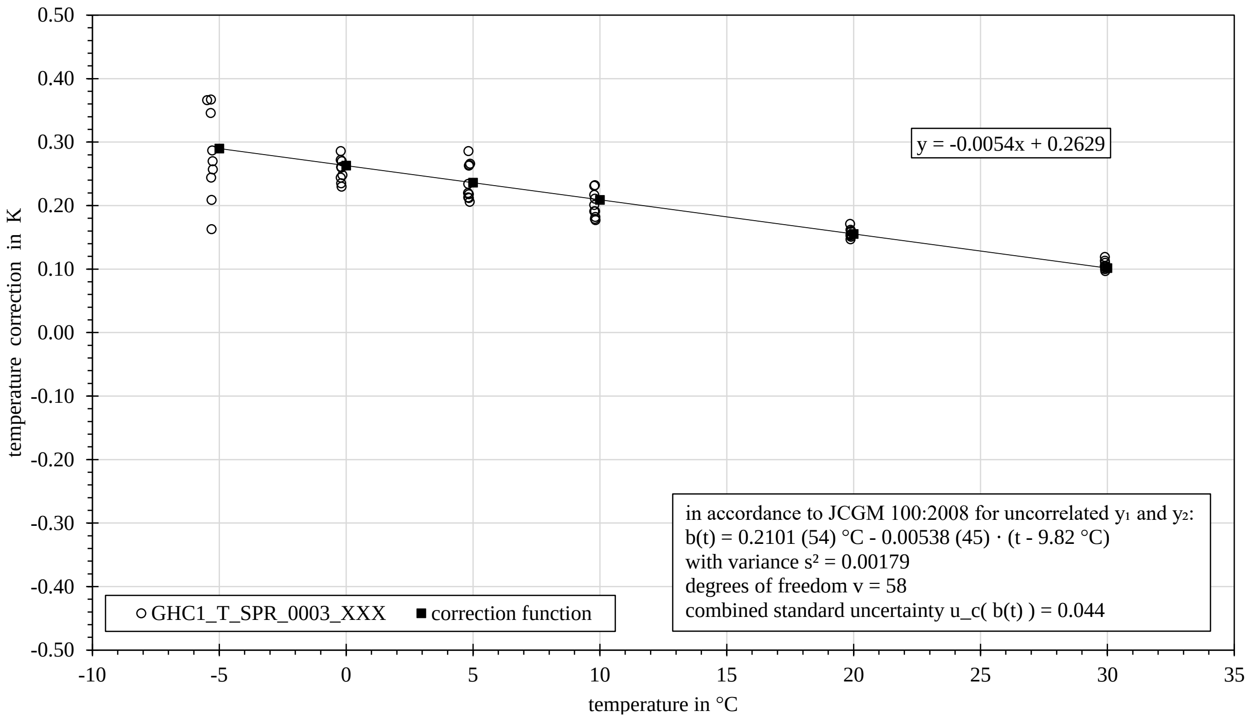

Figure 90: necessary correction of GHC1_T_SPR_0003_XXX defined by calibration of the temperature sensor

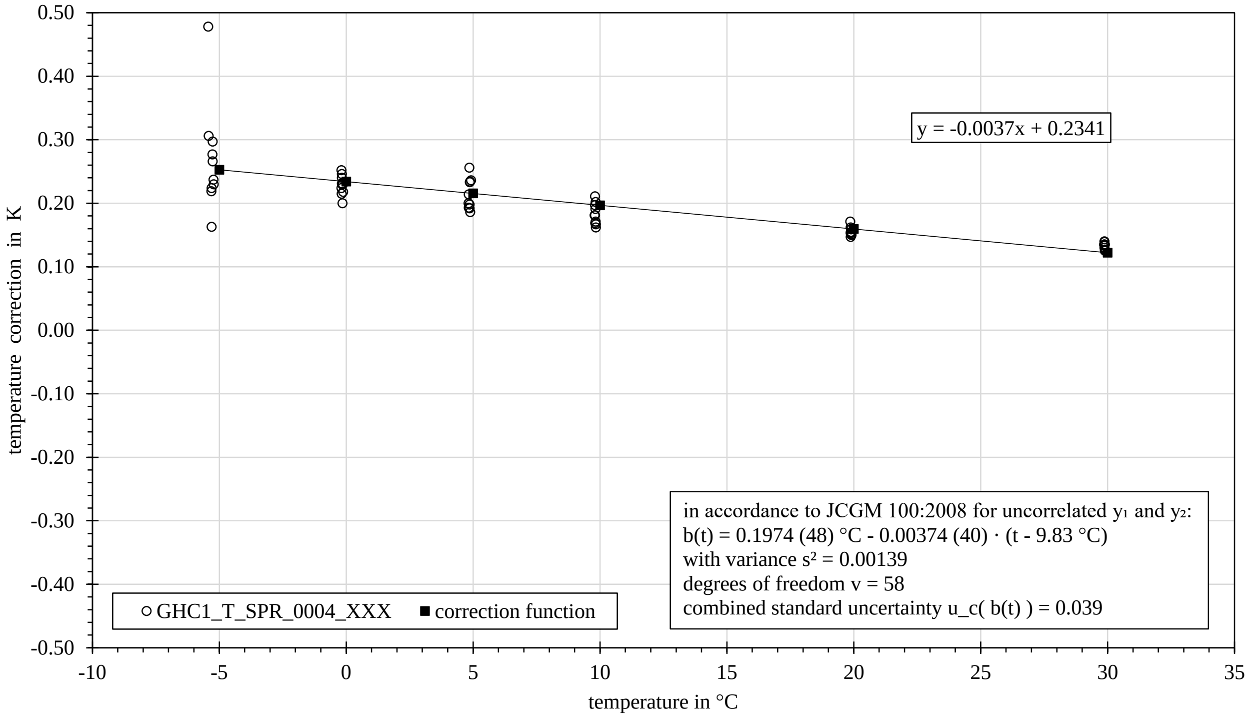

Figure 91: necessary correction of GHC1_T_SPR_0004_XXX defined by calibration of the temperature sensor

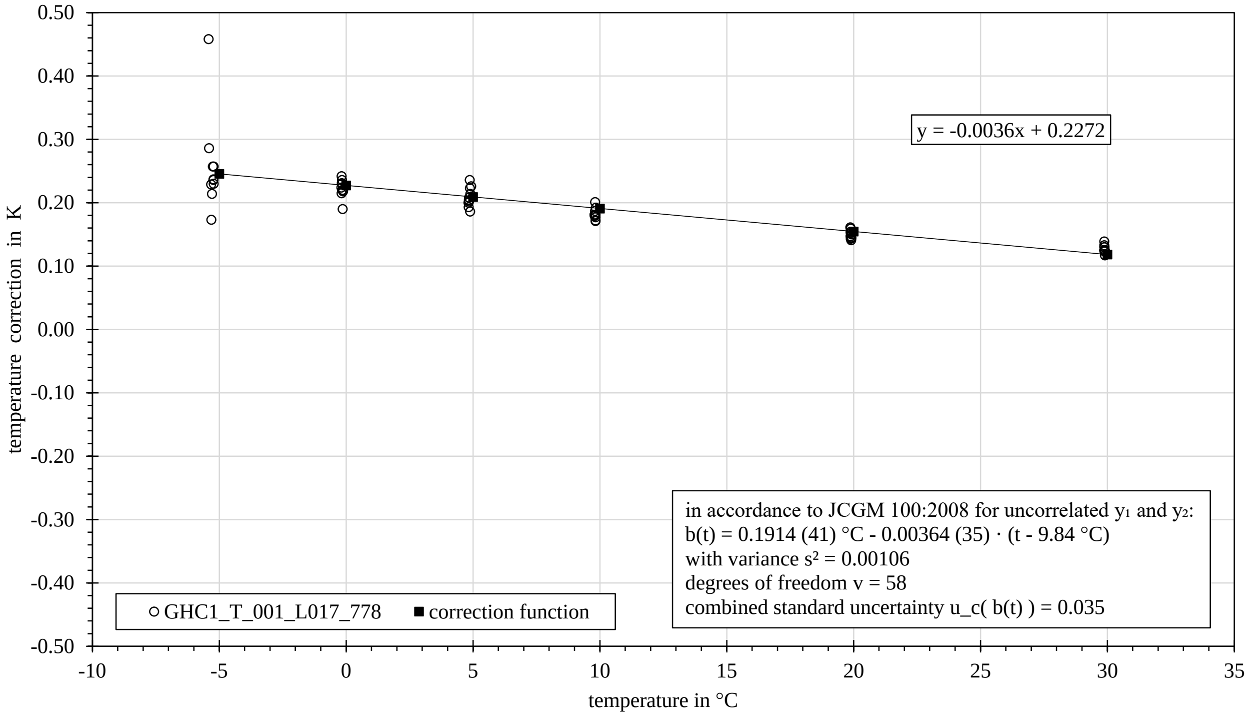

Figure 92: necessary correction of GHC1_T_001_L017_778 defined by calibration of the temperature sensor

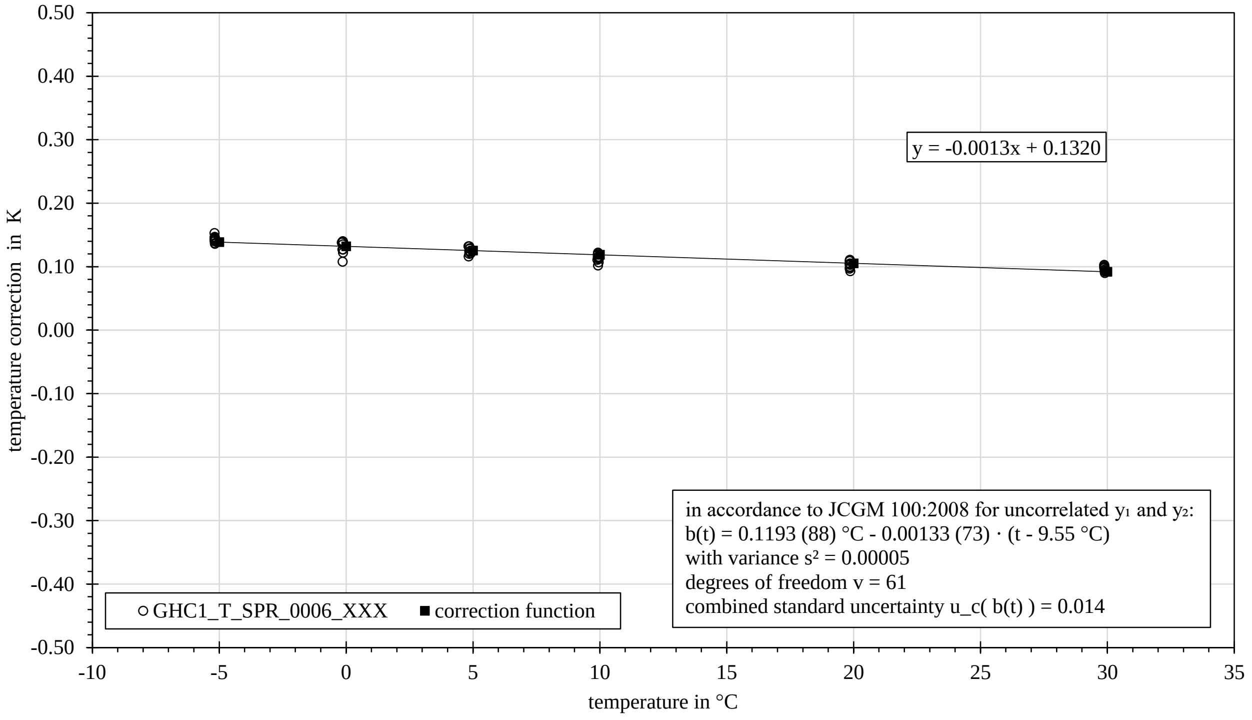

Figure 93: necessary correction of GHC1_T_SPR_0006_XXX defined by calibration of the temperature sensor

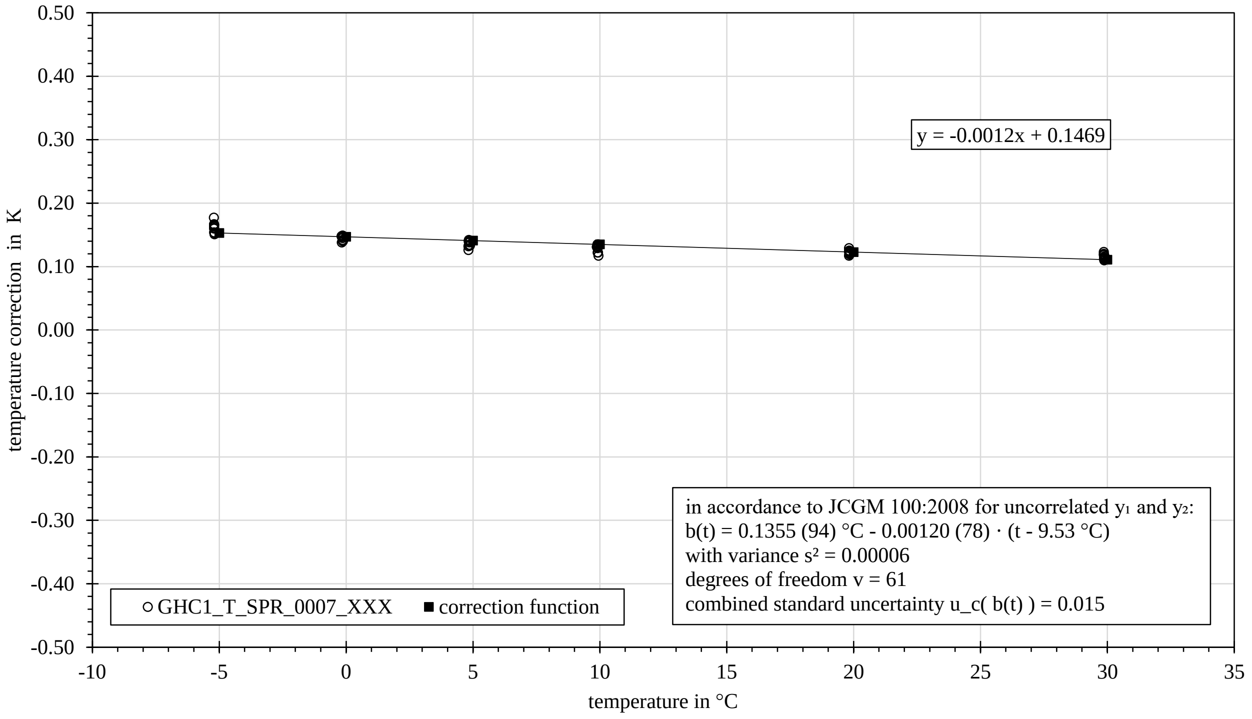

Figure 94: necessary correction of GHC1_T_SPR_0007_XXX defined by calibration of the temperature sensor

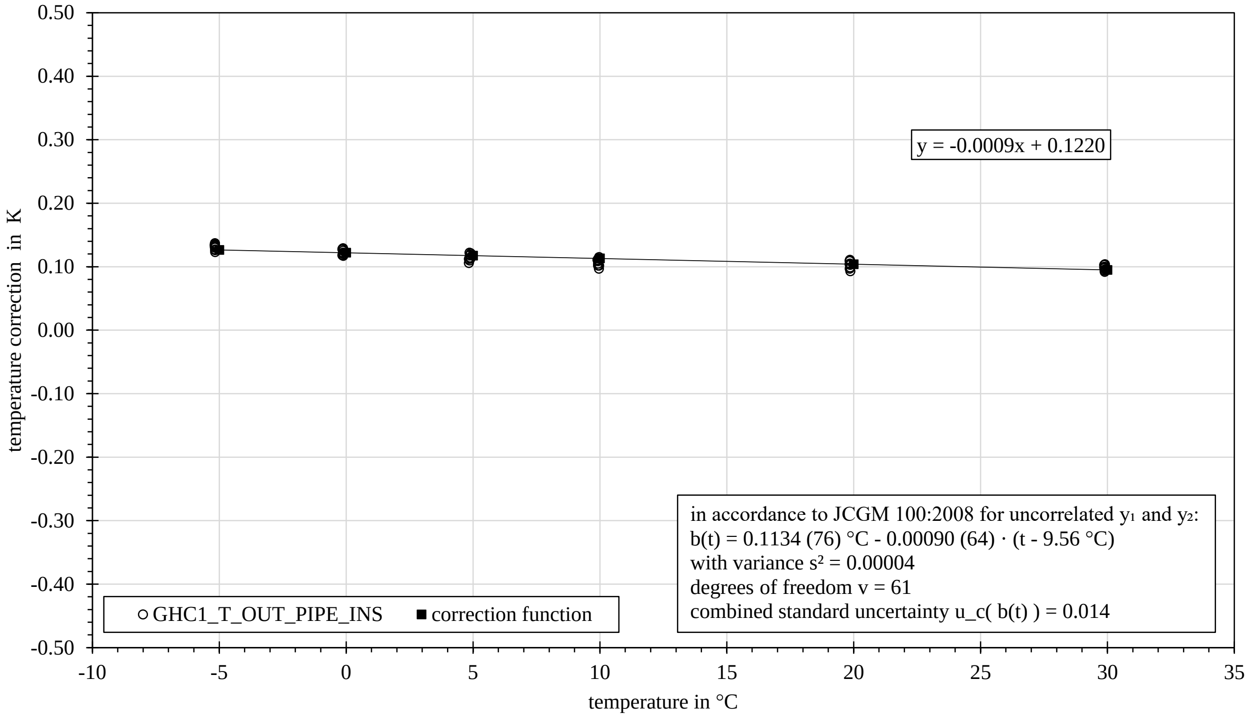

Figure 95: necessary correction of GHC1_T_OUT_PIPE_INS defined by calibration of the temperature sensor

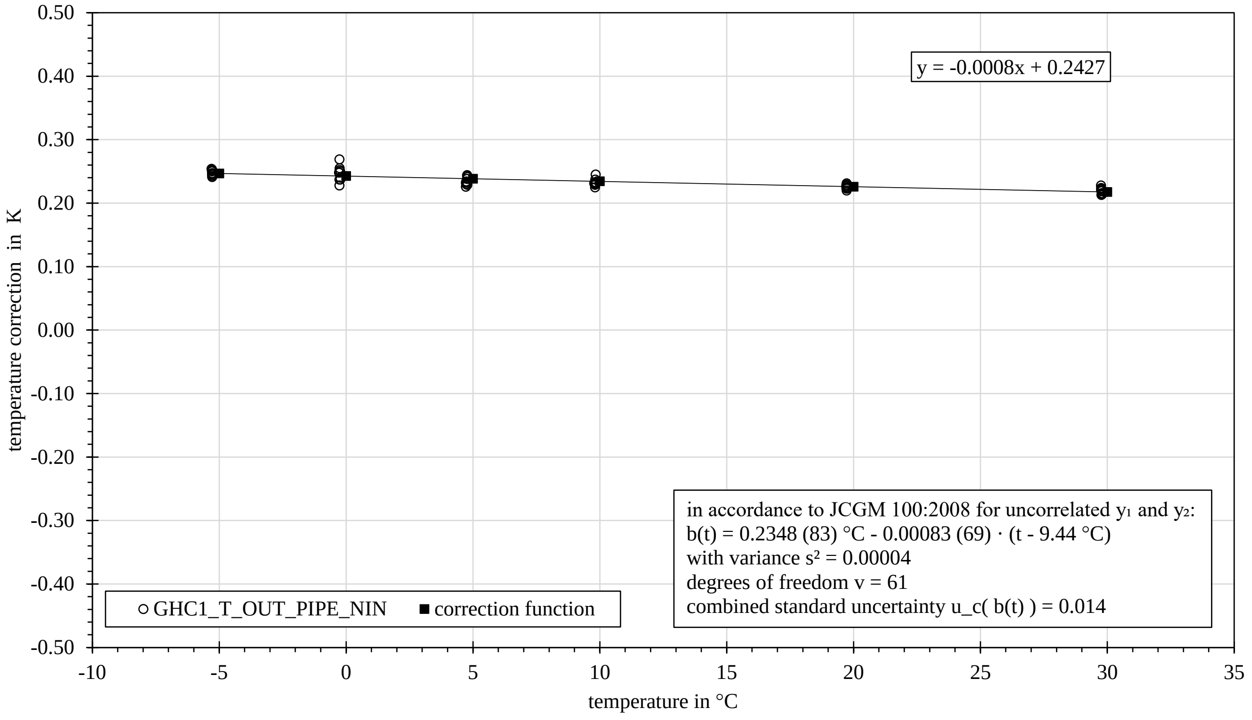

Figure 96: necessary correction of GHC1_T_OUT_PIPE_NIN defined by calibration of the temperature sensor

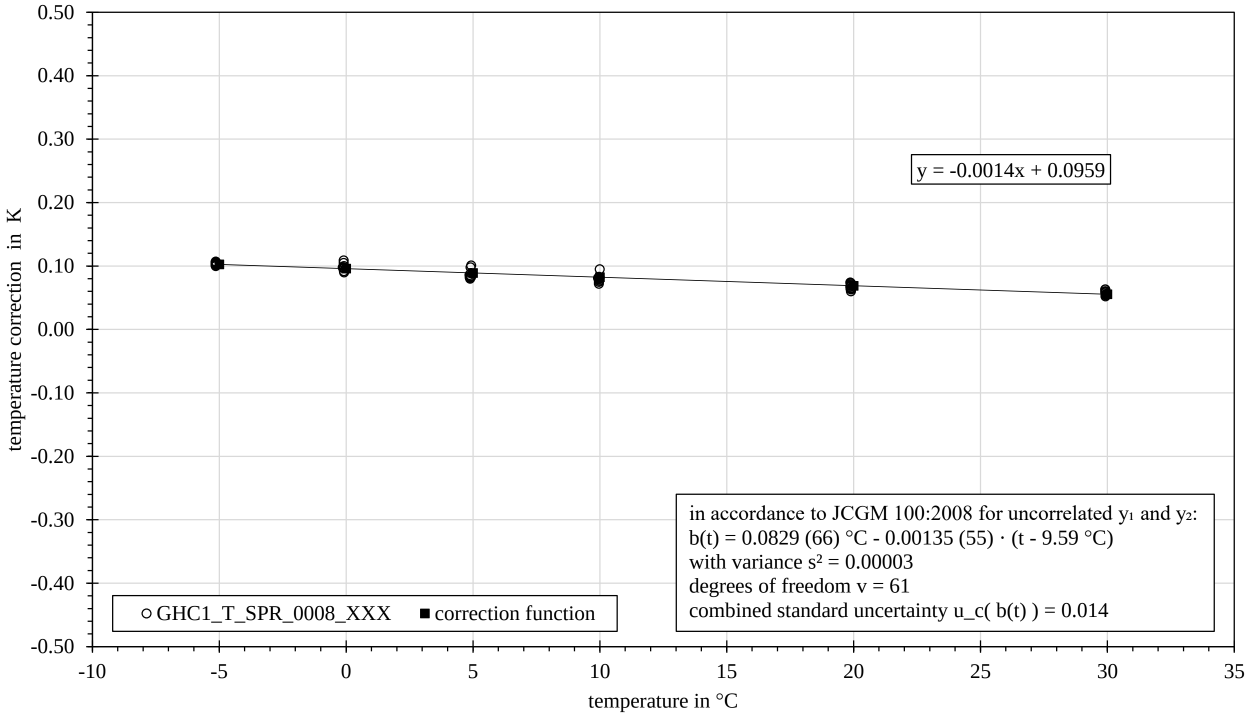

Figure 97: necessary correction of GHC1_T_SPR_0008_XXX defined by calibration of the temperature sensor

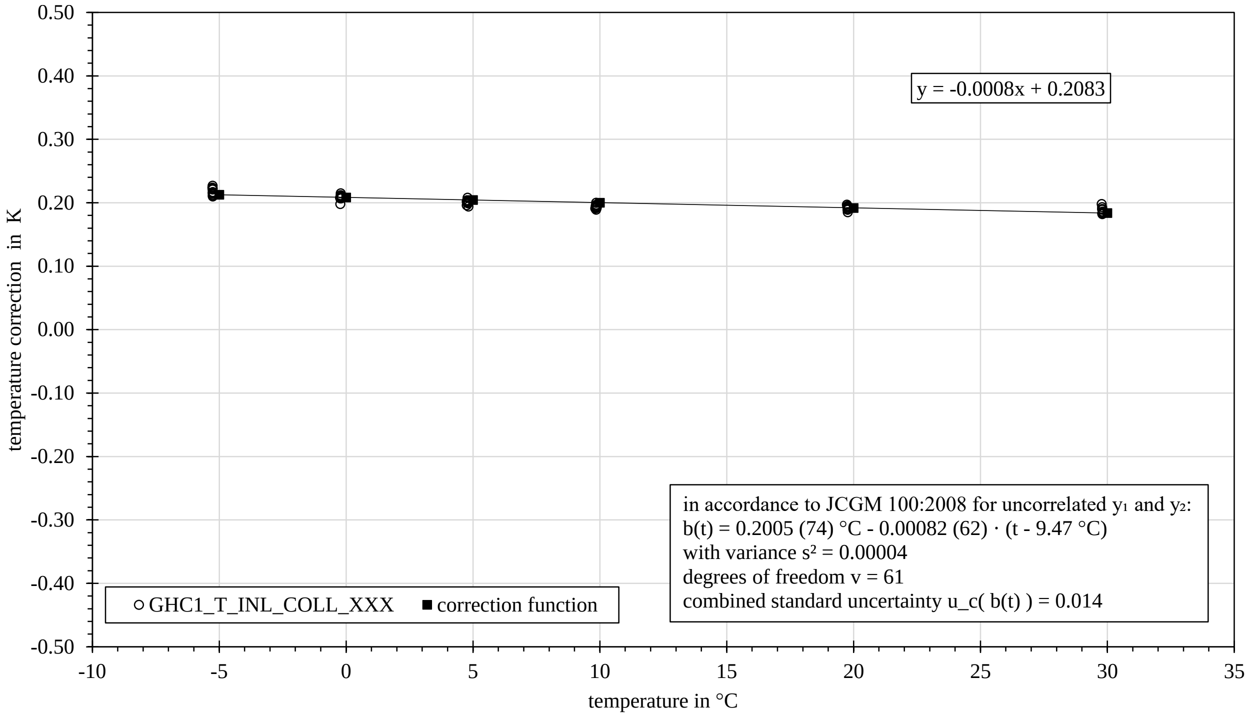

Figure 98: necessary correction of GHC1_T_INL_COLL_XXX defined by calibration of the temperature sensor

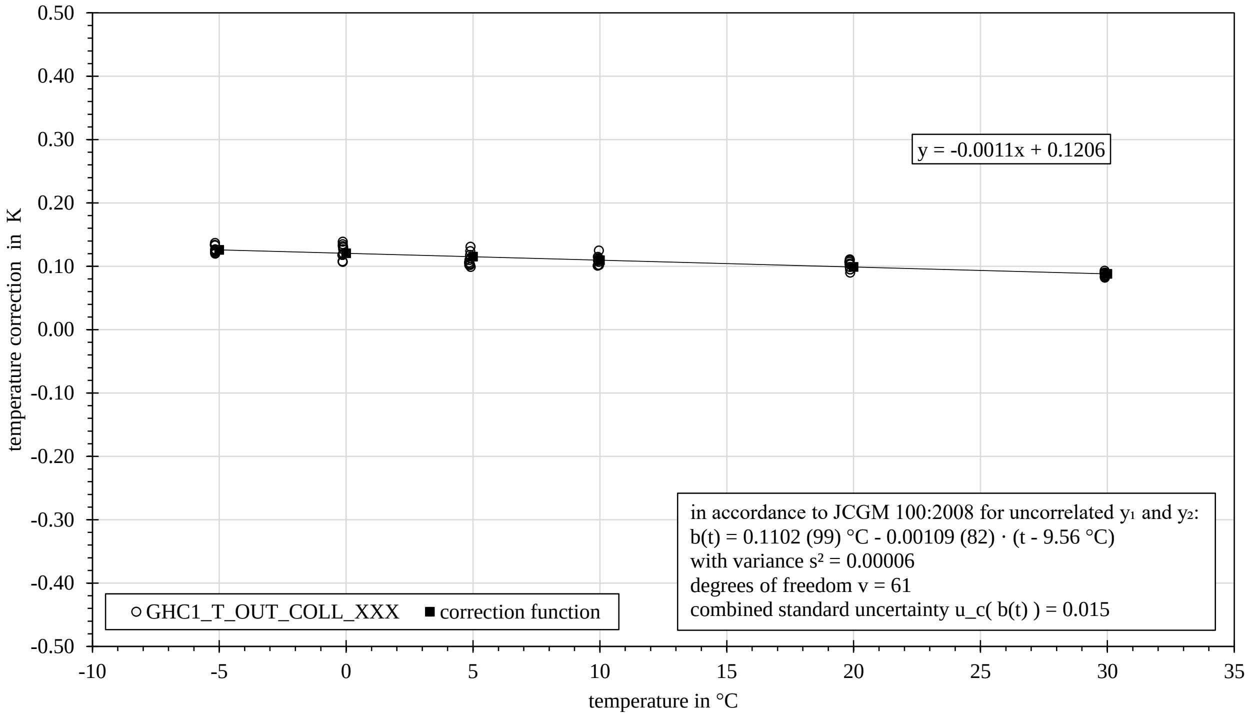

Figure 99: necessary correction of GHC1_T_OUT_COLL defined by calibration of the temperature sensor

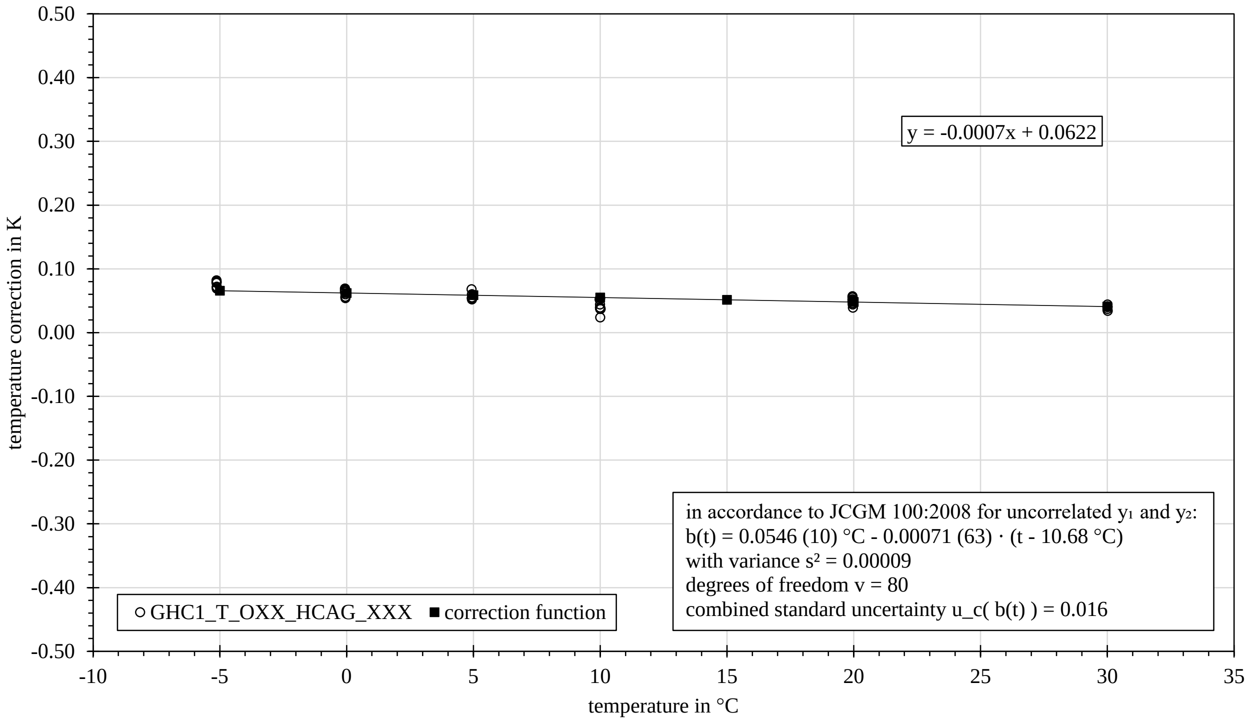

Figure 100: necessary correction of GHC1_T_OXX_HCAG defined by calibration of the temperature sensors

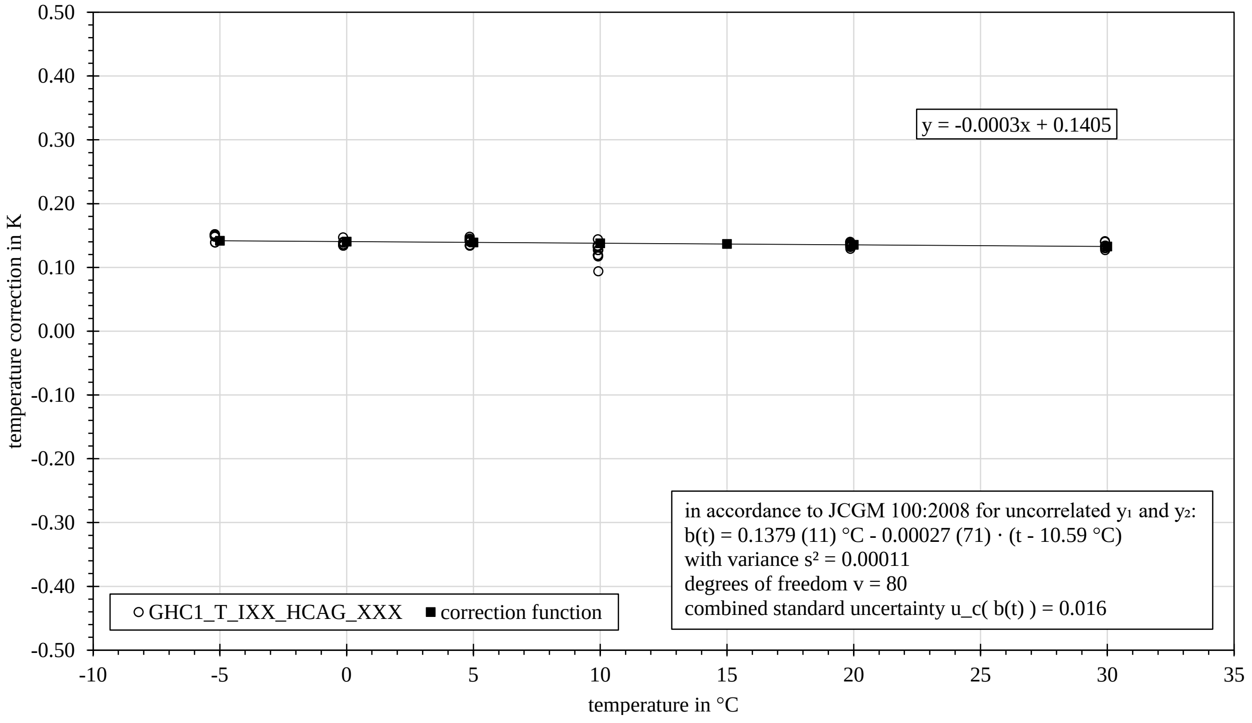

Figure 101: necessary correction of GHC_1_T_IXX_HCAG defined by calibration of the temperature sensors
